# Molecular mimicry of SARS-COV-2 antigens as a possible natural anti-cancer preventive immunization

**DOI:** 10.3389/fimmu.2024.1398002

**Published:** 2024-06-14

**Authors:** Concetta Ragone, Angela Mauriello, Beatrice Cavalluzzo, Ernesta Cavalcanti, Luigi Russo, Carmen Manolio, Simona Mangano, Biancamaria Cembrola, Maria Tagliamonte, Luigi Buonaguro

**Affiliations:** ^1^ Innovative Immunological Models Unit, Istituto Nazionale Tumori - IRCCS - “Fond G. Pascale”, Naples, Italy; ^2^ Lab of Clinical Pathology, Istituto Nazionale Tumori - IRCCS - “Fond G. Pascale”, Naples, Italy; ^3^ Clinical and Epidemiological Genetics Unit, Azienda Ospedaliera – University of Padua, Padua, Italy

**Keywords:** SARS-CoV-2, (TAA) tumor-associated antigen, T cell cross reactivity, molecular mimicry, cancer vaccine

## Abstract

**Background:**

In the present study we investigated whether peptides derived from the entire SARS-CoV-2 proteome share homology to TAAs (tumor-associated antigens) and cross-reactive CD8+ T cell can be elicited by the BNT162b2 preventive vaccine or the SARS-CoV-2 natural infection.

**Methods and results:**

Viral epitopes with high affinity (<100nM) to the HLA-A*02:01 allele were predicted. Shared and variant-specific epitopes were identified. Significant homologies in amino acidic sequence have been found between SARS-CoV-2 peptides and multiple TAAs, mainly associated with breast, liver, melanoma and colon cancers. The molecular mimicry of the viral epitopes and the TAAs was found in all viral proteins, mostly the Orf 1ab and the Spike, which is included in the BNT162b2 vaccine. Predicted structural similarities confirmed the sequence homology and comparable patterns of contact with both HLA and TCR α and β chains were observed. CD8+ T cell clones cross-reactive with the paired peptides have been found by MHC class l-dextramer staining.

**Conclusions:**

Our results show for the first time that several SARS-COV-2 antigens are highly homologous to TAAs and cross-reactive T cells are identified in infected and BNT162b2 preventive vaccinated individuals. The implication would be that the SARS-Cov-2 pandemic could represent a natural preventive immunization for breast, liver, melanoma and colon cancers. In the coming years, real-world evidences will provide the final proof for such immunological experimental evidence. Moreover, such SARS-CoV-2 epitopes can be used to develop “multi-cancer” off-the-shelf preventive/therapeutic vaccine formulations, with higher antigenicity and immunogenicity than over-expressed tumor self-antigens, for the potential valuable benefit of thousands of cancer patients around the World.

## 1 Introduction

The Coronavirus disease 2019 (COVID-19) pandemic has resulted in a dramatic global public health crisis (1) and the entire scientific community has focused the attention on strategies to combat such an emerging infection. In this quest, successful preventive vaccines have been developed and administered to millions of individuals worldwide inducing an effective protective immunity (2–4).

The Pfizer-BioNTech BNT162b2 vaccine is based on the entire Spike Glycoprotein of the virus to elicit neutralizing antibodies for blocking the interaction between the virus and the ACE-2 cellular receptor. The SARS-CoV-2 Spike protein is made of the N-terminal S1 subunit, for virus-receptor binding and a C-terminal S2 subunit responsible for virus-cell membrane fusion (5). The S1 subunit includes an N-terminal domain (NTD) and a receptor-binding domain (RBD). The latter directly binds to the peptidase domain (PD) of angiotensin-converting enzyme 2 (ACE2) (6, 7).

The BNT162b2 vaccine has been administered to >700 million of individuals and the efficacy against Covid-19 ranges between 86 and 100% across countries and populations, with a protection from severe diseases >96% (8–10). The response to anti-SARS-Cov-2 preventive vaccine shows high interpersonal variability at short and medium term (11), which is not dependent from the individual HLA allelic variants (12).

In addition to NAb response, T cell reactivity against SARS-CoV-2 epitopes has been reported in vaccinated subjects (13). Similar pattern of immune B and T cell responses have been reported in SARS CoV-2 infected individuals and specific target epitopes have been identified (14–19).

We have recently identified 59 immunogenic epitopes linked to the most prevalent HLA alleles in the sequence of the Spike protein included in the BNT162b2 mRNA vaccine. An established long-term CD8+ T cell memory response specific to 23/59 epitopes was found, with a strong immunodominance for NYNYLYRLF (HLA-A24:02) and YLQPRTFLL (HLA-A02:01) epitopes (20).

In the present study we wanted to verify whether the molecular mimicry between epitopes derived from the SARS-CoV-2 and publicly available TAAs can be identified along with cross-reacting CD8+ T cells. This would suggest that the SARS-CoV-2 pandemic has represented a natural “anti-cancer vaccination”, eliciting a spectrum of anti-viral T cell clones able to cross-react with tumor antigens. Such memory CD8+ T cells may be promptly recalled during the lifetime by cancer cells expressing TAAs similar or identical to the SARS-CoV-2 antigens and protect from cancer progression. This is possible because the degeneracy of the TCR in antigen recognition allows each single receptor to cross-react against similar antigens, recognizing at least 10^6^ different MHC-bound peptides (21, 22). Sequence and structure homologies between microorganism-derived antigens (viruses and bacteria) (MoAs) and tumor-associated antigens (TAAs) have been previously described (23). In particular, the homology should be localized in the amino acid residues at the central position of the peptides bound to the MHC-I groove, which directly interact with the T-cell receptor (TCR). Therefore, MoAs may elicit a T-cell response cross-reactive with TAAs and several experimental evidences have been reported so far (24–33). In particular, we have shown that several HLA-A*02:01-restricted cancer epitopes share sequence homology with peptides derived from human chronic viruses (i.e. Herpesviruses, papilloma, hepatitis B). Similarly, novel HLA-A*02:01-associated TAAs specific for hepatocellular carcinoma (HCC) (i.e. ISG15) with a high sequence homology to viral-derived antigens have been predicted (i.e. Calicivirus). Bioinformatics modelling showed that epitope’s pairs shared very similar 3D conformation, with an almost-identical pattern of contact with HLA and TCR. Finally, ex vivo immunization experiments demonstrate that PBMCs cross-react to the paired peptides (29–31). In particular, we have recently described peptides derived from HIV-1 sharing high sequence and conformational homology to TAAs derived from non-AIDS defining cancers (e.g. colon and breast cancers). Cross-reactive T-cells are identified only in HIV-positive patients, suggesting that a natural infection may turn out in a natural “preventive anti-cancer vaccine” (33).

More recently, we have mined homology between TAAs and antigens derived from Firmicutes and Bacteroidetes phyla, which account for 90% of gut microbiota (32). Sequence and structural homology emerged between several TAAs and HLA-A*02:01-restricted peptides derived from microbiota, which was particularly striking for MAGE-A1 KVLEYVIKV peptide. Importantly, we have been the first to report individual as well as cross-reactivity against paired MoAs and TuAs in both healthy subjects (HS) and cancer patients (CP), which is the founding evidence of the potential protective role of cross-reacting T cells on onset and progression of cancer (32–35). Based on such findings, here we searched for a molecular mimicry between SARS-CoV 2 antigens and TAAs expressed in several tumors. Significant sequence and conformational homologies have been found between SARS-CoV-2 peptides and TAAs expressed in different cancers. The viral epitopes were mostly derived from the Orf 1ab and the Spike proteins, which is included in the BNT162b2 vaccine. Furthermore, significant percentages of CD8+ T cell clones cross-reactive with the paired peptides have been found in infected and BNT162b2 vaccinated individuals.

## 2 Materials and methods

### 2.1 Sequence alignment of VOCs’ SARS-CoV-2 proteins and epitope prediction analysis

SARS-CoV-2 Spike protein encoded by the BNT162b2 vaccine and SARS-CoV2 proteins of the Wuhan, Alpha, Beta, Gamma, Delta and Omicron variants were downloaded ViralZone site (https://viralzone.expasy.org/9556). The entire sequences were analyzed with NetMHCpan4.1 tool (https://services.healthtech.dtu.dk/service.php?NetMHCpan-4.1), to predict the binding affinity.

Likewise, the peptides deposited at the Cancer Antigenic Peptide database (https://caped.icp.ucl.ac.be/Peptide/list) were used to interrogate NetMHCpan V.4.1 tool. Nonamer peptides for the first most prevalent MHC class I HLA-A*02:01 alleles (http://www.allelefrequencies.net) have been selected with a predicted affinity value <100nM.

### 2.2 BLAST homology search

The TAAs and SARS-CoV-2 derived peptide selected as SB according to NetMHCpan V.4.1 prediction tool, have been submitted to Basic Local Alignment Search Tool, BLAST (https://blast.ncbi.nlm.nih.gov/Blast.cgi) in order to identify sequence similarity between TAA and Viral epitopes predicted. Sequences with a homology of at least 4/9 identical residues along the sequence were considered significant.

### 2.3 Epitope modeling and molecular docking

The 3D structure of interaction between peptides and HLA-A*02:01 was generated using PyMol (PyMol Molecular graphics system, version 1.8.6.2) and Molsoft ICM (http://www.molsoft.com/, version 3.8–7d) software.

The PDB format of the complex between HLA-A*02:01 (1AO7), a viral peptide (TAX), and human T-cell receptor was downloaded from RCS Protein Data Bank (PDB) website (https://www.rcsb.org/structure/1AO7); the PyMol software was used to modify the TAX peptide sequence into the peptides analyzed in the present study. The Molsoft Mol Browser was used to generate the epitope modeling and molecular docking.

### 2.4 Peptide synthesis and solubilization

All peptides were synthesized at a purity of ≥90% (GenScript, Piscataway, NJ, USA). Lyophilized powders were reconstituted in DMSO Solution (CARLO ERBA Reagents S.r.l., Cornaredo, Italy) and diluted in 100 µM in ddH2O.

### 2.5 Sample collection and PBMCs isolation

Peripheral blood was obtained by venipuncture from a cohort of 56 healthcare workers enrolled at the National Cancer Institute “Pascale” in Naples, ITALY, upon signing an informed consent (**Supplementary Table 1**). All of them underwent the prescribed schedule of the Pfizer-BioNTech BNT162b2 vaccination (prime at Day 0; boost at day 21). Fresh human PBMCs were harvested after 2 weeks post-boosting dose, isolated by Ficoll-Hypaque density gradient centrifugation and cultured in RPMI 1640 medium (Life Technologies, Carlsbad, CA) supplemented with 2 mM l-glutamine (Sigma), 10% fetal bovine serum (Life Technologies) and 2% penicillin/streptomycin (5000 I.U./5 mg/ml, MP Biomedicals). Healthy donor samples were genotyped for HLA-A loci (Lab of Histocompatibility, Section of Cryopreservation and BaSCO, AORN Santobono-Pausilipon, Naples, Italy). Samples from HLA-A*02:01–positive individuals (n=20) were selected for downstream analyses.

### 2.6 Peptide binding affinity

Peptide binding affinity to HLA-A*02:01 molecule and BFA decay assays were per-formed for each candidate peptide. Human TAP-deficient T2 cell line (174xCEM.T2; ATCC CRL 1992™) was purchased from American Type Culture Collection (ATCC; https://www.atcc.org/) and cultured in Iscove’s modified Dulbecco’s medium (IMDM; Gibco Life Technologies) containing 25 mM HEPES and 2 mM L-Glut, supplemented with 20% fetal bovine serum (FBS; Capricorn Scientific GmbH), 100 IU/ml penicillin and 100 μg/ml streptomycin (Gibco Life Technologies). Cells were maintained at 37˚C in a humidi-fied incubator with 5% CO2. Briefly, T2 were seeded at 3.5 × 105 cells per well in 24 well plates and incubated 16 hours at 27°C with peptides (final concentrations: 5, 10, 20, 50 and 100 μM) in IMDM serum free medium. The next day, cells have been incubated for additional 2 hours at 37˚C. Following incubation, cells were harvested and centrifuged at 200 x g for 5 min. Subsequently, cells were washed twice with phosphate buffered saline (1X PBS; Gibco Life Technologies) and stained with R-PE conjugated anti human HLA A2 mono-clonal antibody (cat. 343306; BioLegend), for 30 min at 4°C, and analyzed with the At-tune™ NxT flow cytometer (Thermo Fisher Scientific). The fold increase was calculated using the following formula: FI = [mean fluorescence intensity (MFI) sample/MFI background, where MFI background represents the value without peptide. All the experiments were performed in triplicate.

### 2.7 IFN-γ ELISpot assay

PBMCs from HLA-A*02:01–positive individuals were harvested after 2 weeks post-boosting dose for IFN-γ ELISpot assay. Spike predicted peptides whit the best affinity for MHC class I HLA-A*02:01, KIADYNYKL_RBD_ and YLQPRTFLL_NTD_ were added at a final concentration of 10 μg/mL to 3×10^5^ PBMCs per well in 100 μL RPMI 1640 medium (Capricorn Scientific GmbH). PBMCs were cultured at 37°C in a humidified incubator with 5% CO2 for 20 hours. Stimulation with 10 μg/mL Phytohemagglutinin (PHA-K; Capricorn Scientific GmbH) was used as positive control; stimulation with 10 μg/mL LTDEMIAQY peptide was used as the negative control, RPMI 1640 medium (Capricorn ScientificGmbH) was used as background control. The plates were read with an AID EliSpot Reader Systems (AID GmbH, Strassberg, Germany). Determinations from triplicate tests were averaged. Data were analyzed by subtracting the mean number of spots in the wells with cells and medium-only from the mean counts of spots in wells with cells and antigen. Spot forming units (SFU) were calculated as the frequency per 10^6^ PBMCs.

### 2.8 pMHC multimer T cell staining

pMHC complexes were generated by combining purified disulfide-stabilized HLA-A*02:01 monomer (100 µg/mL) with 100 µM of Spike derived peptides for 30 min in PBS at room temperature (36). Then, pMHC complexes were centrifuged for 5 min at 3300_ g to sediment any aggregated MHC molecules. For each 100 µL pMHC, 9.02 µL SA-PE/Cy7 (BioLegend, 405206) or 18.04 µL (0.1 mg/mL stock, SA-BV421 (BioLegend, 405226), streptavidin conjugate was added and incubated for 30 min on ice. D-biotin (Sigma-Aldrich, St.Louis, MO, USA) was added at a final concentration of 25 µM to block any free binding sites. To stain for T-cell activity, PBMCs (2.5 x 10^6^) from healthy vaccinated donors were incubated with a pool of pMHC multimers (3 µL/multimer) and dasatinib (50 nM final, LC laboratories, D-3307) for 15 min at 20°C. Cells were then stained with antibodies anti-CD3 PE- Cy5 (1:100, BD, 15–0038-42), anti-CD8 FITC (1:100, BD, 555366) and LIVE/DEAD Fixable aqua(1:1000, Invitrogen) for 30 min on ice and washed twice in FACS buffer (PBS + 2% FCS).

Gating for CD3+/CD8+ T cells was performed on live cells, and binding to pMHCs was assessed by measuring specific fluorescence associated with each individual pMHC. Samples were acquired on a flow cytometer (Attune NxT, Thermo Fisher).

### 2.9 MHC I Dextramer preparation and T cell staining

*MHC I Dextramer* complex (Immudex) were generated by combining purified disulfide-stabilized HLA-A*02:01 monomer (3 µM), with 100 μM peptide for 18°C for 48 h; subsequently MHC I-peptide monomer (1 µM), were loaded onto U-Load Dextramer and incubated for 30 min in dark. In particular all the MHC I -SARS-CoV -2 derived peptides were loaded onto U-Load Dextramer PE conjugated; whereas MHC I -TAA peptides were loaded onto U-Load Dextramer FITC conjugated. To stain for T-cell reactivity, 3 x 10^6^ PBMCs from vaccinated donors, were stimulated for 3 days with viral peptides. After stimulation PBMCs were harvested and incubated with a pool of MHC I Dextramer complex for 10 min at 37°C in dark; D-biotin (Sigma-Aldrich, St.Louis, MO, USA) was added at a final concentration of 25 μM to block any free binding sites. Cells were then stained with antibodies anti-CD3 Super Bright 436 (1:100, Invitrogen, 62–0037-42), anti-CD8 PE- Cy7 (1:100, Biolegend, 300914) and LIVE/DEAD Fixable aqua (1:1000, Invitrogen) for 30 min on ice and washed twice in FACS buffer (PBS + 2% FCS). Gating for CD3^+^/CD8^+^ T cells was performed on live cells, and binding to *MHC I Dextramer* was assessed by measuring specific fluorescence associated with each individual *MHC I Dextramer*. Samples were acquired on a flow cytometer (Attune NxT, Thermo Fisher).

### 2.10 Data processing and statistical analysis

T cell recognition data, determined by DNA-barcoded pMHC multimers analysis and Barracoda software, was plotted using RStudio version 4.1.0. For statistical analysis, data was assumed to have a non-Gaussian distribution and non-parametric tests were therefore used. Wilcoxon signed rank test was used for single paired comparisons and the Mann-Whitney test was used for unpaired comparisons. The p-values are indicated in figure legends. Plots were generated using GraphPad Prism version 9.1.2 (GraphPad Software Inc., USA).

## 3 Results

### 3.1 Sequence alignment of VOCs’ SARS-CoV-2 spike proteins

The entire proteome of the reference SARS-CoV-2 Wuhan isolate as well as Alpha, Beta, Gamma, Delta and Omicron variants of concern (VOC), were downloaded from the ViralZone site (https://viralzone.expasy.org). The protein sequences, covering the entire Spike protein (1.273 aa), were selected and aligned to identify identities and differences between the 6 VOCs. Most of the mutations are located in the first 650 positions, corresponding to the S1 region, which include the most antigenic regions of the spike protein. In particular, indels are found only in the first 250 positions at the NH_2_ terminus of the Alfa, Beta, Delta and Omicron proteins (**Supplementary Table 2**). The identity matrix shows that the Omicron sequence is the most diverse from all other VOCs. Indeed, while the VOCs’ sequence homology is, on average, 98.74% (98.52 – 98.94), the Omicron sequence has an average homology with all other VOCs of 97.3% (97.08 – 97.4) (**Supplementary Table 3**).

### 3.2 Peptide prediction in the SARS-CoV-2 spike protein

**Figure 1:**
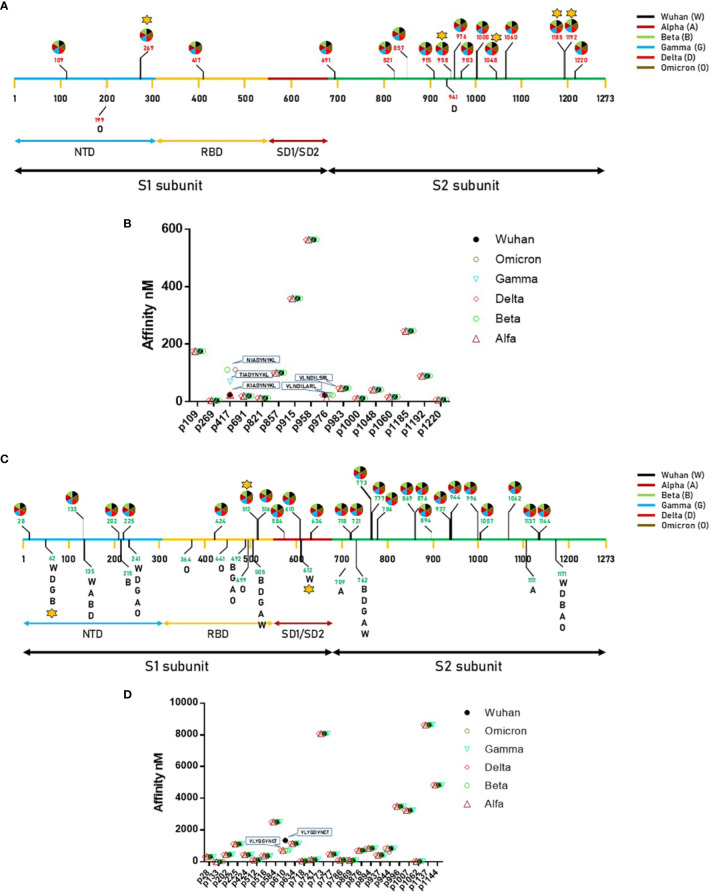
Predicted epitopes in the SARS-CoV-2 Spike protein. **(A)** Position of the epitopes predicted to be Strong Binders (SBs) and indication of the VOC in which they have been found.; **(B)** Affinity value is indicated for each of the predicted SBs. **(C)** Position of the epitopes predicted to be Weak Binders (WBs) and indication of the VOC in which they have been found.; **(D)** Affinity value is indicated for each of the predicted WBs. The star indicates the epitopes for which T cell reactivity has been previously published.

In order to verify whether the observed mutations reflected in predicted HLA-A*02:01-associated epitopes, the individual spike proteins were analyzed with the NetMHCpan software. The analysis returned 18 predicted strong binding peptides (SB), all shared among the VOCs with the exception of one identified only in the spike of the Omicron variant (199 – KIYSKHTPV – 207) and one in the spike of the Delta variant (941 – SALGKLQNV - 949) (**Supplementary Table 4**; **Figure 1A**). Some of the shared predicted strong binding peptides are characterized by different linear aa sequence, which has an impact on the predicted affinity to the HLA-A*02:01 allele. In particular, at position 417, while the peptides derived from Wuhan, Alpha and Delta VOCs have an affinity of 23.08 nM, the one derived from the Gamma VOC has an affinity of 69.17 nM and the ones derived from the Beta and Omicron VOCs have an affinity of 110.85 nM. Similarly, at position 976, while the peptides derived from all VOCs have an affinity of 22.77 nM, the one derived from the Alpha VOC has an affinity of 12.95 nM (**Figure 1B**). Regarding the predicted weak binding peptides (WB), the analysis returned 39 peptides, of which 25 are shared among all the VOCs and 14 are identified in single or multiple spikes of the variants (**Supplementary Table 5**; **Figure 1C**). Overall, 7 new predicted WBs have been identified in VOCs other than Wuhan. Of these, 1 is shared at position 492, 3 are unique to the Omicron, 2 unique to Alpha and 1 unique to Beta variants (**Supplementary Table 6**). Interestingly, the Delta variant does not show any new predicted WB. All VOCs loose the predicted WB at position 612 of the Wuhan variant (**Figure 1C**). The predicted shared WBs, even though are characterized by different linear aa sequence, they show identical predicted affinity to the HLA-A*02:01 allele. The only exception is represented by the peptide at position 610, which shows a significant improvement in affinity in all VOCs compared to the Wuhan variant (733.09 vs. 1342.97 nM) (**Figure 1D**). For all the subsequent analyses, only SB (nr. 12) and WB (nr. 4) with affinity <100nM have been further considered (**Supplementary Tables 4** and **5**).

**Figure 2:**
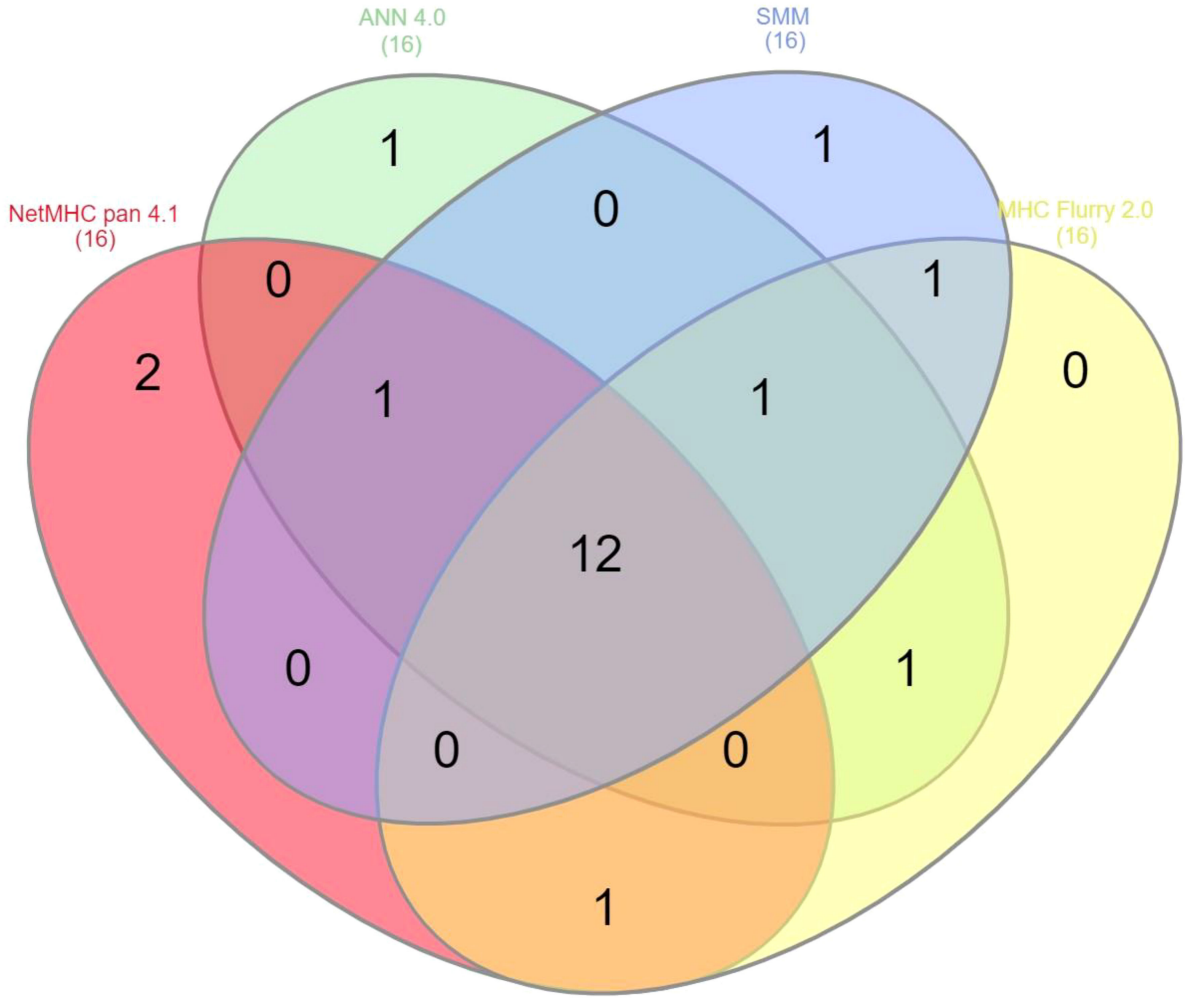
Compared prediction of SB+WB by all algorithms. The prediction of SB and WB spike epitopes predicted by NetMHC – Pan4.1 (<100nM) was assessed also with the indicated algorithms. Unique and shared predicted epitopes in the first 16 positions in all methods are indicated.

In order to validate the 16 selected SB+WB epitopes, the same prediction analysis was performed using additional three algorithms (ANN 4.0, SMM and MHC Flurry 2.0). The results showed that 2 out of 16 epitopes were predicted only by NetMHC-Pan4.1 and 12 of them were predicted by all four algorithms (75%) (**Figure 2**; **Supplementary Table 7**).

### 3.3 T cell reactivity to predicted spike epitopes

We have recently assessed T cell reactivity against 59 epitopes predicted in the Spike protein associated to different HLA alleles, covering both the S1 and S2 subunits. In particular, 11 were associated with the HLA-A*02:01 and only three were located in the S1 subunit, namely VTWFHAIHV at position 62 and YLQPRTFLL at position 269 in the NTD region; VLSFELLHA at position 512 in the RBD region. The results show that a long-term CD8+ T cell memory response is observed for 23 peptides and the strongest immunodominance is found for NYNYLYRLF (HLA-A*24:02), YLQPRTFLL (HLA-A*02:01) epitopes (20).

In the present analysis, we have found that the latter has the highest affinity to the HLA-A*02:01 allele (4.3 nM), supporting the previous finding on the immunodominance. In order to assess the T cell response to an additional SB epitope associated to the HLA-A*02:01 allele within the S1 subunit, we selected the KIADYNYKL SB peptide at position p417 in the RBD region (23.08 nM).

**Figure 3:**
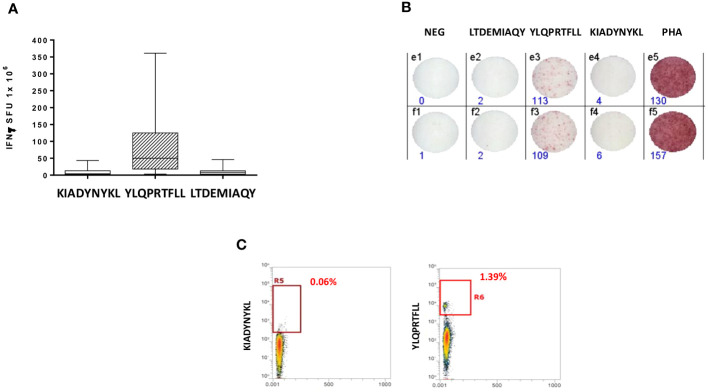
Epitope-specific CD8+ T-cell clones for epitopes derived from the SARS-CoV-2 Spike protein. **(A)** Elispot assay. PBMCs from 20 HLA-A* 02:01 positive vaccinated donors were stimulated O/N with the specific HLA-A*0201 predicted Spike Epitopes. SFU = IFNγ spot forming units. **(B)** Elispot assay for a single subject; **(C)** Box plot showing in a single individual the higher presence of CD8+ T cells specific for YLQPRTFLL than KIADYNYKL after pMHC staining.

An IFN-γ ELISpot assay was performed on PBMCs from all vaccinated healthcare workers, comparing the T cell reactivity to the KIADYNYKL peptide and the immunodominant YLQPRTFLL peptide using the LTDEMIAQY peptide as the negative control. All subjects had variable levels of circulating T CD8+ cells reacting mainly to the YLQPRTFLL peptide (0–356 SFU, with an average of 87.9), in agreement with our previous finding. On the contrary, the reactivity to the additional KIADYNYKL_RBD_ peptide did not differ from the one to the negative control (mean 9.8 vs. 10.8 SFU) (**Figures 3A, B**; **Supplementary Figure 1**). Such results were confirmed by an MHC-class I tetramer staining, which showed a percentage of circulating CD8+ T cells specific for the YLQPRTFLL peptide significantly higher compared to KIADYNYKL (average 1,23% and 0,18% respectively) (**Figure 3C**).

### 3.4 Identification of spike peptides homologous TAA epitopes

In order to verify whether such strong T cell reactivity elicited by the YLQPRTFLL peptide could provide a preventive protection against cancer development, we searched for sequence homology with all published tumor associated antigens (TAAs) at the Cancer Antigenic Peptide database. The BLAST search did not return significant homology with any known TAA. We subsequently searched for all other 56 SB and WB peptides predicted in the Spike protein and 10 partial homologies were returned. Of these, five were in the SP1 subunit along the sequence (two in the NTD, two in the RBD and one in the SD1/SD2 regions), and five in the SP2 subunit. Only the latter were classified as SBs, corresponding to CSNK1A1, GnTV, CLPP, Tyrosinase, STEAP1. Among these 10 peptides, 6 are common to all the VOCs, 1 is only in the Beta, 3 in the Omicron and 1 in all but the Wuhan (**Table 1**).

**Table 1:** Sequence homology between spike epitopes and TAAs.

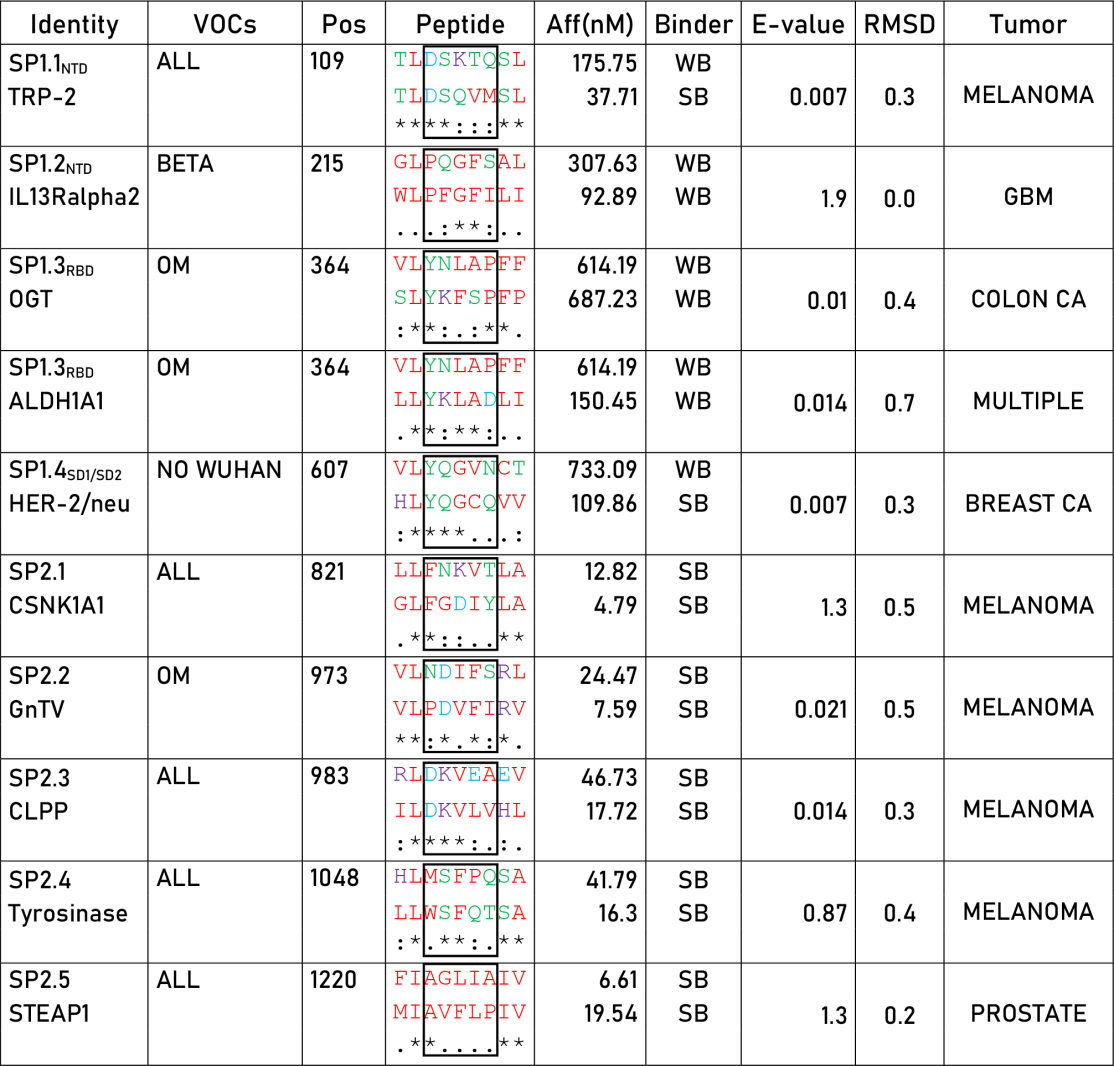

The spike epitopes have been aligned to homologous TAAs, as identified via BLAST search. Aligned aminoacid sequences are shown for each epitope pair. Chemical properties are indicated by color code. Asterisk (*) indicates identity between the epitopes. Full stop (.) indicates residues of the same chemical property. Colon (:) indicates residues of different chemical property. The affinity for each peptide is reported as well as the tumor expressing the TAA.

Red: non polar.

Green: polar.

Purple: positive.

Blue: Negative.

Given that the core region of the nonamer bound to the HLA molecule is the one prevalently involved in the interaction with the α and β chains of the TCR, we analyzed this region for each of the paired peptides (p3 – p7). In particular, we focused on the predicted SBs. The amino acid residues of the SP2.1/CSNK1A1 pair shows identity only in p3, similar chemical properties in p6 and p7, different properties in p4 and p5. The SP2.2/GnTV pair shows identity in p4 and p6, similar chemical properties in p5, different properties in p3 and p7. The SP2.3/CLPP pair shows identity in p3 - p5, similar chemical properties in p7, and different properties in p6. The SP2.4/Tyrosinase pair shows identity only in p4 & p5, similar chemical properties in p3 and p7, different properties in p6. The SP2.5/STEAP1 pair shows identity only in p3, similar chemical properties in p4 - p7 (**Table 1**). Binding to HLA-A*02:01 was experimentally confirmed using the TAP-deficient T2 cells (**Supplementary Figure 2**).

### 3.5 Conformational and interaction analysis of the paired epitopes

**Figure 4:**
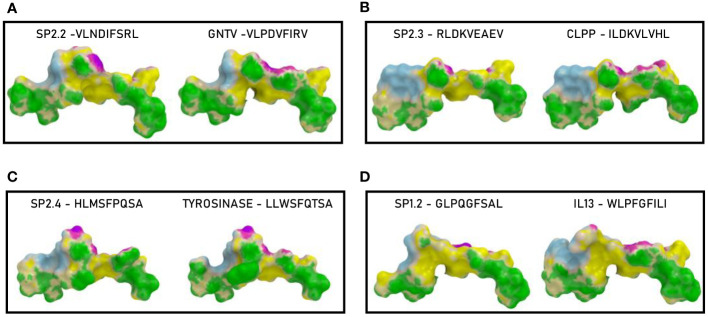
Predicted 3D conformation of SPIKE peptides and paired TAAs. The conformation of the SARS-CoV-2 spike-derived epitopes (SP) and paired TAAs bound to the indicated HLA-A*02:01 molecule is shown. The paired epitopes are indicated in each box **(A–D)**. The prediction was performed using as template the publicly available crystallized structure (PDB https://www.rcsb.org/structure/1AO7). Green areas = contact points with HLA molecule; light Blue areas = contact points with the TCR α chain; Violet areas = contact points with the TCR β chain.

In order to further evaluate the conformation similarity of the paired peptides, bioinformatics analyses have been performed by molecular docking of both peptides together with the HLA and the TCR molecules. The results showed that three of the peptides from the spike’s SP2 region share a very high conformation similarity to TAAs. In particular, the SP2.2 –VLNDIFSRL_973-OM_ is highly homologous to the GnTV – VLPDVFIRV; the SP2.3 – RLDKVEAEV_983-ALL_ is highly homologous to the CLPP – ILDKVLVHL; the SP2.4 – HLMSFPQSA_1048 – ALL_ is highly homologous to the Tyrosinase – LLWSFQTSA. Indeed, the comparison analysis of the spike peptides with the homologous TAAs showed high similar conformation and contact areas to the HLA molecule as well as to α and β chains of the TCR (**Figures 4A-C**). On the contrary, all other peptides from the SP1 and SP2 regions showed poor conformation similarity to TAAs (**Figure 4D**; **Supplementary Figures 3-4**).

**Figure 5:**
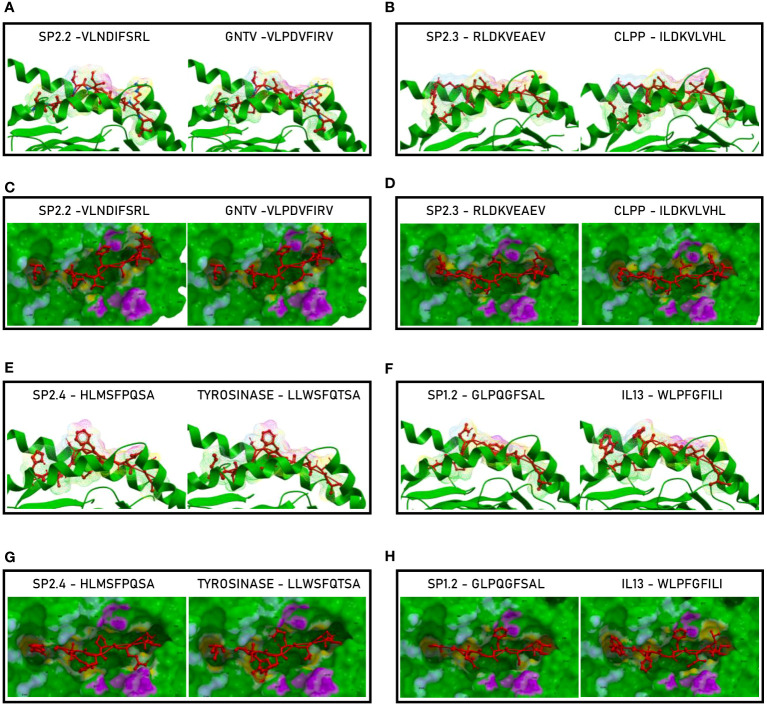
Molecular mimicry of spike epitopes and TAAs: interaction with the HLA molecule. The paired epitopes are shown bound to the HLA-A*02:01 molecule. The TCR facing residues are presented to the TCR αβ chains with the same conformation **(A, B, E, F)**. The footprint of the paired epitopes on the HLA molecule is shown and the contact points are highlighted in yellow **(C, D, G, H)**.

Such observations were confirmed by the analysis of the paired peptides positioned in the HLA peptide-binding groove (**Figure 5**). Indeed, while the SP2.2, 2.3 and 2.4 peptides and the paired TAAs show very high similarity (**Figures 5A–D, E, G**), the SP1.2 is confirmed to be significantly different from the paired IL-13 TAA in the interaction with the HLA peptide-binding groove. This is particularly obvious looking at the footprint of the two peptides on the HLA groove (**Figures 5F, H**; **Supplementary Figures 5-8**).

**Figure 6:**
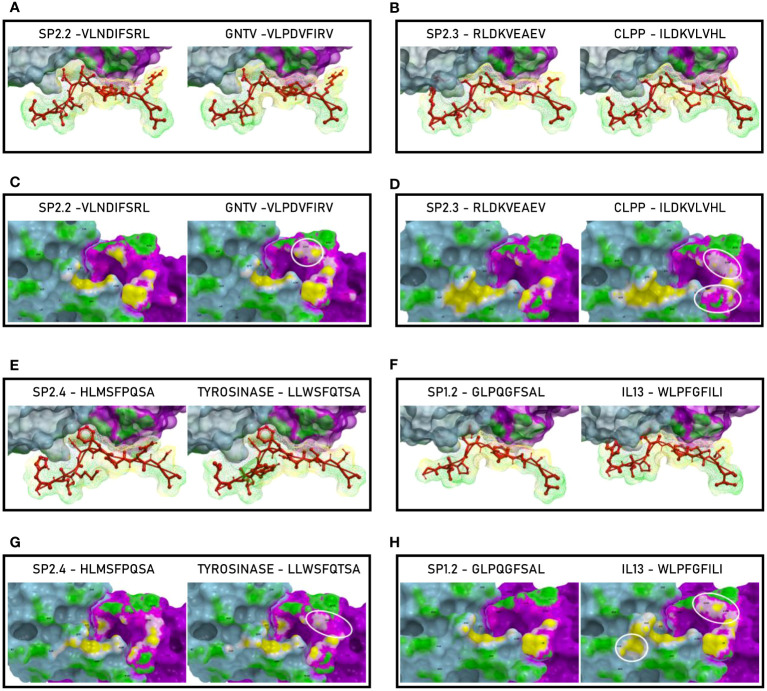
Molecular mimicry of spike epitopes and TAAs: interaction with the TCR molecule. The paired peptides are shown in contact with the TCR αβ chains **(A, B, E, F)**. The footprint of the paired epitopes on the TCR αβ chains is shown and the contact points are highlighted in yellow (α chain) and in pink β (chain). For each pair, differences in the contact areas are circled **(C, D, G, H)**.

The analyses of the interaction between the paired peptides and α and β chains of the TCR provided a further perspective. As reasonably predictable, none of the paired peptides show identical footprints on the TCR chains (**Figure 6**). However, the SP2.2, and 2.4, and the paired TAAs, show minor differences only in the contact areas of the p7 residue (S vs. I, for the SP2.2/GnTV pair; Q vs. T for the SP2.4/Tyrosinase) with the G_100_ and G_101_ residues of the β chain (**Figures 6A, G**). The SP2.3/CLPP pair shows more pronounced differences in the contact areas of the p7 and p8 residues (AE vs. VH) with the L_98_, G_100_ and G_101_ residues of the β chain (**Figure 6D**). No differences in the contact areas with the α chain of the TCR were observed for the three pairs. On the contrary, the less homologous SP1.2/IL13 pair, in addition to differences observed for the other peptides, shows a major difference in the contact area of the p1 residue (G vs. W) with the G_28_, S_29_, Q_30_ residues of the α chain of the TCR (**Figure 6H**; **Supplementary Figures 9–12**).

### 3.6 T cell cross-reactivity to paired peptides

**Figure 7:**
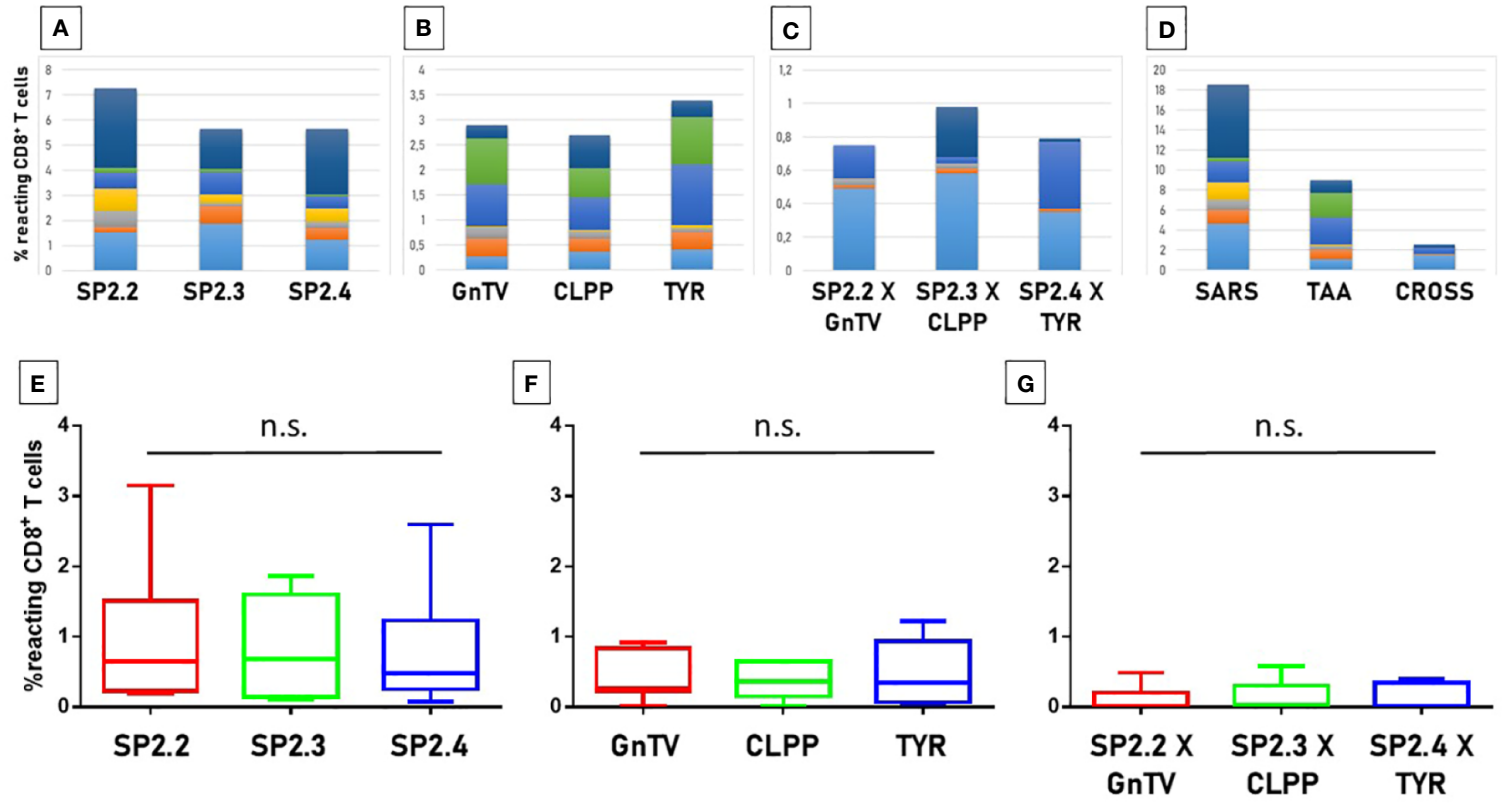
T cell reactivity to spike epitopes and paired TAAs. PBMCs from HLA-A*02:01 positive vaccinated were analyzed by tetramer-staining with the indicated epitopes. Results of reactivity to individual epitopes are shown **(A, B, E, F)**; Results of cross-reactivity to both epitopes are shown **(C, G)**. Cumulative response in all subjects to individual epitopes and cross-reactivity is shown **(D)**. n.s. means not statistical significant.

In order to evaluate if the paired Spike and TAAs peptides with high sequence and conformational homologies were recognized by cross-reacting CD8^+^ T cells, an MHC I Dextramer complex-staining was performed. PBMCs from 7 vaccinated donors were stimulated ex vivo for 3 days with the Spike peptide and then incubated with MHCI Dextramer complexes loaded with either the same viral peptide or the homologous TAA. The results showed a substantial percentage of circulating reacting CD8^+^ T cells specific to the SP2.2, 2.3, 2.4 spike peptides, with average values not statistically different (1.04%, 0.8%, 0.8% on average, respectively) (**Figure 7A**). A lower but significant percentage of circulating reacting CD8^+^ T cells specific to the homologous TAAs spike peptides were observed. Also in this case, the average values were not statistically different (0.4%, 0.48%, and 0.38% on average, respectively) (**Figure 7B**). Interestingly, a CD8^+^ T cell cross-reactivity was observed for the three paired peptides, with average values of 0.11 – 0.14% (**Figure 7C**). The T cell reactivity against the individual peptides as well as the cross-reactivity showed an inter-subject relevant variability. In general, no cross-reactivity was observed in samples showing a percentage of CD8^+^ T cells reacting against either the Spike or the TAA lower than 0.2%. *Reactivities against the negative control (namely a scrambled peptide) was between 0.0% and 0.05% in all tested subjects (0.027% on average; ± 0.0023 standard deviation). For all subjects the response to each SARS and TAA was statistically significant compared to the natural background (p <0.05).*

### 3.7 TCR α and β chains of reacting T cells

**Figure 8:**
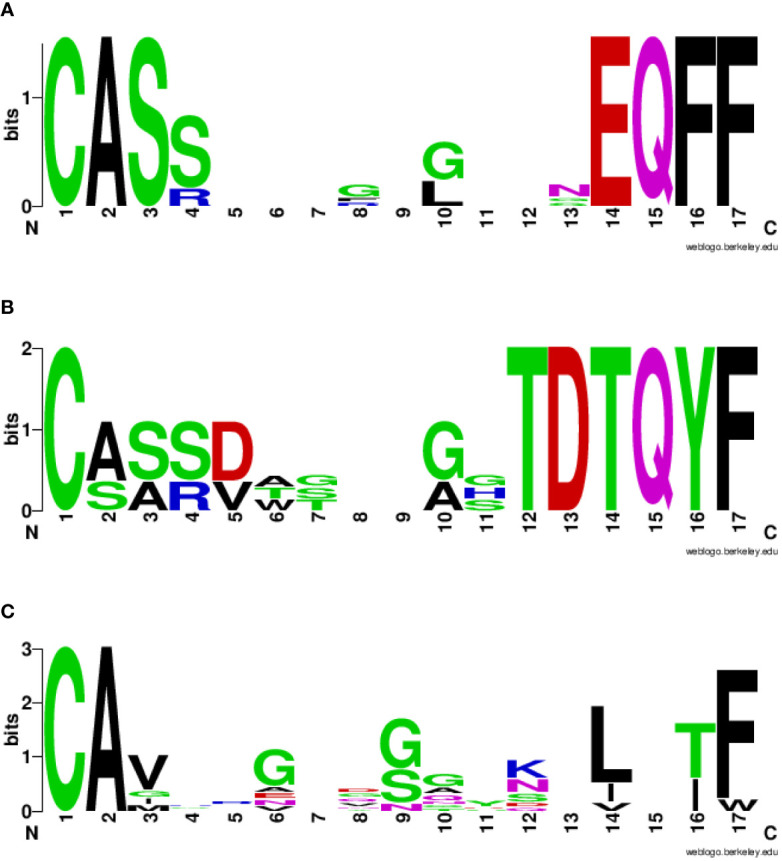
Consensus CDR3 sequences of the αβ chains reacting to paired epitopes. The TRAV/TRAJ and the TRBV/TRBJ sequences described in literature to bind the SP2.4 or the TYR epitopes have been piled up to generate the Seq Logo. SeqLogo from the β CDR3 TRBJ2–1 **(A)** and 2–3 **(B)** binding both epitopes are indicated. SeqLogo from the α CDR3 TRAJ **(C)** binding both epitopes are indicated. The height of the letters indicates the frequency of the specific amino acid residue in that specific position of the epitope.

The VDJdb database was interrogated for searching TCR α and β chains (CDR3 regions) already identified to bind the spike and the homologous TAA peptides. The search returned results only for two spike peptides, namely the SP2.3 and 2.4, and one of the TAA peptides, namely the TYR homologous to SP2.4. While a single α and β CDR3 region has been identified to bind the SP2.3 peptide, at least 4 have been found to bind the SP2.4 peptide. Likewise, the homologous TYR TAA has been described to be bound by 6 independent α and β CDR3 regions (https://vdjdb.cdr3.net/search) (**Table 2**). As expected, none of the combinations binding the individual peptides is identical. However, it is of interest the finding that the same J regions of the β CDR3 (TRBJ2–1 and 2–3) are identified in the TCR binding both the SP2.4 and the homologous TYR TAA. The sequences of the CDR3 are not identical, but the consensus show high conservation at the NH_2_- and COOH- termini of the sequence (**Figures 8A, B**). On the contrary, the J regions of the α chain (TRAJ) do not show any conservation among the TCR binding the paired epitopes, except for the canonical residues C_1_ - A_2_ and F_17_ at the NH_2_- and COOH- termini of the sequence, respectively (**Figure 8C**) (37).

**Table 2:** CDR3 sequence of the α and β chains binding spike and TAA epitopes.

	CDR3 α chain			CDR3 β chain		
SP2.4	CA---G-DSNYQ-L-IW	TRAV27	TRAJ33	CASSQDMR-AG-GPNEQFF	TRBV4-3	TRBJ2-1
	CAI-YN-QGG-KLI--F	TRAV12-2	TRAJ23	CASSTTF--SG-GWSEQFF	TRBV11-3	TRBJ2-1
	CAVSGGTGGG-NKL-TF	TRAV8-4	TRAJ10	CSARDWT---A-STDTQYF	TRBV20-1	TRBJ2-3
	CAGRRG-YGQ-NFV--F	TRAV13-1	TRAJ26	CASSS-G--QG-FFYEQYF	TRBV7-9	TRBJ2-7
TYR TAA	CAV-RGRVSTDK-L-IF	TRAV8-6	TRAJ34	CASSQDQ-----GITEAFF	TRABV4-1	TRBJ1-1
	CA---GKVSGYSTL-TF	TRAV12-2	TRAJ11	CASSQEGGLQGTFVYGYTF	TRABV4-2	TRBJ1-2
	CAVAK---GQ-KLL--F	TRAV3		CASSPSTG-GL-FYNEQFF	TRABV7-9	TRBJ2-1
	CAVNWEEQGG-SYIPTF	TRAV12-2	TRAJ6	CASRVS-G-SL-SYNEQFF	TRABV27	TRBJ2-1
	CAMSRG-GGA-DGL-TF	TRAV12-3	TRAJ45	CASSTPG--QG-A-GEQFF	TRABV6-5	TRBJ2-1
	CAVRDAHNNAGNML-TF	TRAV1-1	TRAJ39	CASSVTSS-RG-GTDTQYF	TRABV9	TRBJ2-3
	CA---VMDSSYK-L-IF	TRAV1-2	TRAJ12	CASSDAG---G-HTDTQYF	TRABV6-4	TRBJ2-3

The CDR3 sequences of the α and β chains binding the SP2.4 spike and the homologous TYR TAA epitopes are shown. Data are from the VDJdb database (https://vdjdb.cdr3.net/).

### 3.8 Peptide prediction in the SARS-CoV-2 proteins outside the spike

Considering that most of the individuals, even if vaccinated with the spike protein, have been and are possibly exposed to the infectious virus, a T cell response against all possible epitopes in the other viral proteins can be elicited. Therefore, all other protein sequences of the SARS-CoV-2 VOCs were analyzed with the NetMHCpan software, to predict potential HLA-A*02:01-associated epitopes.

**Figure 9:**
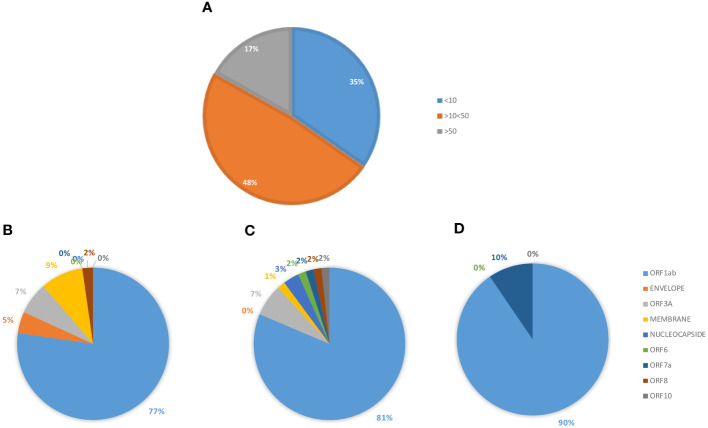
Predicted epitopes in the SARS-CoV-2 proteome. The SBs predicted from the full SARS-CoV-2 proteome, except for the spike protein, are summed up. The percentage of SBs, according the affinity value (nM) is reported **(A)**. The percentage of SBs, in each subgroup, is shown according to each of the viral proteins from which they are derived: <10 nM **(B)**; >10<50 nM **(C)**; >50 nM **(D)**.

The analysis returned a variable number of predicted strong and weak binding peptides (SB and WB), according to the length of each protein sequence. Considering the two extremes, the ORF1ab (7096 aa) and the ORF10 (38 aa), the first showed the highest number of both SB (101) and WB (61) and the latter the lowest number of both SB (1) and WB (0). 98% the predicted binding peptides are shared among the VOCs with the exception of two SBs identified in the ORF1ab (FLARGVVFM_OMICRON_, TIIQTIVEV_ALPHA_) and one WB identified always in the ORF1ab (TIWFLLLSV_ALPHA_) (**Table 3**, **Supplementary Tables 8**, **9**). More than 35% of the SBs are predicted to have an extremely high affinity (<10nM) and 77% of these are predicted in the ORF1ab; 48% are predicted to have a very high affinity (>10<50nM) and 81% of these are predicted in the ORF1ab; 17% are predicted to have a high affinity (>50<100nM) and 90% of these are predicted in the ORF1ab (**Figure 9**).

**Table 3:** Strong (SB) and weak (WB) binders identified in the entire proteome (no spike).

	SB				WB			
PROTEIN	TOT	COMM	OMI	ALPHA	TOT	COMM	OMI	ALPHA
**ORF1ab**	101	99	1	1	61	60	0	1
**ORF3a**	7	7	0	0	3	3	0	0
**ENVELOPE**	2	2	0	0	4	4	0	0
**GLICOPROTEIN**	5	5	0	0	4	4	0	0
**ORF7a**	**3**	3	0	0	2	2	0	0
**ORF7b**	0	0	0	0	4	4	0	0
**ORF6**	1	1	0	0	2	2	0	0
**ORF8**	2	2	0	0	1	1	0	0
**NUCLEOCAPSIDE**	2	2	0	0	0	0	0	0
**ORF10**	1	1	0	0	0	0	0	0
	124	122	1	1	81	80	0	1

Epitopes were predicted from the entire proteome of the SARS-CoV-2 VOCs (no spike). The number of total SB and WB are reported, indicating the number of those common to all VOCs and of those VOC-specific.

**Figure 10:**
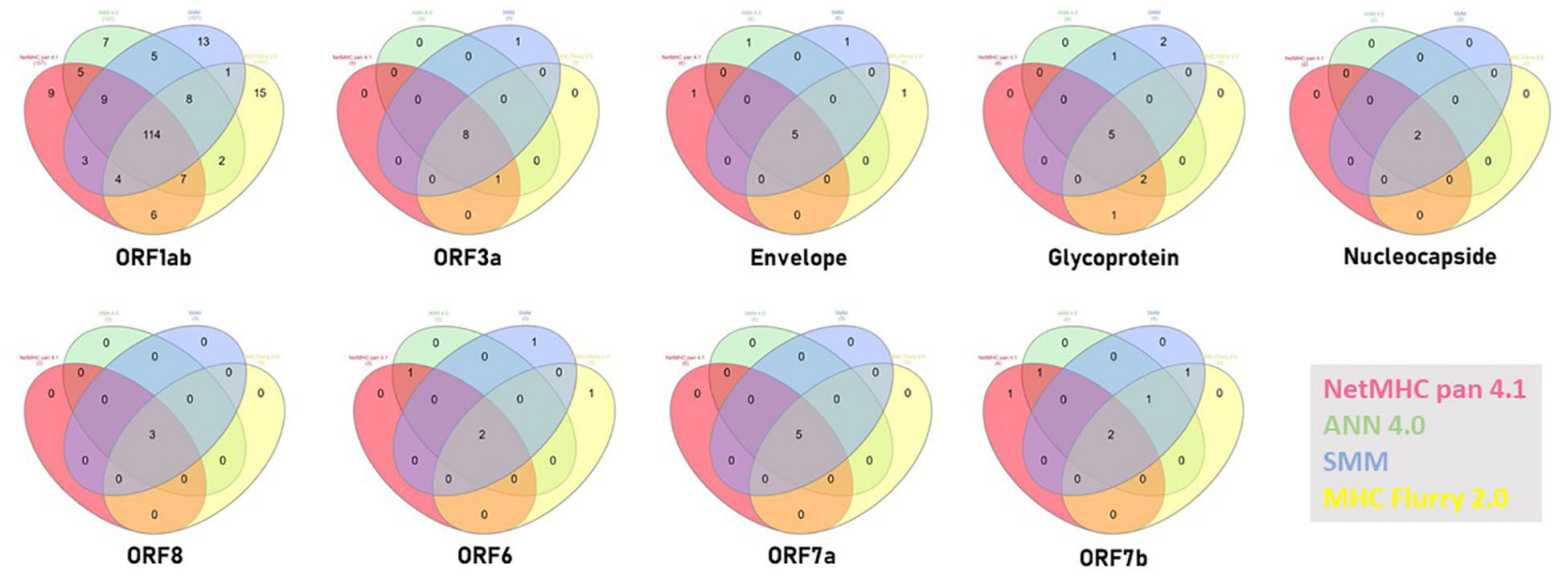
Compared prediction of SB+WB by all algorithms. The prediction of SB and WB non-spike epitopes predicted by NetMHC – Pan4.1 (<100nM) was assessed also with the indicated algorithms. Unique and shared predicted epitopes in the first ranking positions in all methods are indicated for all proteins.

In order to validate all the selected SB+WB epitopes in non-spike proteins, the same prediction analysis was performed using the additional three algorithms. The results showed that between 80 and 100% of the epitopes predicted by NetMHC-Pan4.1 were predicted also by all four algorithms (**Figure 10**; **Supplementary Table 10**).

### 3.9 Homology of SARS peptides to TAA epitopes

The SB and WB peptides predicted in the SARS-CoV-2 proteins, outside of the spike protein, were aligned to all published TAAs for sequence homology. The BLAST search returned significant homology with several known TAAs, mostly in the ORF 1ab, with not less than 4/9 identical aa residues. Twenty-six homologies were identified between SBs and TAAs, 21 of which with epitopes from the ORF1ab, and five homologies between WBs and TAAs, 3 of which with epitopes from the ORF1ab. Of interest, two of the ORF1ab-derived (LLLDDFVEI, VLLAPLLSA) and one of the Nucleocapside-derived (LLLDRLNQL) epitopes show sequence homology to two different TAAs. Several TAAs show homology with more than one viral epitope and, in particular, PRDX5 with five epitopes in both ORF1ab and Nucleocapside. As for the spike protein, HER-2 and GnTV TAAs were found to have homology also to ORF1ab and Membrane glycoprotein-derived epitopes (**Table 4**).

**Table 4:** Sequence homology between SARS-CoV-2 epitopes (no spike) and TAAs.

	Identity	Peptide	Aff(nM)	Binder	E-value	RMSD	Tumor
1	ORF 1AB	YLNTLTLAV	3.42	SB	0.87	0.1	MULTIPLE
	MDK	ALLALTSAV	9.8	SB			
2	ORF 1AB	LLLDDFVEI	4.5	SB	0.25	0.5	MULTIPLE
	KIF20A	LLSDDDVVV	32.87	SB			
3	ORF 1AB	LLLDDFVEI	4.5	SB	6.92x10^-4^	0.7	MELANOMA
	PRDX5	LLLDDLLVS	108.16	SB			
4	ORF 1AB	ALLADKFPV	4.7	SB	0.044	0.0	MULTIPLE
	MDK	ALLALTSAL	9.8	SB			
5	ORF 1AB	FVNEFYAYL	4.97	SB	0.005	0.2	LIVER CA
	GLYPICAN 3	FVGEFFTDV	19.75	SB			
6	ORF 1AB	FLPRVFSAV	5.52	SB	0.38	0.0	MELANOMA
	GnTV	VLPDVFIRV	7.59	SB			
7	ORF 1AB	ALWEIQQVV	5.73	SB	0.16	0.4	MULTIPLE
	IDO1	ALLEIASCL	26.78	SB			
8	ORF 1AB	FLNRFTTTL	6.61	SB	0.021	0.2	MULTIPLE
	CD274	LLNAFTVTV	4.62	SB			
9	ORF 1AB	SLPGVFCGV	7.48	SB	0.021	0.0	MELANOMA
	GnTV	VLPDVFIRV	9.08	SB			
10	ORF 1AB	YLNSTNVTI	12.5	SB	0.58	0.5	MULTIPLE
	CD274	LLNAFTVTV	4.62	SB			
11	ORF 1AB	SLLSVLLSM	17.23	SB	2.9	0.0	MELANOMA
	PRDX5	LLLDDLLVS	108.16	SB			
12	ORF 1AB	ILLLDQALV	17.57	SB	1.3	0.3	BREAST CA
	HER 2	RLLQETELV	30.47	SB			
13	ORF 1AB	KLVNKFLAL	25.98	SB	0.021	0.0	MULTIPLE
	Telomerase	RLVDDFLLV	5.33	SB			
14	ORF 1AB	TLNDLNETL	34.61	SB	0.01	0.3	MULTIPLE
	MELOE	TLNDECWPA	20.02	SB			
15	ORF 1AB	KLNEEIAII	34.66	SB	0.25	0.0	MELANOMA
	HAUS 3	ILNAMIAKI	42.38	SB			
16	ORF 1AB	VLLAPLLSA	46.66	SB	0.067	0.3	MULTIPLE
	Nectin-4	VLVPPLPSL	30.78	SB			
17	ORF 1AB	VLLAPLLSA	46.66	SB	1.9	0.3	MELANOMA
	PRDX5	LLLDDLLVS	108.16	SB			
18	ORF 1AB	SLLMPILTL	47.58	SB	0.03	0.6	MULTIPLE
	MUC 1	LLLLTVLTV	20.01	SB			
19	ORF 1AB	ALLSDLQDL	48.1	SB	0.067	0.7	MELANOMA
	PRDX5	LLLDDLLVS	108.16	SB			
20	ORF 1AB	KLVSSFLEM	58.34	SB	0.25	0.0	MULTIPLE
	Telomerase	RLVDDFLLV	5.33	SB			
21	ORF 1AB	NVLTLVYKV	73.8	SB	4.3	0.6	MULTIPLE
	MAGE-A1	KVLWYVIKV	5.38	SB			
22	ORF 3A	ALLAVFHSA	21.8	SB	0.044	0.0	MULTIPLE
	MDK	ALLALTSAV	9.8	SB			
23	GLYCOPR	KLLEEWNLV	6.49	SB	0.25	0.3	BREAST CA
	HER-2	RLLQETELV	30.47	SB			
24	GLYCOPR	KLLEQWNLV	7.24	SB	4.3	0.3	BREAST CA
	HER-2	RLLQETELV	30.47	SB			
25	NUCLEOC	LLLDRLNQL	10.55	SB	0.25	0.2	MULTIPLE
	BING-4	CQWGRLWQL	24.33	SB			
26	NUCLEOC	LLLDRLNQL	10.55	SB	0.014	0.3	MELANOMA
	PRDX5	LLLDDLLVS	108.16	SB			
27	ORF 1AB	LLFLMSFTV	9.96	WB	0.87	0.7	MULTIPLE
	MUC 1	LLLLTVLTV	20.01	SB			
28	ORF 1AB	GVFCGVDAV	44.69	WB	0.11	0	PROSTATE
	Hepsin	GLQLGVQAV	77.31	SB			
29	ORF 1AB	VLLSMQGAV	86.01	WB	0.38	0	MULTIPLE
	MDK	ALLALTSAV	9.8	SB			
30	GLYCOPR	TLACFVLAA	25.06	WB	0.38	0.7	COLON CA
	HEPACAM	RLAPFVYLL	3.57	SB			
31	ORF 7	FLALITLAT	65.85	WB	0.014	0	THYROID
	CALCA	FLALSILVL	30.04	SB			

The epitopes have been aligned to homologous TAAs, as identified via BLAST search. Aligned aminoacid sequences are shown for each epitope pair. Green color indicates identity between the residues in the paired epitopes. The affinity for each peptide is reported with the tumor expressing the TAA.

**Figure 11:**
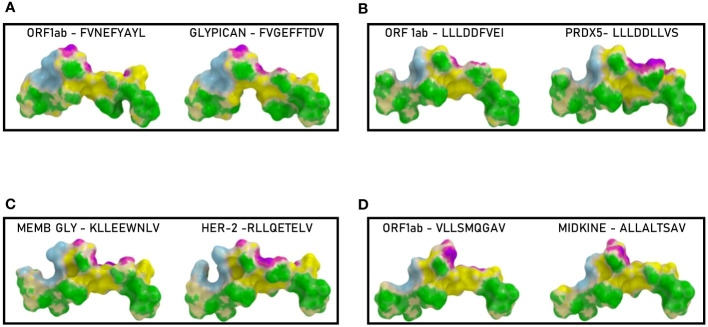
Predicted 3D conformation of non-SPIKE peptides and paired TAAs. The conformation of the SARS-CoV-2 derived epitopes (ORF and MEMB) and paired TAAs bound to the indicated HLA-A*02:01 molecule is shown. The paired epitopes are indicated in each box **(A–D)**. The prediction was performed using as template the publicly available crystallized structure (PDB https://www.rcsb.org/structure/1AO7). Green areas = contact points with HLA molecule; light Blue areas = contact points with the TCR α chain; Violet areas = contact points with the TCR β chain.

Binding to HLA-A*02:01 was experimentally confirmed using the TAP-deficient T2 cells (**Supplementary Figure 13**). The comparison analysis of the non-spike peptides with the homologous TAAs showed high similar conformation and contact areas to the HLA molecule as well as to α and β chains of the TCR (**Figures 11A-D**). On the contrary, all other peptides showed poor conformation similarity to TAAs (**Supplementary Figures 14-19**).

Such observations were confirmed by the analysis of the paired peptides positioned in the HLA peptide-binding groove, in particular looking at the footprint of the paired peptides on the HLA groove (**Figure 12**; **Supplementary Figures 20-31**).

**Figure 12:**
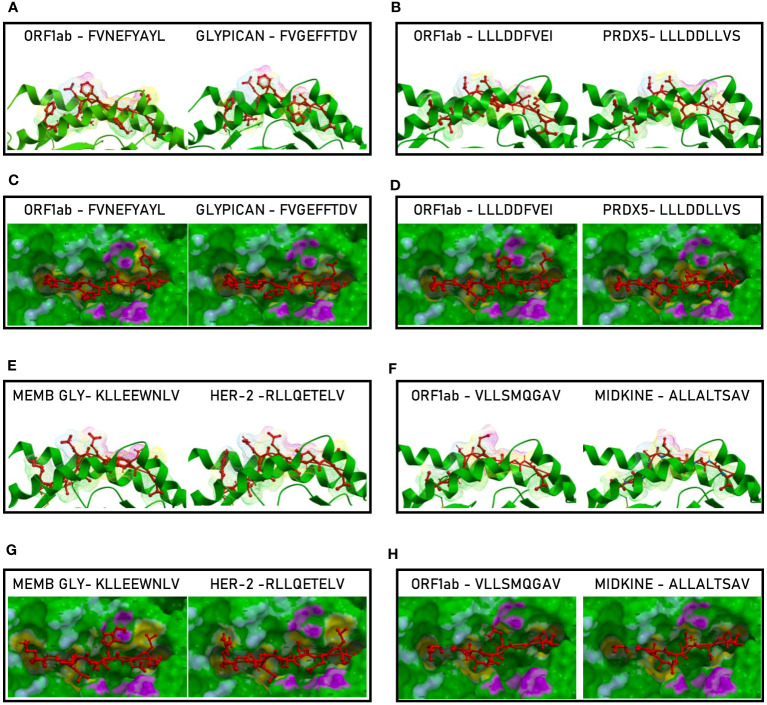
Molecular mimicry of non-spike epitopes and TAAs: interaction with the HLA molecule. The paired epitopes are shown bound to the HLA-A*02:01 molecule. The TCR facing residues are presented to the TCR αβ chains with the same conformation **(A, B, E, F)**. The footprint of the paired epitopes on the HLA molecule is shown and the contact points are highlighted in yellow **(C, D, G, H)**.

**Figure 13:**
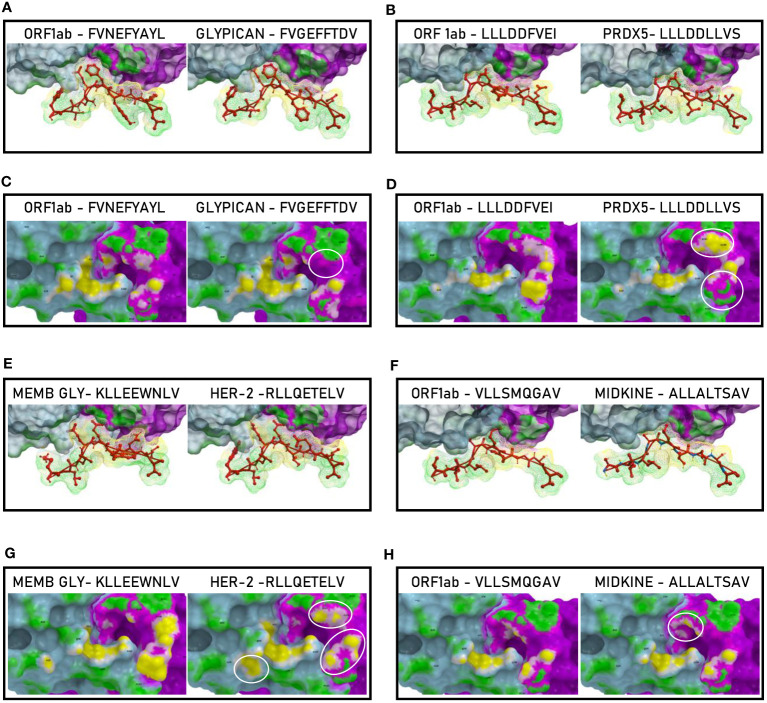
Molecular mimicry of non-spike epitopes and TAAs: interaction with the TCR molecule. The paired peptides are shown in contact with the TCR αβ chains **(A, B, E, F).** The footprint of the paired epitopes on the TCR αβ chains is shown and the contact points are highlighted in yellow (α chain) and in pink β (chain). For each pair, differences in the contact areas are circled **(C, D, G, H)**.

The analyses of the interaction between the paired peptides and the α and β chains of the TCR provided a further perspective. As reasonably predictable, none of the paired peptides show identical footprints on the TCR chains (**Figure 13**). However, the ORF1ab and the paired Glypican and MDK TAAs, show minor differences only in the contact areas with the G_100_ and G_101_ residues of the β chain (**Figures 13, C, H**). On the contrary, the ORF1ab and the Memb Glyc show more pronounced differences with the paired PRDX5 and Her-2 TAAs in the contact areas with the L_98_, G_100_ and G_101_ residues of the β chain (**Figures 13D, G**). No relevant differences in the contact areas with the α chain of the TCR were observed for the pairs (**Figure 11H**). Overall, more pronounced differences in the contact areas with both α and β chains of the TCR were observed in other epitope pairs (**Supplementary Figures 32–43**).

### 3.10 T cell cross-reactivity to non-spike epitopes and TAAs

**Figure 14:**
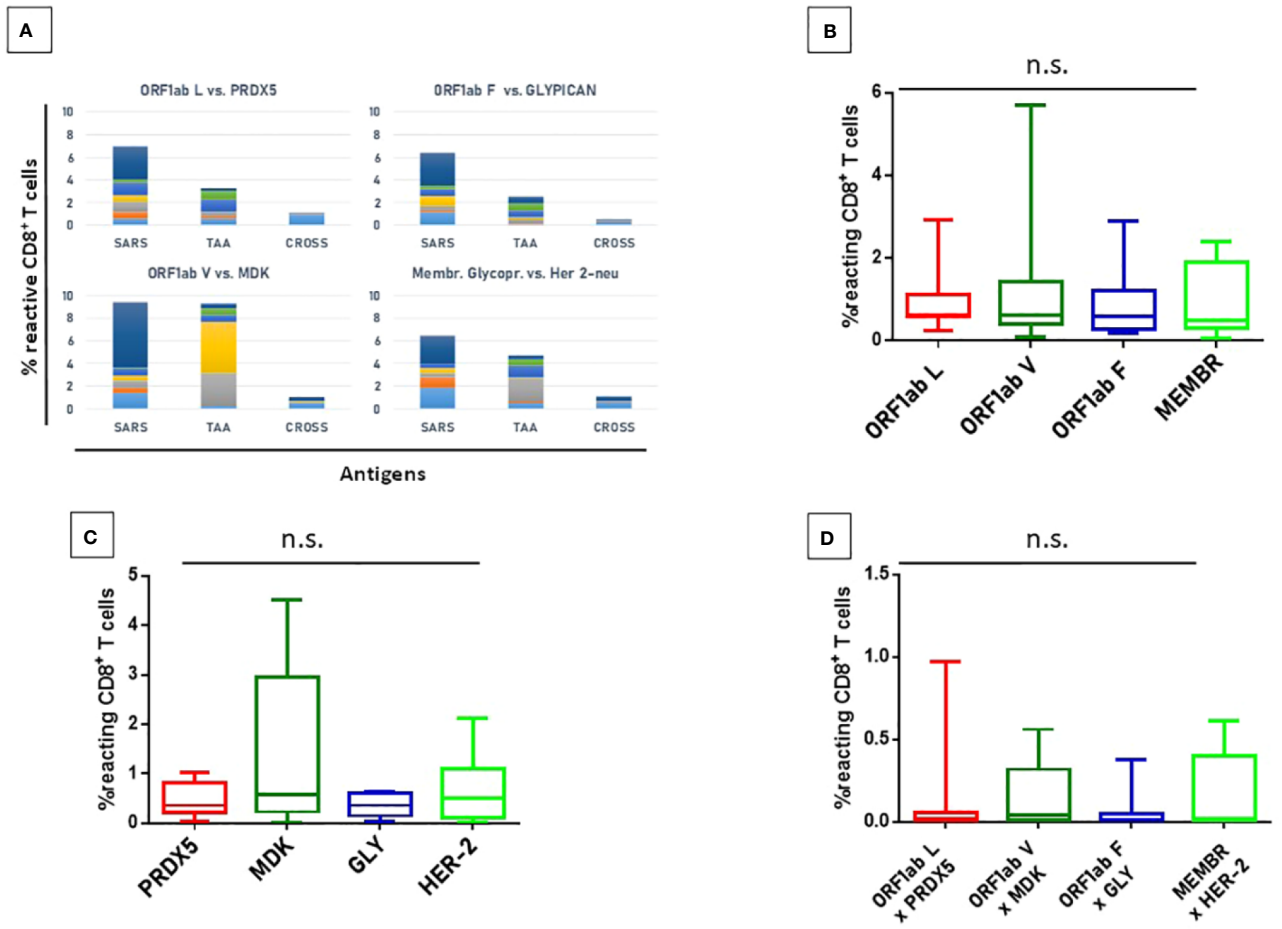
T cell reactivity to non-spike epitopes and paired TAAs. PBMCs from HLA-A*02:01 positive vaccinated were analyzed by tetramer-staining with the indicated epitopes **(A)**. Results of reactivity to individual SARS epitopes **(B)**, paired TAA **(C)** and cross-reactivity **(D)** are shown. ORF1ab L = LLLDDFVEI; ORF1ab V = VLLSMQGAV; ORF1ab F = FVNEFYAYL. n.s. means not statistical significant.

In order to evaluate if the paired non-Spike and TAAs peptides with high sequence and conformational homologies were recognized by cross-reacting CD8^+^ T cells, an MHC I Dextramer complex-staining was performed in the same experimental setting described for the spike-derived peptides. Four paired epitopes were selected according to the conformational homology and the range of predicted affinity (9.8 – 108.16nM). The results showed a substantial percentage of circulating reacting CD8^+^ T cells specific to the SRAS-CoV-2 peptides, with average values not statistically different (0,99 - 1.34%). However, significant variability was observed among the tested

subjects and, in particular, one showed high percentage of reacting CD8^+^ T cells against all the SARS-CoV-2-derived epitopes (2.4 – 5.7%) (**Figure 14**).

A lower but significant percentage of circulating reacting CD8^+^ T cells specific to the homologous TAAs epitopes, with an average included between 0.36% (Glypican) and 1.34 (MDK) but the differences between the groups do not reach the statistical difference (**Figure 9**). Finally, a low level of CD8^+^ T cell cross-reactivity was observed for the four paired peptides, with average values of 0.06 – 0.16%, showing a direct correlation with the levels of reactivity against the individual peptides (**Figure 14**). *Reactivities against the negative control (namely a scrambled peptide) was between 0.0% and 0.065% in all tested subjects (0.034% on average; ± 0.004 standard deviation). For all subjects the response to each SARS and TAA was statistically significant compared to the natural background (p <0.05).*

### 3.11 Total T cell cross-reactivity to SARS-CoV-2 epitopes and TAAs

In order to evaluate the cumulative effect induced by the epitopes derived from the entire SARS-CoV-2 proteome, we analyzed the total CD8^+^ T cell responses against the viral epitopes, their paired TAAs and the cross-reactivities.

**Figure 15:**
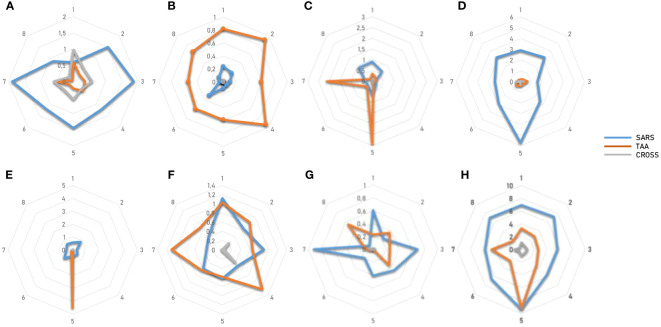
T cell reactivity to non-spike epitopes and paired TAAs. PBMCs from HLA-A*02:01 positive vaccinated were analyzed by tetramer-staining with the indicated epitopes. Results of reactivity in each subject to individual SARS epitopes, paired TAA and cross-reactivity is shown **(A-G)**. The sum of the reactivity in all subjects to individual SARS epitopes, paired TAA and cross-reactivity is shown **(H)**.

The overall analysis confirmed that the percentage of reacting CD8^+^ T cells to each individual epitope (SARS and TAA) is very variable from subject to subject, with a wide range from <1% to 6%. Some of the subjects show a consistent reactivity to all SARS or TAA epitopes, others a more focused one. Interestingly, selective high reactivity to TAAs, MDK the highest, is observed (p<0.001). Overall, considering the sum of the reactivities of all subjects, the highest percentage is observed for the VLLSMQGAV_ORF1ab_/ALLALTSAV_MDK_ and KLLEEWNLV_MEMBR/_RLLQETELV_HER-2_ epitope pairs, reaching >9% and ~6% of the total circulating T cells, respectively (**Figure 15**; **Supplementary Figure 44**).

## 4 Discussion

In the present study we screened all the possible MHC-I T cell epitopes in the entire SARS-CoV-2 proteome predicted to bind the HLA-A*02:01 allele and their homology to tumor associated antigens (TAAs). The ultimate goal is to assess whether the T cell response elicited by the vaccination and/or the productive infection with the SARS-CoV-2 may represent a natural priming to cancer antigens, to be promptly recalled and boosted in case of tumor growth.

The analysis was first focused on epitopes derived from the Spike protein, to assess the role of the vaccination. Most of them are shared among all the VOCs, with only few identified in variants different from the Wuhan, suggesting that all the vaccines implemented around the World (based on the Wuhan isolate) have potentially elicited the broadest spectrum of T cells response. Regardless all the predicted strong and weak binding epitopes (18 SB and 39 WB) in the spike protein, we confirmed, as previously reported by us and others, that the most immunogenic epitope in the contest of the HLA-A*02:01 allele is the YLQPRTFLL. The BLAST search did not return any homology between this peptide and known TAAs, implying that the strong anti-SARS-CoV-2 T cell response elicited by the vaccination is unlike to represent an immunological priming, potentially protective against tumor growth. Considering the remaining predicted 56 SB and WB peptides in the spike protein, 10 partial homologies with TAAs were found, equally distributed in the SP1 and SP2 subunits. Four of these homologies were associated to the Beta (one) and Omicron (three) VOCs only, suggesting that the infection with such VOCs may elicit a broader priming against tumor antigens. Obviously, this broadening effect does not apply to vaccinated individuals, which have been immunized with the Wuhan variant only. Such homology between the paired epitopes has been further corroborated by molecular docking analysis, which showed a very high similarity in the conformation of the peptides and the pattern of contact with the HLA molecule as well as α and β chains of the TCR. The reactivity of T cells from healthy subjects to TAAs and, yet more, the cross-reactivity of T cells to both epitopes provided the ultimate confirmation of the molecular mimicry between the viral and tumor antigens. The CDR3 regions of the TCR α and β chains binding the HLMSFPQSA_SP2.4_ and the paired LLWSFQTSA_TYR_ peptides have been identified and are publicly available. There is no shared sequence among the CDR3 binding the two epitopes, although the consensus shows high conservation at the NH_2_- and COOH- termini. Nevertheless, considering the profusely reported broad variability in the CDR3 sequences binding a very same epitope, the finding in the present study cannot exclude that some of these CDR3 are indeed able to bind both epitopes. The same pipeline of analysis has been applied to all other SARS-CoV-2 proteins. Indeed, also vaccinated subjects may be infected by the replicating virus and therefore primed by any peptide derived from the viral proteome. The total number of predicted peptides is 205 (124 SB and 81 WB); 81.5% of the SBs and 75.3% of the WBs are predicted in the ORF1ab, which, indeed, represents 73% of the entire viral proteome. The homology with TAAs was identified for 26 SBs and 5 WBs from the SARS epitopes and 25/31 of them (80.1%) were from the ORF1ab. Two of the ORF1ab-derived (LLLDDFVEI, VLLAPLLSA) and one of the Nucleocapside-derived (LLLDRLNQL) epitopes show sequence homology to two different TAAs. Likewise, some of the TAAs show homology to more SARS epitopes, namely the LLLDDLLVS_PRDX5_ (5 homologies) and the ALLALTSAV_MDK_ (4 homologies).

Also for these paired epitopes, the sequence homology has been further corroborated by molecular docking analysis, which showed a very high similarity in the conformation of the peptides and the pattern of contact with the HLA molecule as well as α and β chains of the TCR. As predictable, all the paired SARS-CoV-2 peptides (spike and non-spike) and TAAs show high E-values confirming that they do not have any evolutionary relationships. Indeed, they are the result of a random event with a likely relevance in protection from cancer development, progression and final clinical outcome. Moreover, the RMSD values are all <1 which are considered as proof of highly similar structure. The top paired peptides were experimentally confirmed to bind the HLA-A*02:01 using the TAP-deficient T2 cells.

Moreover, the reactivity of T cells from healthy subjects to SARS epitopes, TAAs and, yet more, the cross-reactivity of T cells to both epitopes provided the ultimate confirmation of the molecular mimicry between these viral and tumor antigens. As predictable, the reactivity of cells from each subject to each epitope is significantly variable with completely different patterns, indicating a highly individual responsiveness. Nevertheless, the cumulative analysis show a consistent reactivity to all SARS and TAA epitopes with an average of 6.77% and 3.81% of reacting CD8^+^ T cells, respectively. Furthermore, the percentage of cross-reactive T cells is, on average, 0.81%. In particular, the highest percentage is observed for the VLLSMQGAV_ORF1ab_/ALLALTSAV_MDK_ and KLLEEWNLV_MEMBR/_RLLQETELV_HER-2_ epitope pairs, reaching >9% and ~6% of the total circulating T cells, respectively. For these pairs, the cross-reactive CD8^+^ T cells reach 1% of the total circulating T cells, confirming that the higher is the reactivity for the individual peptides the higher is the cross-reactivity.

Overall, in accordance to the proposed mechanism of the molecular mimicry in cancer patients infected by SARS-CoV-2 (38), the findings of the present study describe for the first time that significant homology is found between several epitopes derived from the entire SARS-CoV-2 proteome and the whole list of known TAAs. The present study is limited to the HLA-A*02:01 haplotype, which is present in about 40–50% of the Italian and Western general population and, therefore, it has a great impact. A much larger observation group will allow to generalize this result to many other haplotypes.

The level of sequence and conformational homology, together with the percentages of CD8^+^ T cells reacting with the individual epitopes and/or cross-reacting with both of them, has a high potential implication. Indeed, this suggests that the T cell response elicited during the natural infection by one or multiple SARS-CoV-2 derived epitopes may prime the immune system against several TAAs. The predicted epitopes showing a molecular mimicry with TAAs are mostly (>95%) common to all VOCs of the SARS-CoV-2, implying that the individual variants provides a very limited increased protective effect.

Therefore, the SARS-CoV-2 infection may represent a “natural anti-cancer vaccination” eliciting a memory T cell compartment able to cross-react with cancer cells and providing a protection from cancer development and progression. Such a protecting effect will be evaluated in the coming years by epidemiological studies assessing the incidence of several tumor types, including breast, liver, melanoma and colon cancers. Consequently, the viral antigens homologous to TAAs may be used for developing “multi-cancer” off-the-shelf preventive/therapeutic vaccine formulations, with higher antigenicity and immunogenicity than over-expressed tumor self-antigens, for the potential valuable benefit of thousands of cancer patients around the World.

## Data availability statement

The raw data are available at https://zenodo.org/records/11232764.

## Ethics statement

The studies involving humans were approved by Istituto Nazionale Tumori - IRCCS PASCALE, Naples IT. The studies were conducted in accordance with the local legislation and institutional requirements. The participants provided their written informed consent to participate in this study.

## Author contributions

CR: Writing – original draft, Formal analysis, Methodology. AM: Formal analysis, Methodology, Writing – original draft. BCa: Methodology, Writing – review & editing. EC: Writing – review & editing, Resources. LR: Resources, Writing – review & editing. CM: Writing – review & editing, Methodology. SM: Methodology, Writing – review & editing. BCe: Methodology, Writing – review & editing. MT: Writing – review & editing, Supervision. LB: Supervision, Writing – review & editing, Conceptualization, Funding acquisition, Writing – original draft.

## Supplementary material

Supplementary Figure 1 IFNγ EliSpot assay. Interferon gamma production by PBMCs from each individual subject activated by the spike epitopes HLA-A*02:01 YLQPRTFLL_NTD_ and KIADYNYKL_RBD_. The negative control is the epitope HLA-A*01:01 LTDEMIAQY_NTD_. SFU = Spot Forming Units.

Supplementary Figure 2 Experimental binding of TAAs and spike viral paired peptides to HLA-A*0201. Binding to HLA-A*0201 molecule was assessed in TAP-deficient T2 cells loaded with the indicated peptides. **(A)** Mean fluorescence intensity at flow cytometer indicates binding levels of peptides to HLA molecules. **(B)**.

Supplementary Figure 3,4 Predicted 3D conformation of SARS-CoV-2 pike peptides and paired TAAs. The conformation of the SARS-CoV-2 no-spike derived epitopes and paired TAAs bound to the indicated HLA-A*02:01 molecule is shown. The prediction was performed using as template the publicly available crystallized structure (PDB https://www.rcsb.org/structure/1AO7). Green areas = contact points with HLA molecule; light Blue areas = contact points with the TCR α chain; Violet areas = contact points with the TCR β chain

Supplementary Figure 5-8 Molecular mimicry of SARS-CoV-2 spike epitopes and TAAs: interaction with the HLA molecule. The paired epitopes are shown bound to the HLA-A*02:01 molecule. The TCR facing residues are presented to the TCR αβ chains with the same conformation **(A, B, E, F)**. The footprint of the paired epitopes on the HLA molecule is shown and the contact points are highlighted in yellow **(C, D, G, H)**.

Supplementary Figure 9,12 Molecular mimicry of SARS-CoV-2 spike epitopes and TAAs: interaction with the TCR molecule. The paired peptides are shown in contact with the TCR αβ chains **(A, B, E, F)**. The footprint of the paired epitopes on the TCR αβ chains is shown and the contact points are highlighted in yellow (α chain) and in pink β (chain). For each pair, differences in the contact areas are circled **(C, D, G, H)**.

Supplementary Figure 13 Experimental binding of TAAs and no-spike viral paired peptides to HLA-A*0201. Binding to HLA-A*0201 molecule was assessed in TAP-deficient T2 cells loaded with the indicated peptides. A. Mean fluorescence intensity at flow cytometer indicates binding levels of peptides to HLA molecules. B.

Supplementary Figure 14-19 Predicted 3D conformation of SARS-CoV-2 no-spike peptides and paired TAAs. The conformation of the SARS-CoV-2 no-spike derived epitopes and paired TAAs bound to the indicated HLA-A*02:01 molecule is shown. The prediction was performed using as template the publicly available crystallized structure (PDB https://www.rcsb.org/structure/1AO7). Green areas = contact points with HLA molecule; light Blue areas = contact points with the TCR α chain; Violet areas = contact points with the TCR β chain

Supplementary Figure 20-31 Molecular mimicry of SARS-CoV-2 no-spike epitopes and TAAs: interaction with the HLA molecule. The paired epitopes are shown bound to the HLA-A*02:01 molecule. The TCR facing residues are presented to the TCR αβ chains with the same conformation **(A, B, E, F)**. The footprint of the paired epitopes on the HLA molecule is shown and the contact points are highlighted in yellow **(C, D, G, H)**.

Supplementary Figure 32-43 Molecular mimicry of SARS-CoV-2 no-spike epitopes and TAAs: interaction with the TCR molecule. The paired peptides are shown in contact with the TCR αβ chains **(A, B, E, F)**. The footprint of the paired epitopes on the TCR αβ chains is shown and the contact points are highlighted in yellow (α chain) and in pink β (chain). For each pair, differences in the contact areas are circled **(C, D, G, H)**.

Supplementary Figure 44 Cross-reactive CD8+ T cells in pMHC tetramer staining. Unstimulated PBMCs from sample a representative subject were incubated with pMHC tetramers loaded with the indicated TAA and MoAs homologous peptides. The percentage of reactive CD8^+^ T cells to the individual epitopes as well as the cross-reactive ones are shown.

## References

[1] Lurie N Keusch GT Dzau VJ. Urgent lessons from COVID 19: why the world needs a standing, coordinated system and sustainable financing for global research and development. Lancet. (2021) 397(10280):1229–36. doi: 10.1016/S0140-6736(21)00503-1 PMC7993931 33711296

[2] Gao FX Wu RX Shen MY Huang JJ Li TT Hu C. Extended SARS-CoV-2 RBD booster vaccination induces humoral and cellular immune tolerance in mice. iScience. (2022) 25(12):1054–79. doi: 10.1016/j.isci.2022.105479 PMC9625849 36338436

[3] McGill COVID19 Vaccine Tracker Team. Pfizer/BioNTech: comirnaty. Available online at: https://covid19.trackvaccines.org/vaccines/6/.

[4] Filia A Urdiales AM Fadda G. Comirnaty (BNT162b2), Il primo vaccino contro il COVID-19 approvato in Europa e in Italia: Istituto Superiore di Sanità –Dipartimento malattie infettive; Rome, Italy. (2021).

[5] Walls AC Park YJ Tortorici MA Wall A McGuire AT Veesler D. Structure, function, and antigenicity of the SARS-CoV-2 spike glycoprotein. Cell. (2020) 181(2):281–8292.e6. doi: 10.1016/j.cell.2020.02.058 PMC7102599 32155444

[6] Yan R Zhang Y Li Y Xia L Guo Y Zhou Q. Structural basis for the recognition of SARS-CoV-2 by full-length human ACE2. Science (New York, N.Y.). (2020) 367:1444–8. doi: 10.1126/science.abb2762 PMC7164635 32132184

[7] McCallum M De Marco A Lempp FA Tortorici MA Pinto D Walls AC. N-terminal domain antigenic mapping reveals a site of vulnerability for SARS-CoV-2. Cell. (2021) 184(9):2332–2347.e16. doi: 10.1016/j.cell.2021.03.028 PMC7962585 33761326

[8] Polack FP Thomas SJ Kitchin N Absalon J Gurtman A Lockhart S. Safety and efficacy of the BNT162b2 mRNA Covid-19 vaccine. N Engl J Med. (2020) 383(27):2603–15. doi: 10.1056/NEJMoa2034577 PMC7745181 33301246

[9] Thomas SJ Moreira ED Jr Kitchin N Absalon J Gurtman A Lockhart S. Safety and efficacy of the bnt162b2 mrna covid-19 vaccine through 6 months. N Engl J Med. (2021) 385(19):1761–73. doi: 10.1056/NEJMoa2110345 PMC8461570 34525277

[10] Moreira ED Jr Kitchin N Xu X Dychter SS Lockhart S Gurtman A. Safety and efficacy of a third dose of BNT162b2 Covid-19 vaccine. N Engl J Med. (2022) 386(20):1910–21. doi: 10.1056/NEJMoa2200674 PMC9006787 35320659

[11] Cavalcanti E Isgrò MA Rea D Di Capua L Trillò G Russo L. Vaccination strategy and anti - SARS-CoV-2 S titers in healthcare workers of the INT – IRCCS “Fondazione pascale” Cancer center (Naples, Italy). Inf Agents Cancer. (2021) 16(1):32. doi: 10.1186/s13027-021-00375-2 PMC8114024 33980271

[12] Ragone C Meola S Fiorillo PC Penta R Auriemma L Tornesello ML. HLA does not impact on short-medium-term antibody response to preventive anti-SARS-Cov-2 vaccine. Front Immunol. (2021) 12:734689. doi: 10.3389/fimmu.2021.734689 34386018 PMC8353253

[13] Sahin U Muik A Vogler I Derhovanessian E Kranz LM Vormehr M. BNT162b2 vaccine induces neutralizing antibodies and poly-specific T cells in humans. Nature. (2021) 595(7868):572–7. doi: 10.1038/s41586-021-03653-6 34044428

[14] Grifoni A Weiskopf D Ramirez SI Mateus J Dan JM Moderbacher CR. Targets of T cell responses to SARS-CoV-2 coronavirus in humans with COVID-19 disease and unexposed individuals. Cell. (2020) 181(7):1489–1501.e15. doi: 10.1016/j.cell.2020.05.015 PMC7237901 32473127

[15] Weiskopf D Schmitz KS Raadsen MP Grifoni A Okba NMA Endeman H. Phenotype and kinetics of SARS-CoV-2-specific T cells in COVID-19 patients with acute respiratory distress syndrome. Sci Immunol. (2020) 5(48):eabd2071. doi: 10.1126/sciimmunol.abd2071 32591408 PMC7319493

[16] Braun J Loyal L Frentsch M Wendisch D Georg P Kurth F. SARS-CoV-2-reactive T cells in healthy donors and patients with COVID-19. Nature. (2020) 587(7833):270–4. doi: 10.1038/s41586-020-2598-9 32726801

[17] Gangaev A Ketelaars SLC Isaeva OI Patiwael S Dopler A Hoefakker K. Identification and characterization of a SARS-CoV-2 specific CD8+ T cell response with immunodominant features. Nat Commun. (2021) 12(1):2593. doi: 10.1038/s41467-021-22811-y 33972535 PMC8110804

[18] Le Bert N Tan AT Kunasegaran K Tham CYL Hafezi M Chia A. SARS-CoV-2-specific T cell immunity in cases of COVID-19 and SARS, and uninfected controls. Nature. (2020) 584(7821):457–62. doi: 10.1038/s41586-020-2550-z 32668444

[19] Ferretti AP Kula T Wang Y Nguyen DMV Weinheimer A Dunlap GS. Unbiased screens show CD8+ T cells of COVID-19 patients recognize shared epitopes in SARS-CoV-2 that largely reside outside the spike protein. Immunity. (2020) 53(5):1095–1107.e3. doi: 10.1016/j.immuni.2020.10.006 PMC7574860 33128877

[20] Hernandez SPA Hersby DS Munk KK Tamhane T Trubach D Tagliamonte M. Three doses of BNT162b2 COVID-19 mRNA vaccine establish long-lasting CD8+ T cell immunity in CLL and MDS patients. Front Immunol. (2023) 13:1035344. doi: 10.3389/fimmu.2022.1035344 36703960 PMC9873231

[21] Wooldridge L Ekeruche-Makinde J van den Berg HA Skowera A Miles JJ Tan MP. A single autoimmune T cell receptor recognizes more than a million different peptides. J Biol Chem. (2012) 287(2):1168–77. doi: 10.1074/jbc.M111.289488 PMC3256900 22102287

[22] Sewell AK. Why must T cells be cross-reactive? Nat Rev Immunol. (2012) 12(9):669–77. doi: 10.1038/nri3279 PMC7097784 22918468

[23] Loftus DJ Castelli C Clay TM Squarcina P Marincola FM Nishimura M. Identification of epitope mimics recognized by CTL reactive to the melanoma/melanocyte-derived peptide MART-1(27–35). J Exp Med. (1996) 184(2):647–57. doi: 10.1084/jem.184.2.647 PMC2192745 8760818

[24] Pittet MJ Valmori D Dunbar PR Speiser DE Liénard D Lejeune F. High frequencies of naive Melan-A/MART-1-specific CD8(+) T cells in a large proportion of human histocompatibility leukocyte antigen (HLA)-A2 individuals. J Exp Med. (1999) 190(5):705–15. doi: 10.1084/jem.190.5.705 PMC2195613 10477554

[25] Dutoit V Rubio-Godoy V Pittet MJ Zippelius A Dietrich PY Legal FA. Degeneracy of antigen recognition as the molecular basis for the high frequency of naive A2/Melan-a peptide multimer(+) CD8(+) T cells in humans. J Exp Med. (2002) 196(2):207–16. doi: 10.1084/jem.20020242 PMC2193921 12119345

[26] Rubio-Godoy V Dutoit V Zhao Y Simon R Guillaume P Houghten R. Positional scanning-synthetic peptide library-based analysis of self- and pathogen-derived peptide cross-reactivity with tumor-reactive Melan-A-specific CTL. J Immunol (Baltimore, Md. : 1950). (2002) 169(10):5696–707. doi: 10.4049/jimmunol.169.10.5696 12421949

[27] Petrizzo A Tagliamonte M Mauriello A Costa V Aprile M Esposito R. Unique true predicted neoantigens (TPNAs) correlates with anti-tumor immune control in HCC patients. J Transl Med. (2018) 16(1):286. doi: 10.1186/s12967-018-1662-9 30340600 PMC6194606

[28] Cavalluzzo B Mauriello A Ragone C Manolio C Tornesello ML Buonaguro FM. Novel molecular targets for hepatocellular carcinoma. Cancers. (2021) 14(1):140. doi: 10.3390/cancers14010140 35008303 PMC8750630

[29] Chiou SH Tseng D Reuben A Mallajosyula V Molina IS Conley S. Global analysis of shared T cell specificities in human non-small cell lung cancer enables HLA inference and antigen discovery. Immunity. (2021) 54(3):586–602.e8. doi: 10.1016/j.immuni.2021.02.014 PMC7960510 33691136

[30] Ragone C Manolio C Cavalluzzo B Mauriello A Tornesello ML Buonaguro FM. Identification and validation of viral antigens sharing sequence and structural homology with tumor-associated antigens (TAAs). J Immunother Cancer. (2021) 9(5):e002694. doi: 10.1136/jitc-2021-002694 34049932 PMC8166618

[31] Ragone C Manolio C Mauriello A Cavalluzzo B Buonaguro FM Tornesello ML. Molecular mimicry between tumor associated antigens and microbiota-derived epitopes. J Transl Med. (2022) 20(1):316. doi: 10.1186/s12967-022-03512-6 35836198 PMC9281086

[32] Tagliamonte M Buonaguro L. The impact of antigenic molecular mimicry on anti-cancer T-cell immune response. Front Oncol. (2022) 12:1009247. doi: 10.3389/fonc.2022.1009247 PMC9623278 36330482

[33] Manolio C Ragone C Cavalluzzo B Mauriello A Tornesello ML Buonaguro FM. Antigenic molecular mimicry in viral-mediated protection from cancer: the HIV case. J Trans Med. (2022) 20(1):472. doi: 10.1186/s12967-022-03681-4 PMC9569184 36243758

[34] Tagliamonte M Cavalluzzo B Mauriello A Ragone C Buonaguro FM Tornesello ML. Molecular mimicry and cancer vaccine development. Mol Cancer. (2023) 22(1):75. doi: 10.1186/s12943-023-01776-0 37101139 PMC10131527

[35] Buonaguro L Cavalluzzo B Mauriello A Ragone C Tornesello AL Buonaguro FM. Microorganisms-derived antigens for preventive anti-cancer vaccines. Mol Aspects Med. (2023) 92:101192. doi: 10.1016/j.mam.2023.101192 37295175

[36] Saini SK Tamhane T Anjanappa R Saikia A Ramskov S Donia M. Empty peptide-receptive MHC class I molecules for efficient detection of antigen-specific T cells. Sci Immunol. (2019) 4(37):eaau9039. doi: 10.1126/sciimmunol.aau9039 31324690

[37] Francis JM Leistritz-Edwards D Dunn A Tarr C Lehman J Dempsey C. Allelic variation in class I HLA determines CD8+ T cell repertoire shape and cross-reactive memory responses to SARS-CoV-2. Sci Immunol. (2022) 7(67):eabk3070. doi: 10.1126/sciimmunol.abk3070 34793243 PMC9017864

[38] Burgio S Conway de Macario E Macario AJ Cappello F. SARS-CoV-2 in patients with cancer: possible role of mimicry of human molecules by viral proteins and the resulting anti-cancer immunity. Cell Stress Chaperones. (2021) 26(4):611–6. doi: 10.1007/s12192-021-01211-7 PMC8112475 33977496

