# Supplementary material for: Molecular mimicry of SARS-COV-2 antigens as a possible natural anti-cancer preventive immunization
Source: Front Immunol. 2024 Jun 14;15:1398002. doi: 10.3389/fimmu.2024.1398002 (PMC11211543; doi:10.3389/fimmu.2024.1398002)
Supplement: Supplementary Figure 1 — IFNγ EliSpot assay. Interferon gamma production by PBMCs from each individual subject activated by the spike epitopes HLA-A*02:01 YLQPRTFLLNTD and KIADYNYKLRBD. The negative control is the epitope HLA-A*01:01 LTDEMIAQYNTD. SFU = Spot Forming Units. [file DataSheet_1.pdf]

Suppl. Fig. 1

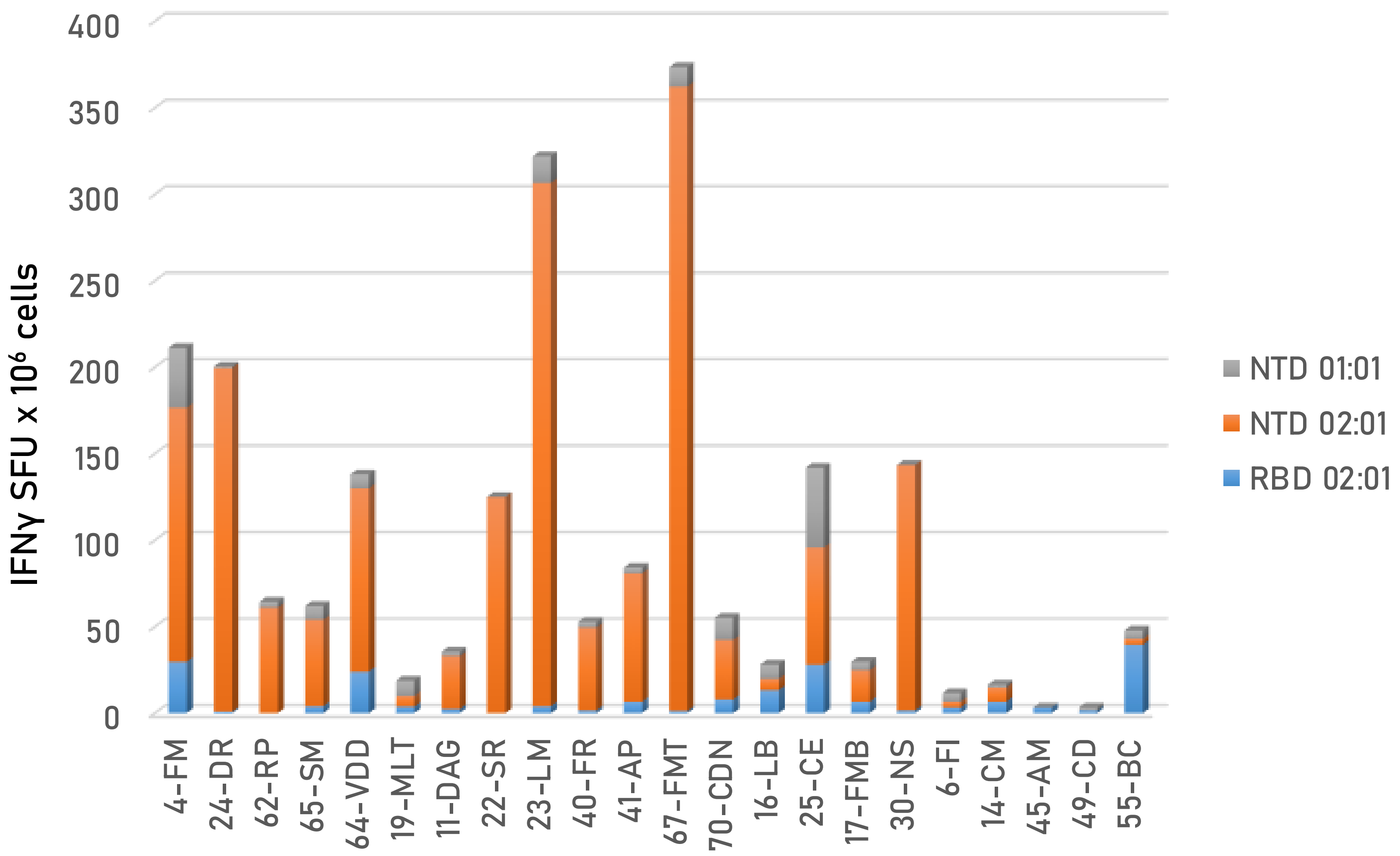

RBD 02:01 = KIADYNYKL
NTD 02:01 = YLQPRTFLL
NTD 01:01 = LTDEMIAQY

Suppl. Fig. 2

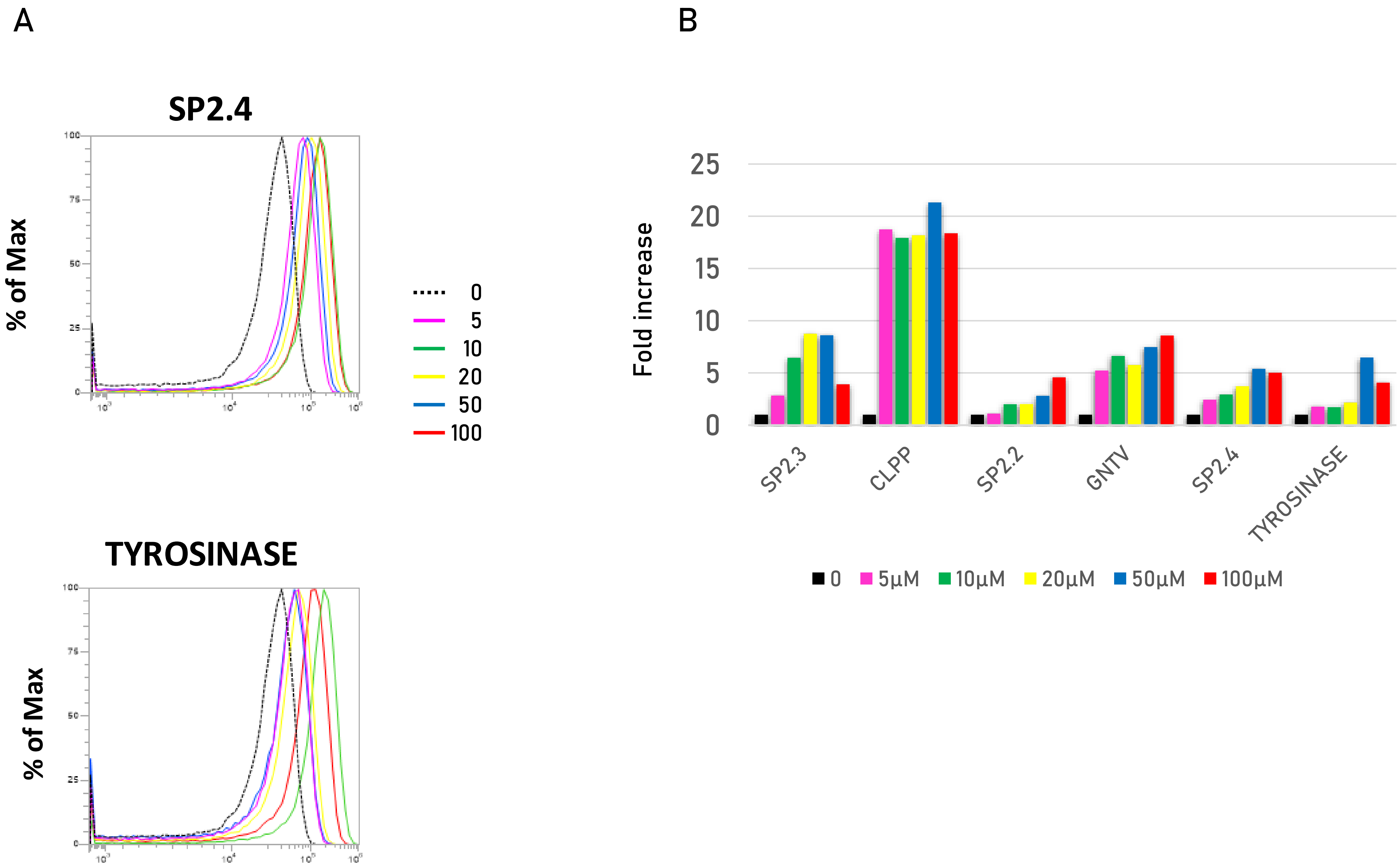
A
B
SP2.4
% of Max
0
5
10
20
50
100
TYROSINASE
% of Max
Fold increase
25
20
15
10
5
0
SP2.3
CLPP
SP2.2
GNTV
SP2.4
TYROSINASE
0
5µM
10µM
20µM
50µM
100µM

Suppl. Fig. 3

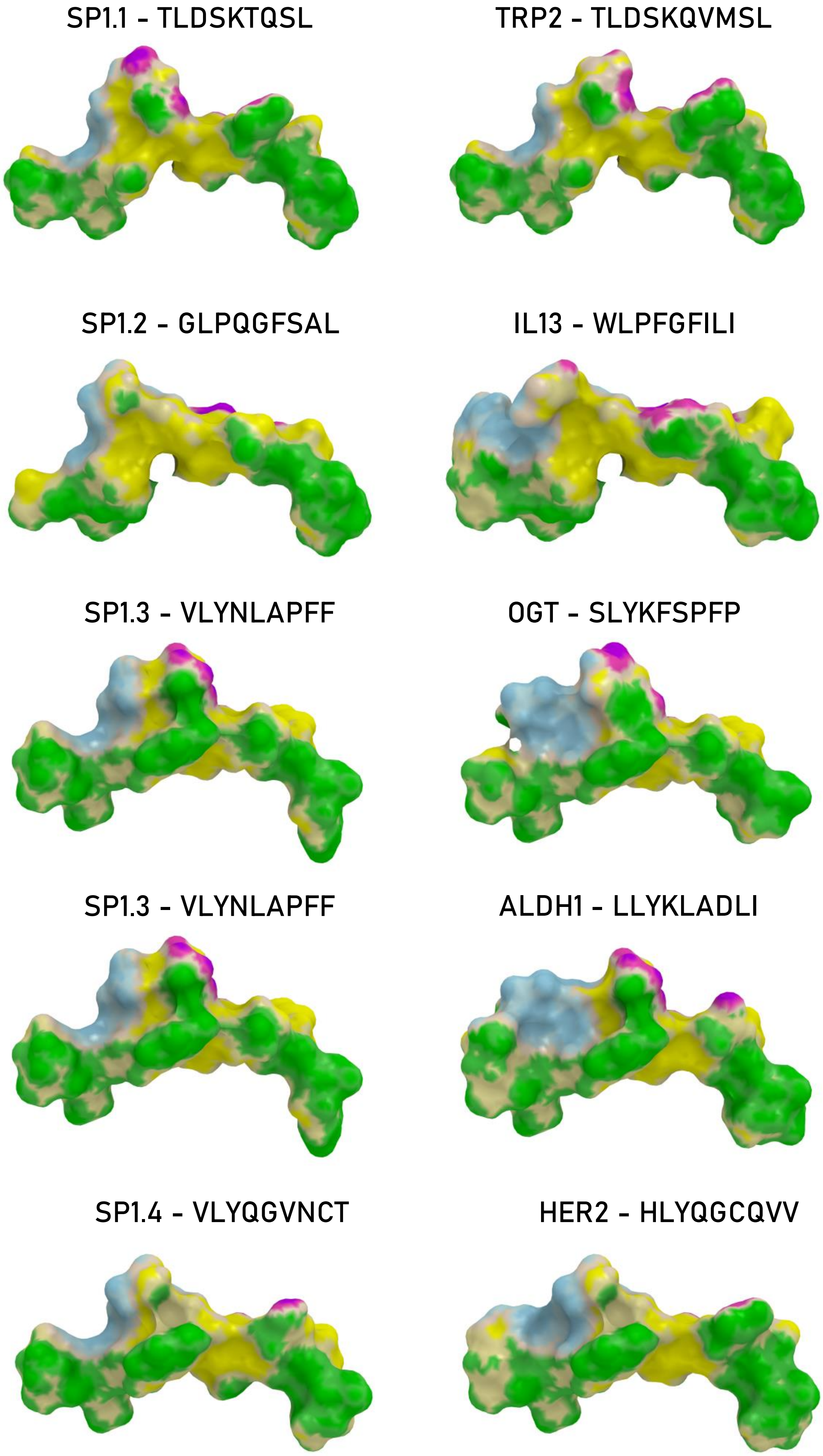
SP1.1 - TLDSKTQSL
TRP2 - TLDSKQVMSL
SP1.2 - GLPQGFSAL
IL13 - WLPFGFILI
SP1.3 - VLYNLAPFF
OGT - SLYKFSPFP
SP1.3 - VLYNLAPFF
ALDH1 - LLYKLADLI
SP1.4 - VLYQGVNCT
HER2 - HLYQGCQVV

Suppl. Fig. 4

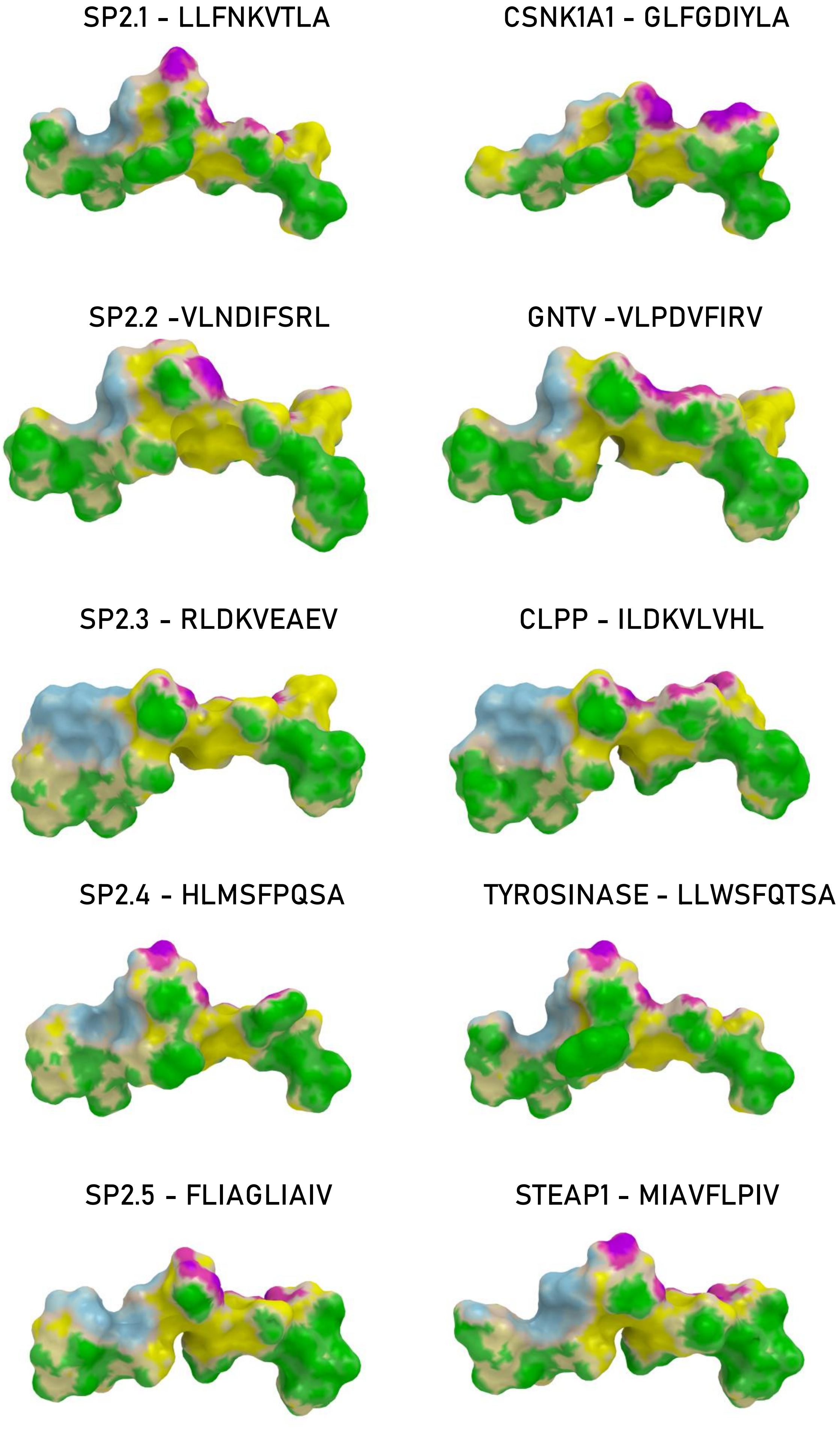
SP2.1 - LLFNKVTLA
CSNK1A1 - GLFGDIYLA
SP2.2 -VLNDIFSRL
GNTV -VLPDVFIRV
SP2.3 - RLDKVEAEV
CLPP - ILDKVLVHL
SP2.4 - HLMSFPQSA
TYROSINASE - LLWSFQTSA
SP2.5 - FLIAGLIAIV
STEAP1 - MIAVFLPIV

Suppl. Fig. 5

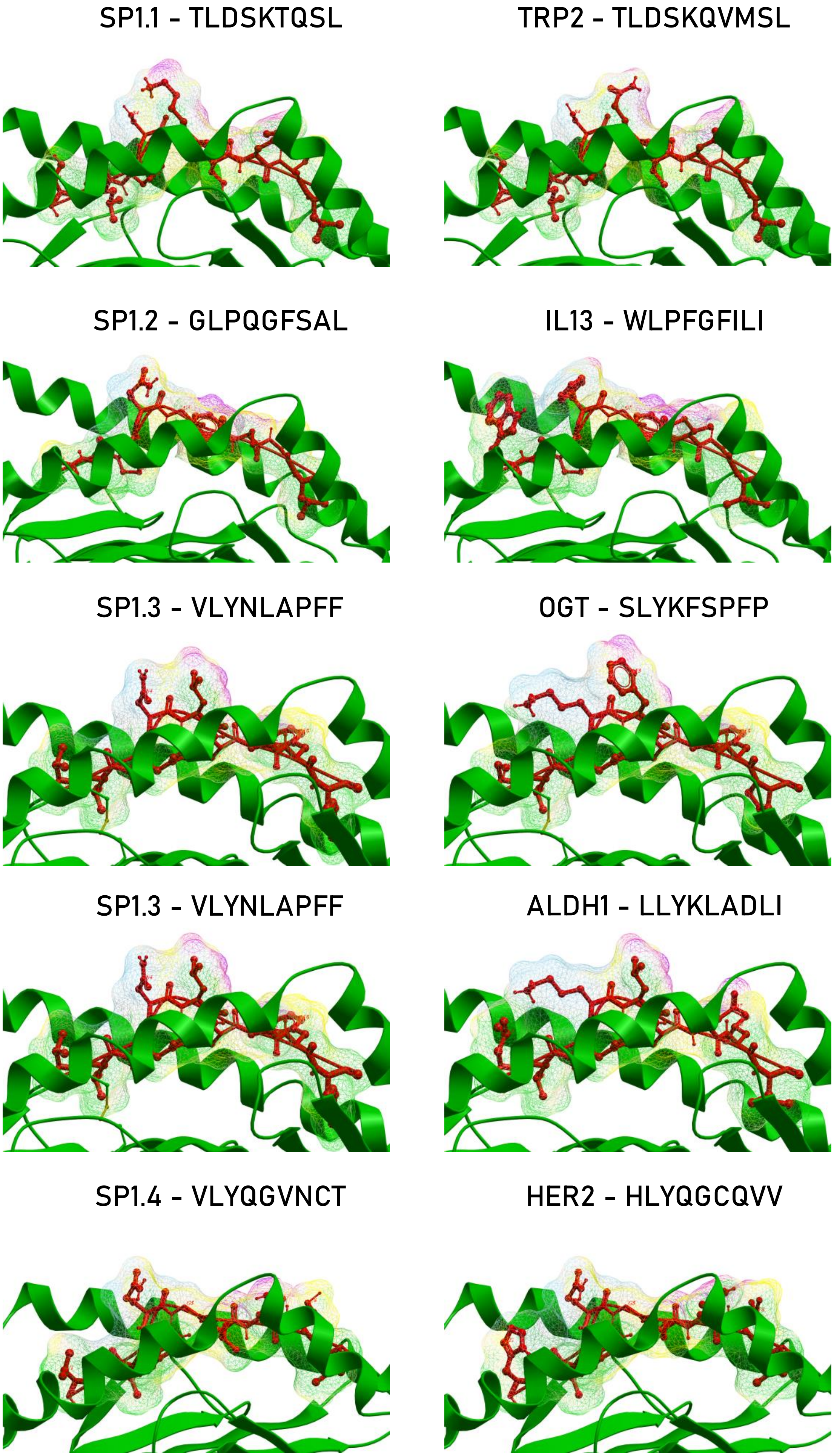
SP1.1 - TLDSKTQSL
TRP2 - TLDSKQVMSL
SP1.2 - GLPQGFSAL
IL13 - WLPFGFILI
SP1.3 - VLYNLAPFF
OGT - SLYKFSPFP
SP1.3 - VLYNLAPFF
ALDH1 - LLYKLADLI
SP1.4 - VLYQGVNCT
HER2 - HLYQGCQVV

Suppl. Fig. 6

SP1.1 - TLDSKTQSL

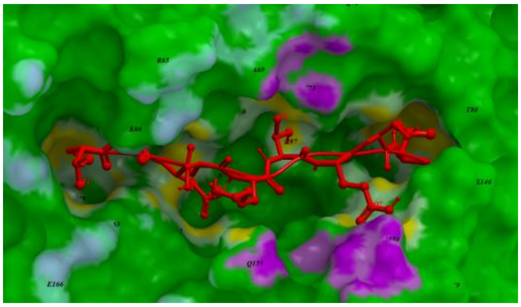

TRP2 - TLDSKQVMSL

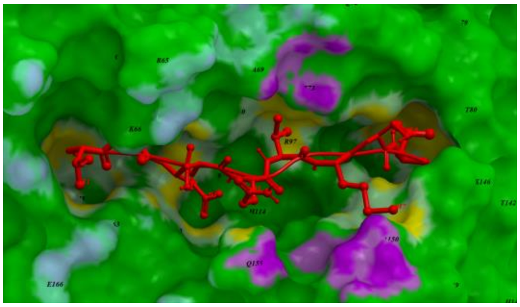

SP1.2 - GLPQGFSAL

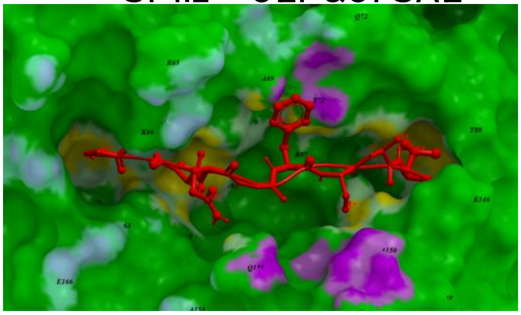

IL13 - WLPFGFILI

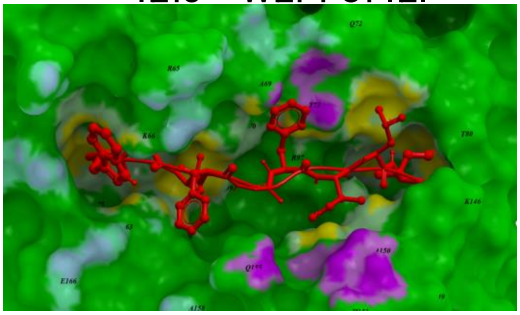

SP1.3 - VLYNLAPFF

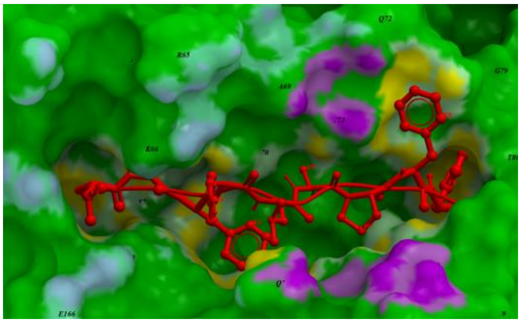

OGT - SLYKFSPFP

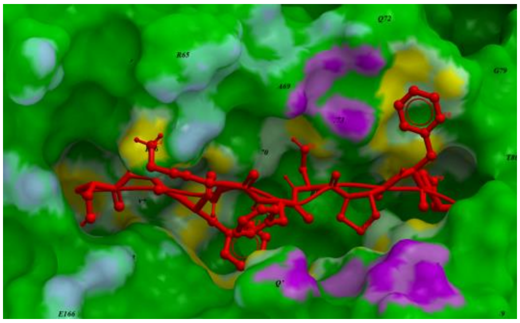

SP1.3 - VLYNLAPFF

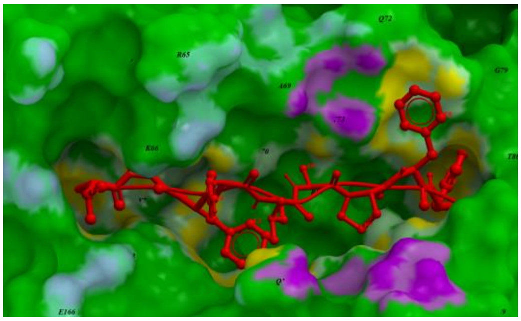

ALDH1 - LLYKLADLI

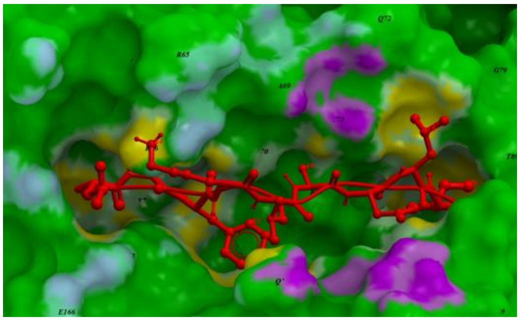

SP1.4 - VLYQGVNCT

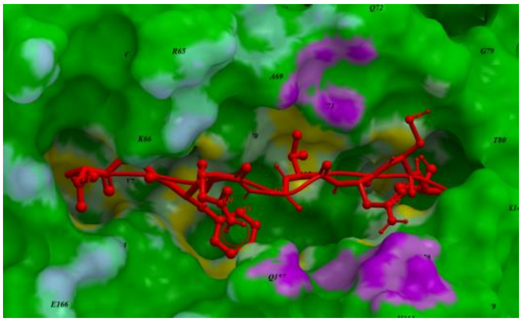

HER2 - HLYQGCQVV

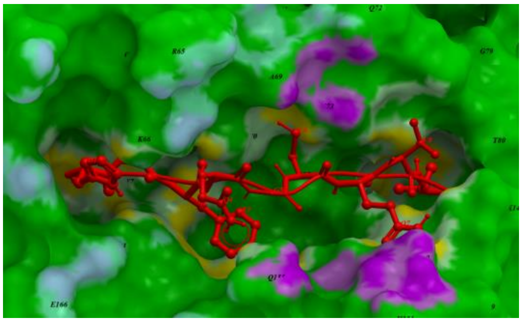

Suppl. Fig. 7

SP2.1 - LLFNKVTLA

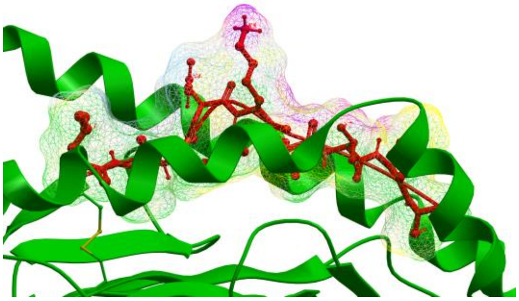

CSNK1A1 - GLFGDIYLA

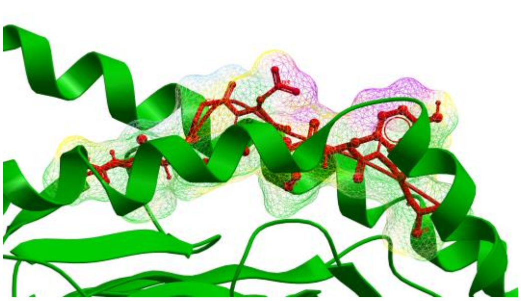

SP2.2 -VLNDIFSRL

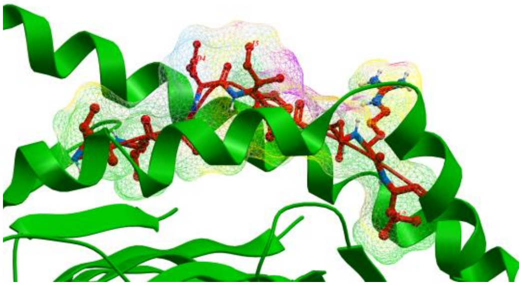

GNTV -VLPDVFIRV

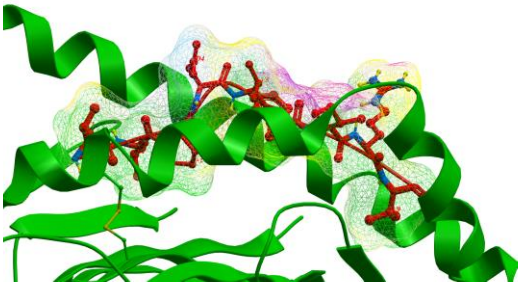

SP2.3 - RLDKVEAEV

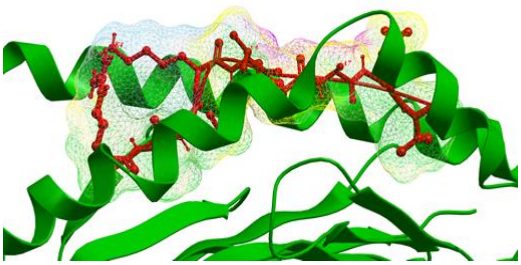

CLPP - ILDKVLVHL

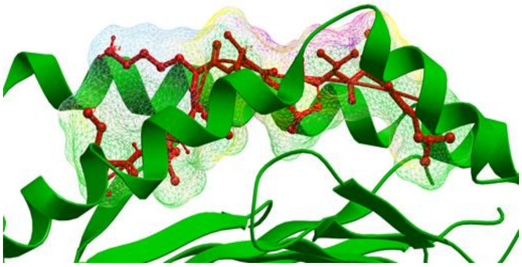

SP2.4 - HLMSFPQSA

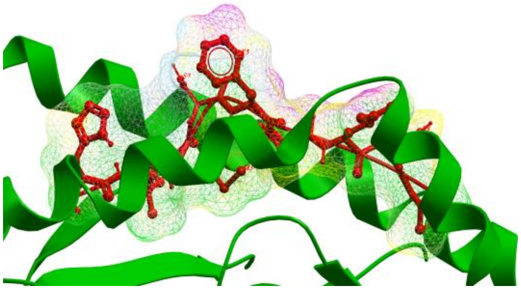

TYROSINASE - LLWSFQTSA

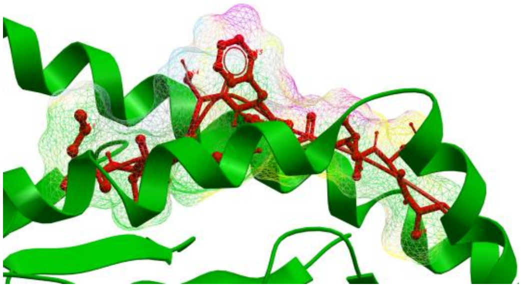

SP2.5 - FLIAGLIAIV

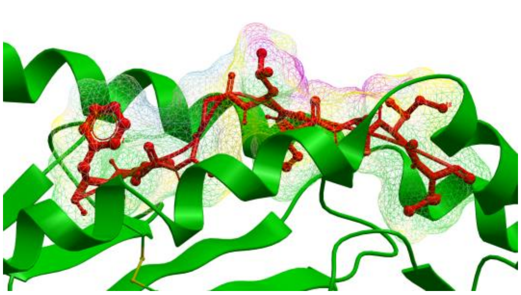

STEAP1 - MIAVFLPIV

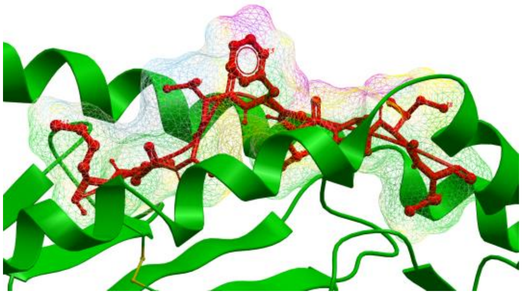

Suppl. Fig. 8

SP2.1 - LLFNKVTLA

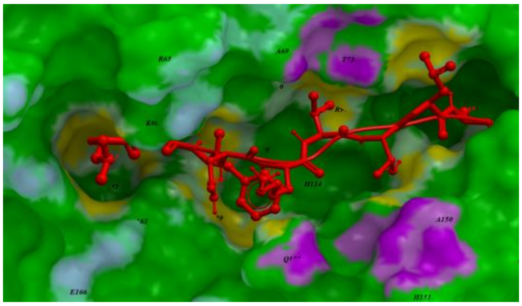

CSNK1A1 - GLFGDIYLA

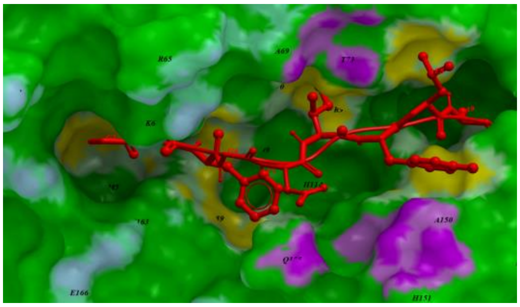

SP2.2 -VLNDIFSRL

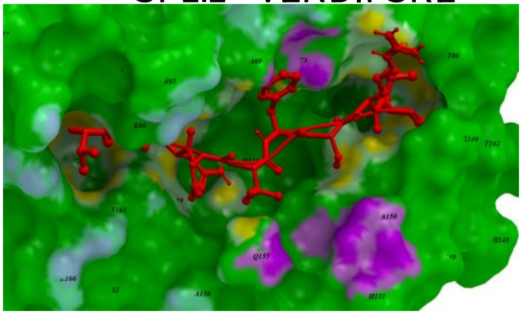

GNTV -VLPDVFIRV

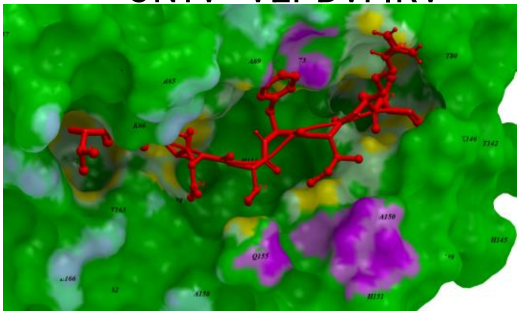

SP2.3 - RLDKVEAEV

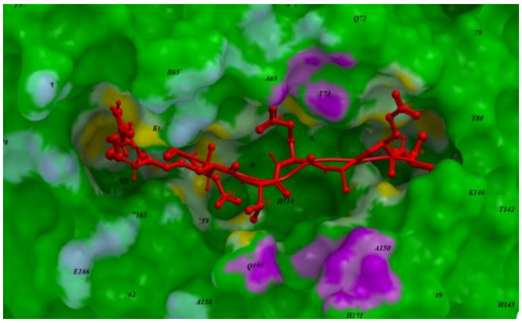

CLPP - ILDKVLVHL

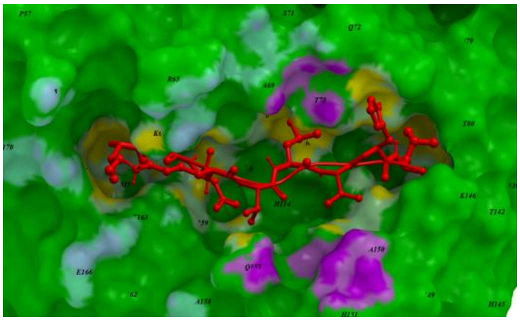

SP2.4 - HLMSFPQSA

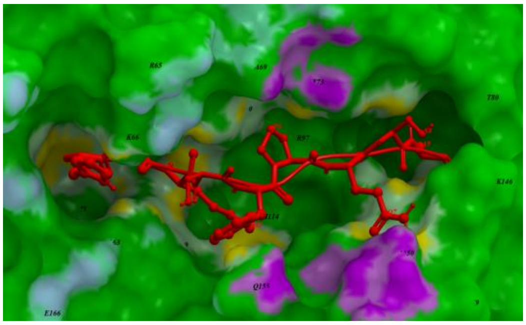

TYROSINASE - LLWSFQTSA

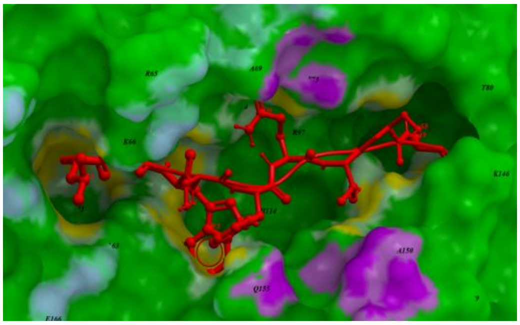

SP2.5 - FLIAGLIAIV

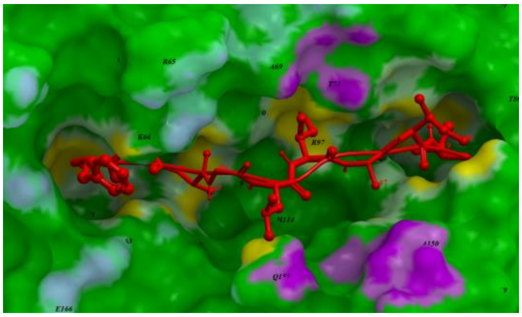

STEAP1 - MIAVFLPIV

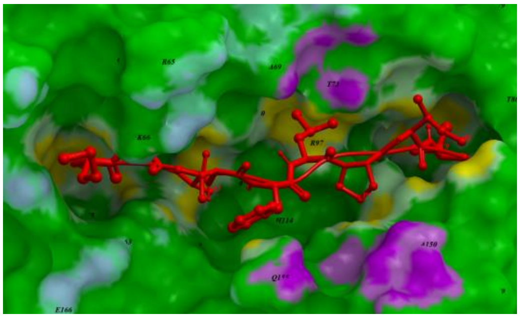

Suppl. Fig. 9

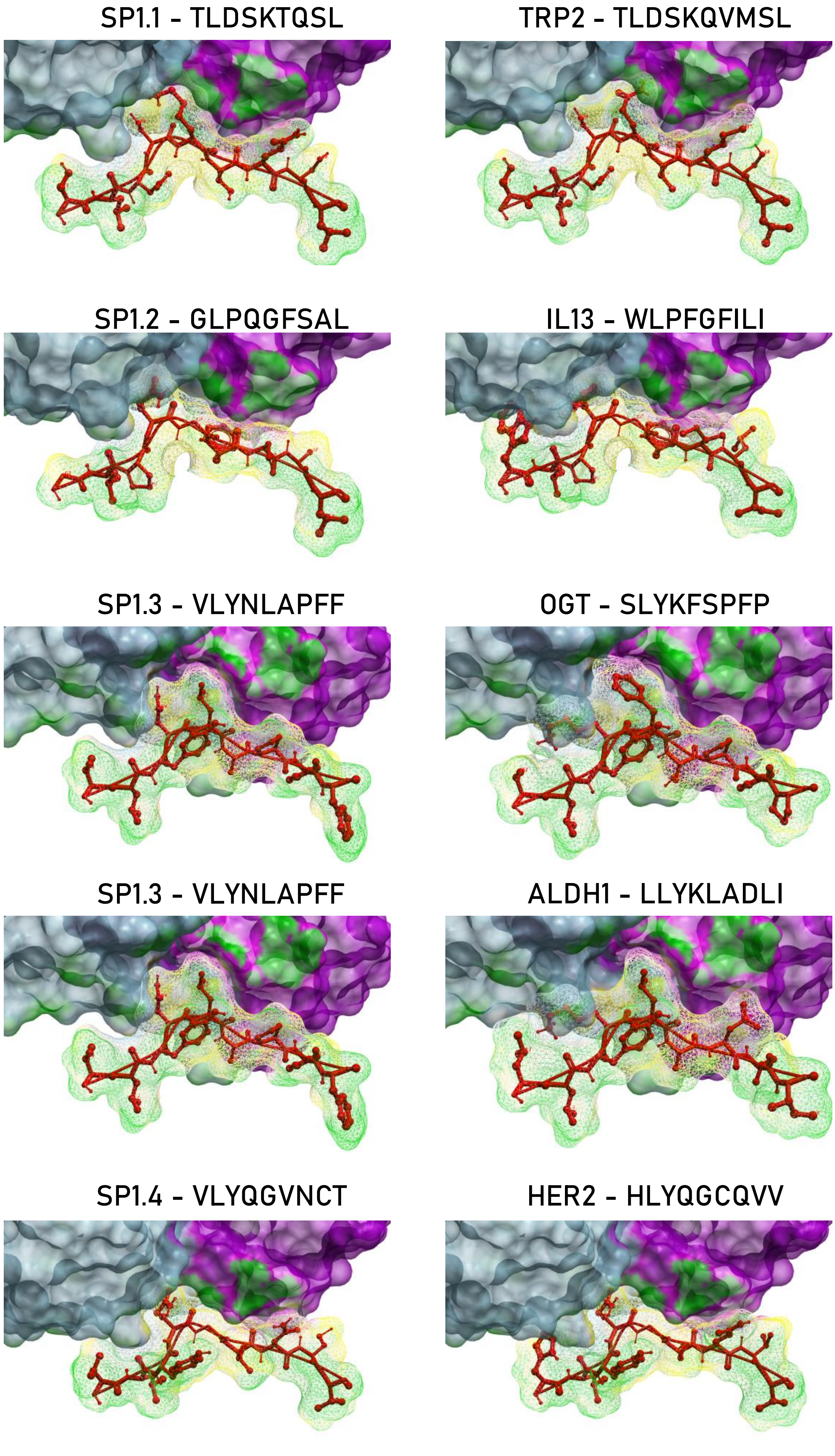
SP1.1 - TLDSKTQSL
TRP2 - TLDSKQVMSL
SP1.2 - GLPQGFSAL
IL13 - WLPFGFILI
SP1.3 - VLYNLAPFF
OGT - SLYKFSPFP
SP1.3 - VLYNLAPFF
ALDH1 - LLYKLADLI
SP1.4 - VLYQGVNCT
HER2 - HLYQGCQVV

Suppl. Fig. 10

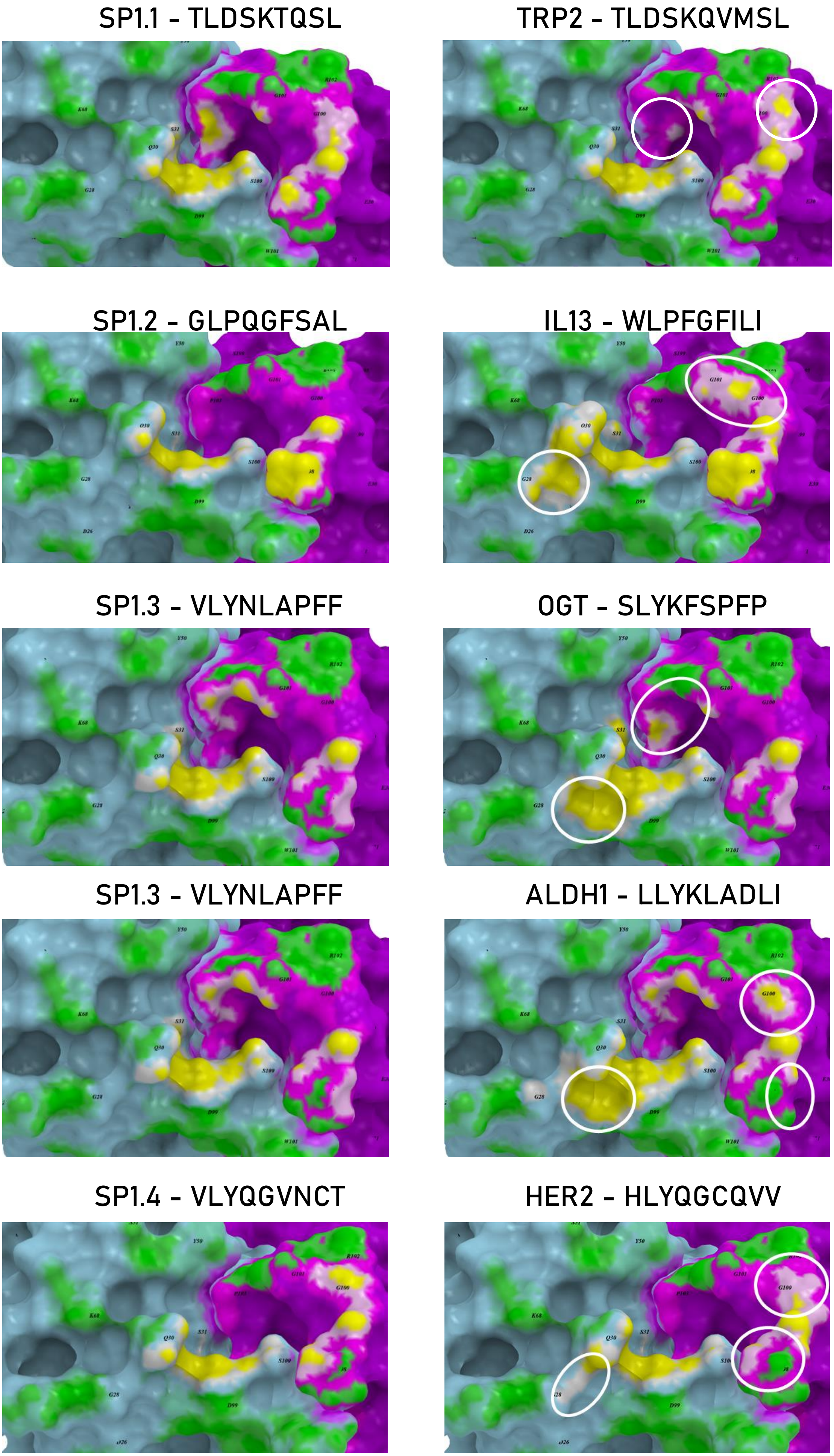
SP1.1 - TLDSKTQSL
TRP2 - TLDSKQVMSL
SP1.2 - GLPQGFSAL
IL13 - WLPFGFILI
SP1.3 - VLYNLAPFF
OGT - SLYKFSPFP
SP1.3 - VLYNLAPFF
ALDH1 - LLYKLADLI
SP1.4 - VLYQGVNCT
HER2 - HLYQGCQVV

Suppl. Fig. 11

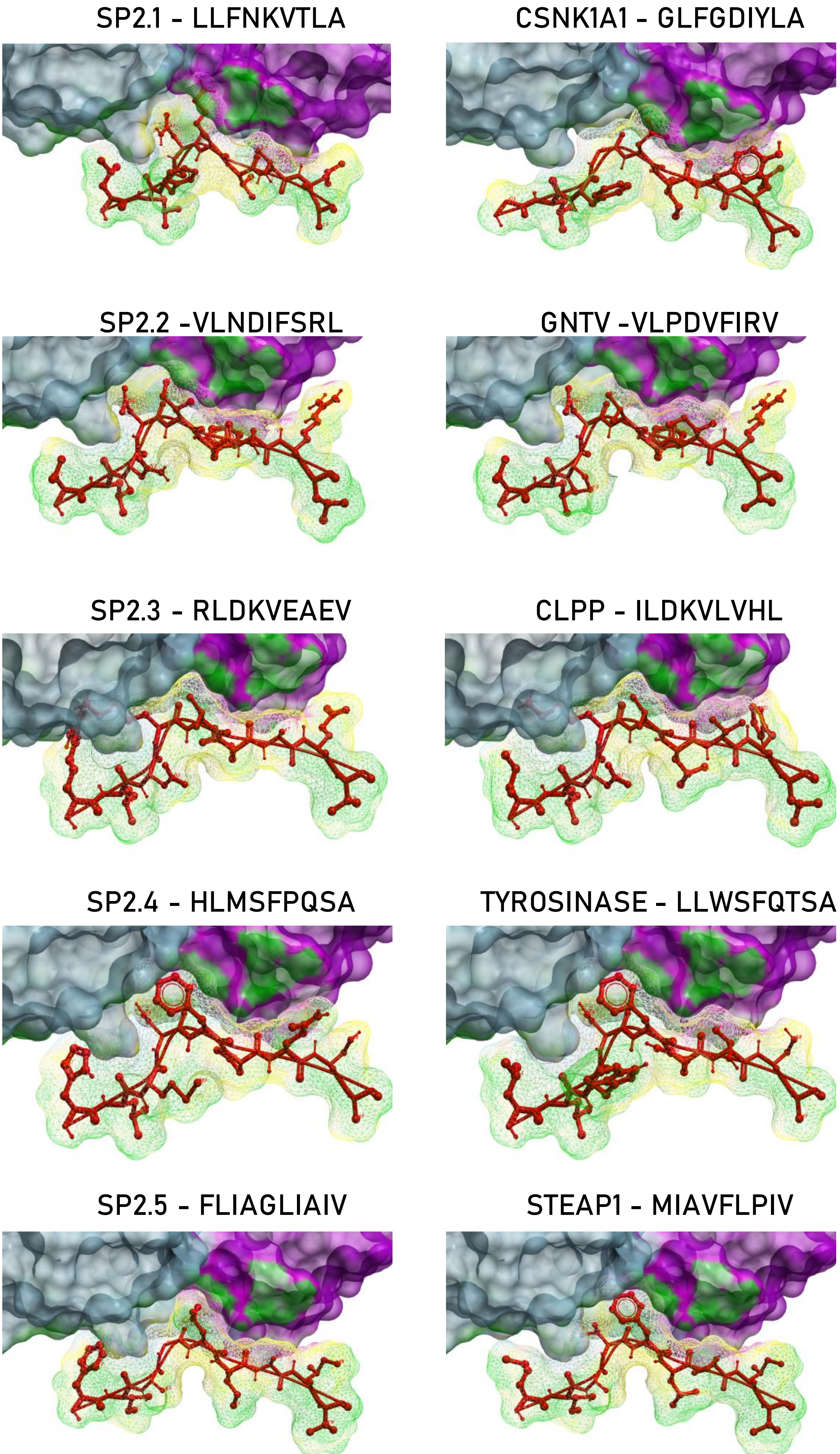
SP2.1 - LLFNKVTLA
CSNK1A1 - GLFGDIYLA
SP2.2 -VLNDIFSRL
GNTV -VLPDVFIRV
SP2.3 - RLDKVEAEV
CLPP - ILDKVLVHL
SP2.4 - HLMSFPQSA
TYROSINASE - LLWSFQTSA
SP2.5 - FLIAGLIAIV
STEAP1 - MIAVFLPIV

Suppl. Fig. 12

SP2.1 - LLFNKVTLA

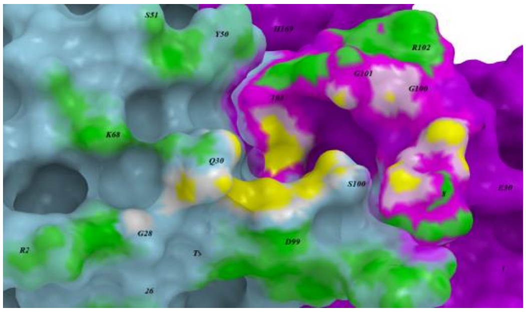

CSNK1A1 - GLFGDIYLA

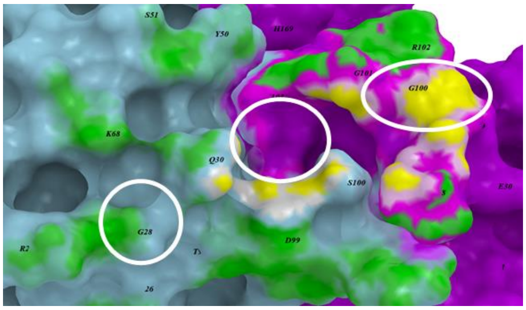

SP2.2 -VLNDIFSRL

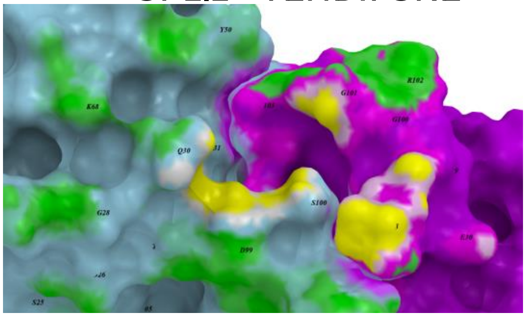

GNTV -VLPDVFIRV

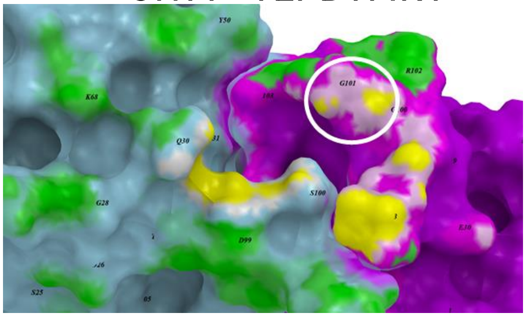

SP2.3 - RLDKVEAEV

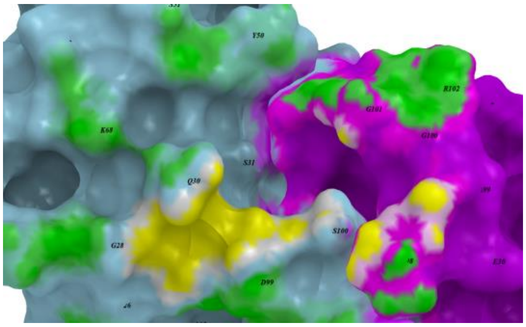

CLPP - ILDKVLVHL

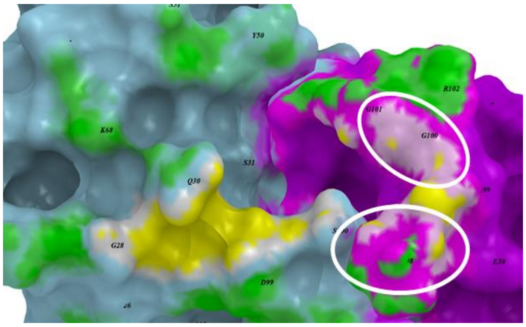

SP2.4 - HLMSFPQSA

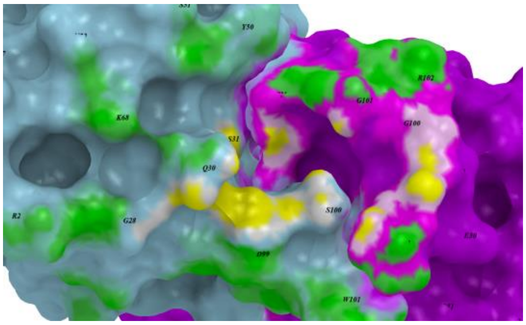

TYROSINASE - LLWSFQTSA

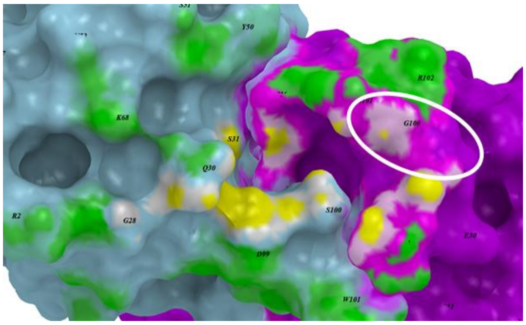

SP2.5 - FLIAGLIAIV

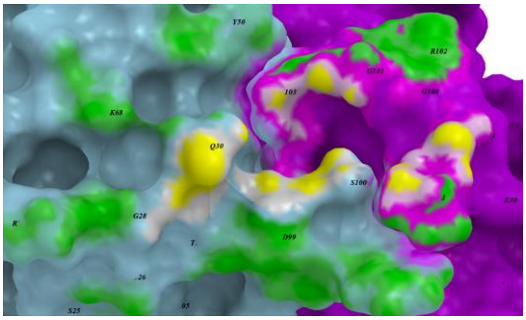

STEAP1 - MIAVFLPIV

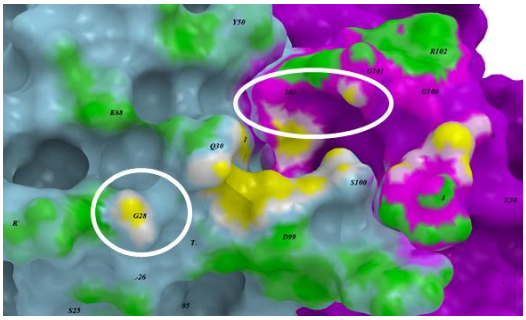

Suppl. Fig. 13

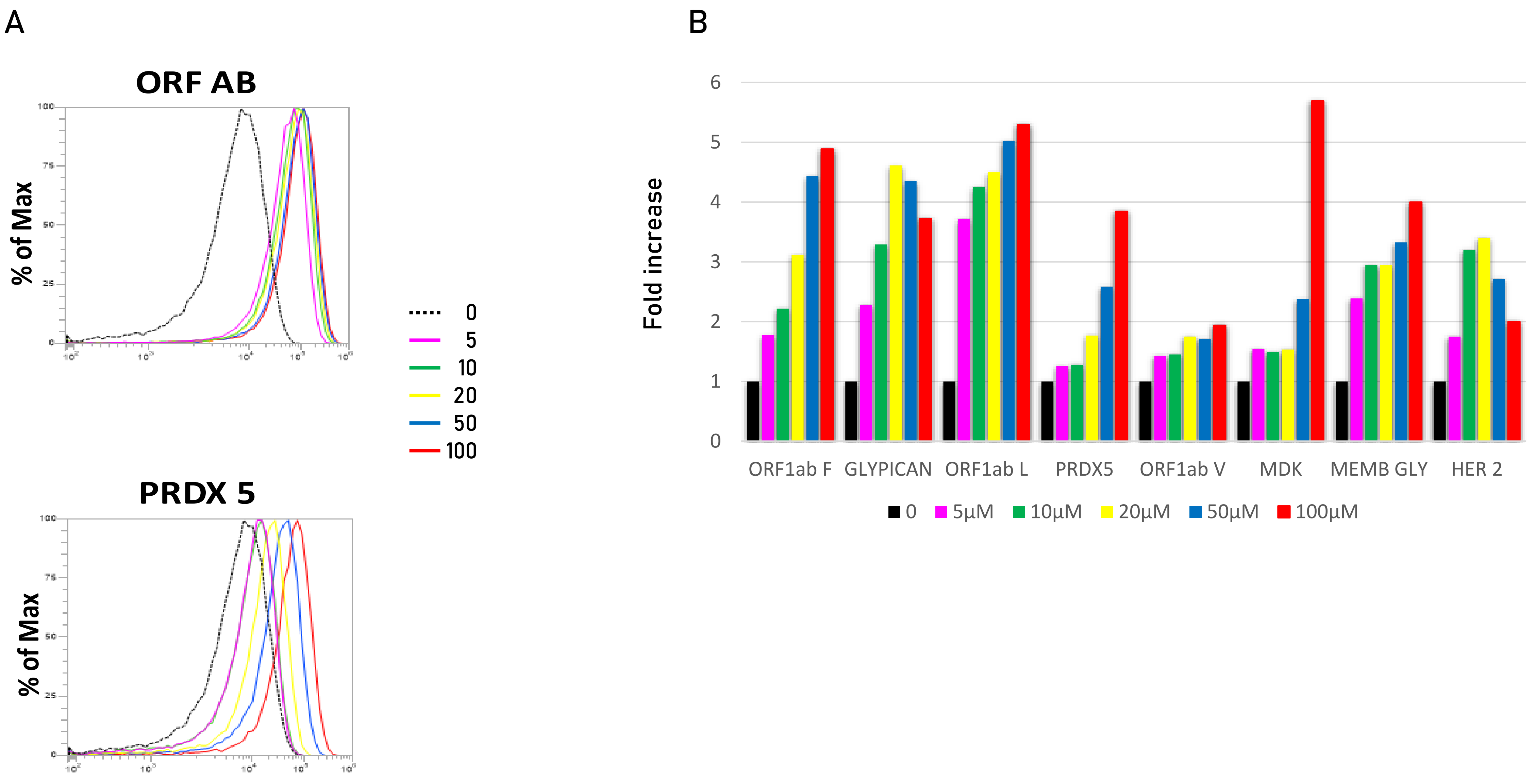
A
ORF AB
% of Max
PRDX 5
% of Max
0
5
10
20
50
100
B
Fold increase
0
1
2
3
4
5
6
ORF1ab F
GLYPICAN
ORF1ab L
PRDX5
ORF1ab V
MDK
MEMB GLY
HER 2
0
5μM
10μM
20μM
50μM
100μM

Suppl. Fig. 14

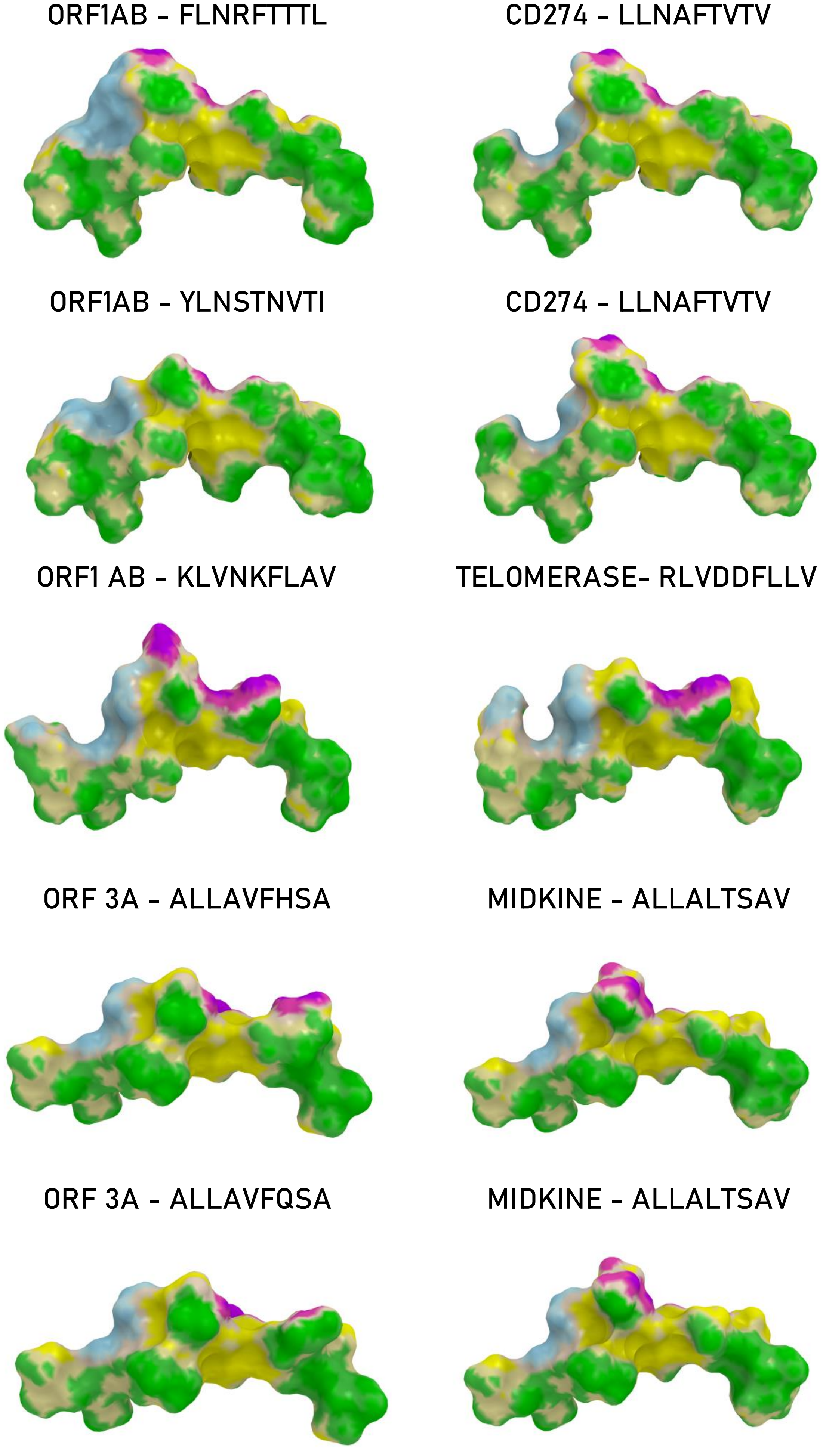
ORF1AB - FLNRFTTTL
CD274 - LLNAFTVTV
ORF1AB - YLNSTNVTI
CD274 - LLNAFTVTV
ORF1 AB - KLVNKFLAV
TELOMERASE- RLVDDFLLV
ORF 3A - ALLAVFHSA
MIDKINE - ALLALTSAV
ORF 3A - ALLAVFQSA
MIDKINE - ALLALTSAV

Suppl. Fig. 15

ORF 1AB- ALLSDLQDL

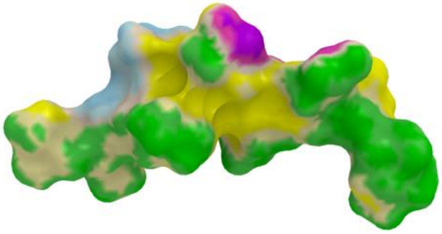

PRDX5- LLLDDLLVS

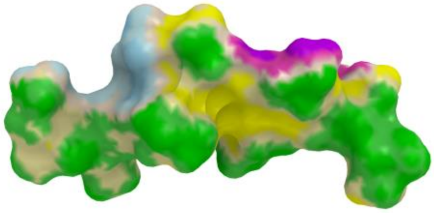

ORF 1AB- VLLAPLLSA

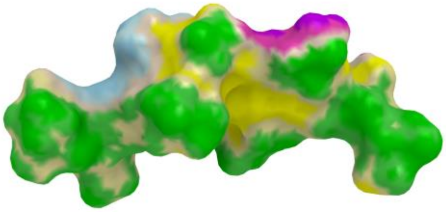

PRDX5- LLLDDLLVS

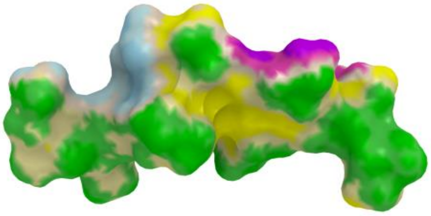

ORF 1AB- SLLSVLLSM

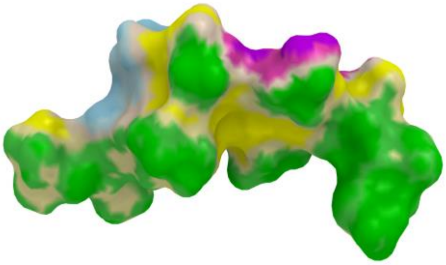

PRDX5- LLLDDLLVS

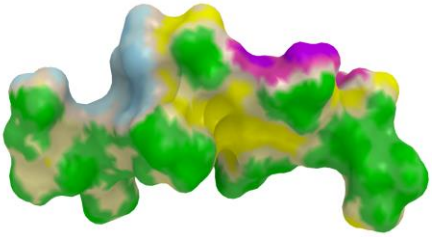

NUCLEOC- LLLDRLNQL

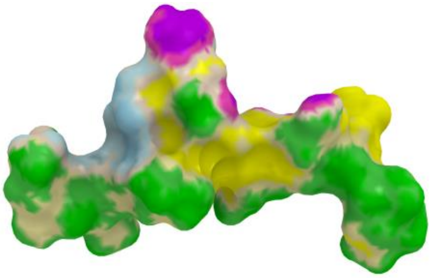

PRDX5- LLLDDLLVS

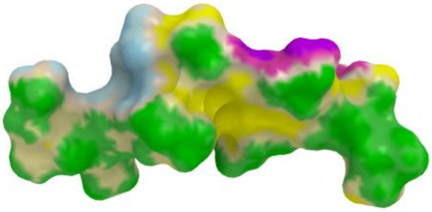

NUCLEOC -LLLDRLNQL

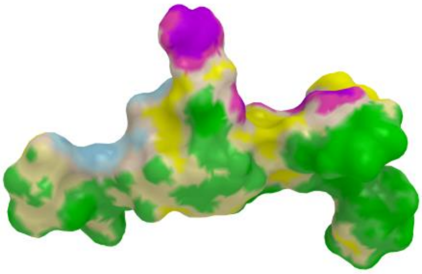

BING 4- CQWGRLWQL

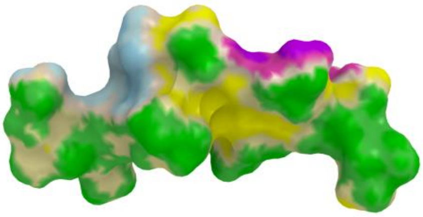

Suppl. Fig. 16

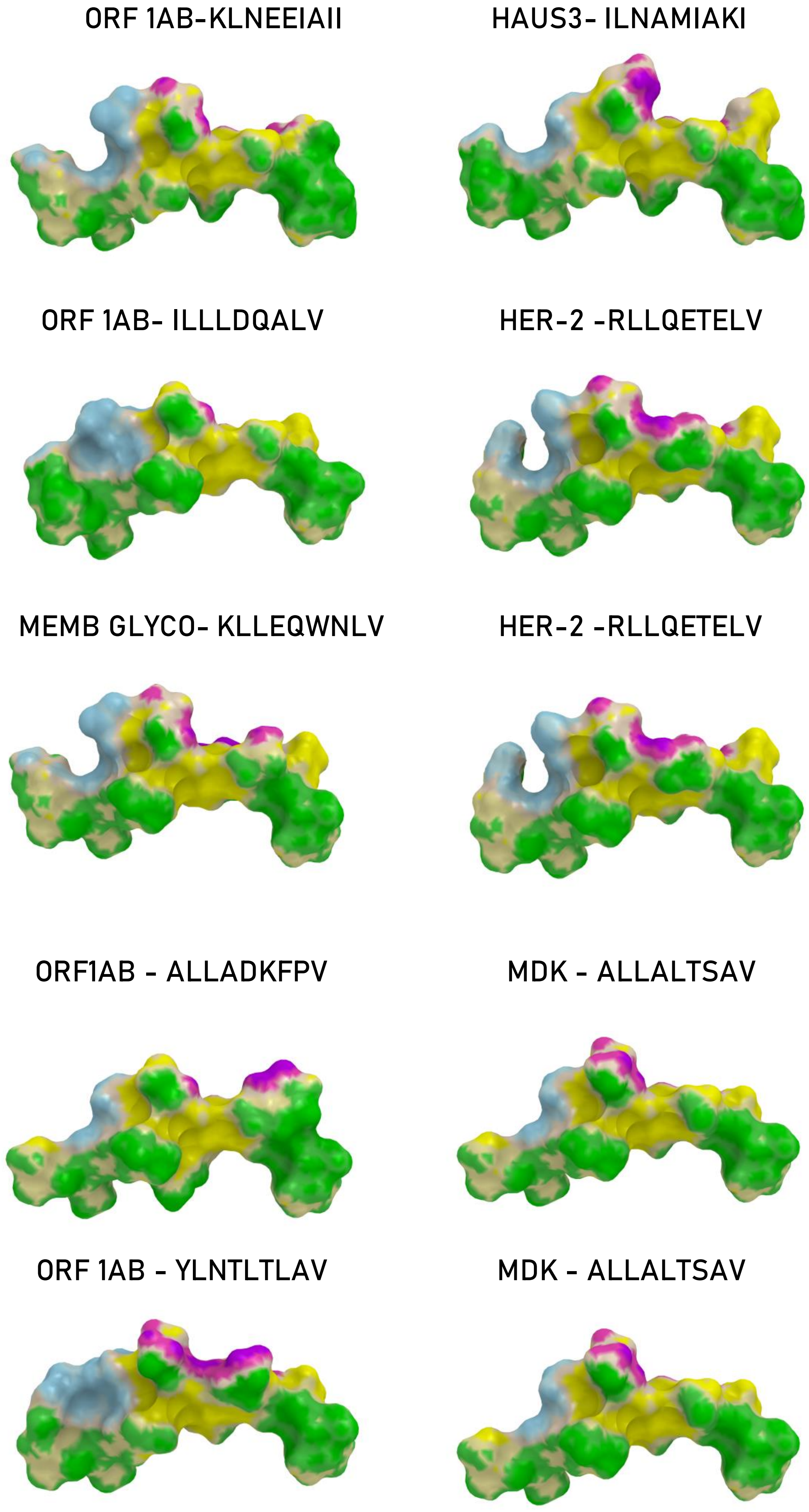
ORF 1AB-KLNEEIAII
HAUS3- ILNAMIAKI
ORF 1AB- ILLLDQALV
HER-2 -RLLQETELV
MEMB GLYCO- KLLEQWNLV
HER-2 -RLLQETELV
ORF1AB - ALLADKFPV
MDK - ALLALTSAV
ORF 1AB - YLNTLTLAV
MDK - ALLALTSAV

Suppl. Fig. 17

ORF 1AB - SLLMPILTL

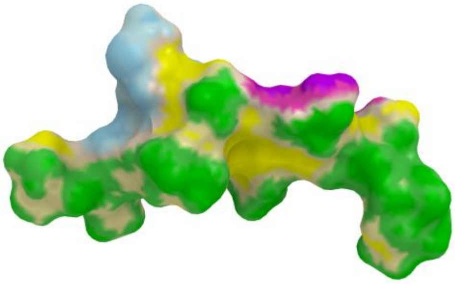

MUC1 - LLLLTVLTV

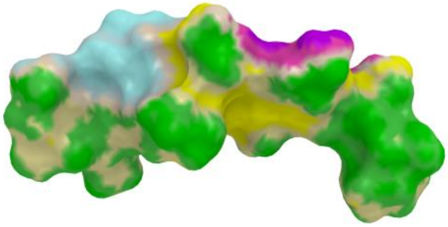

ORF 1AB - LLFLMSFTV

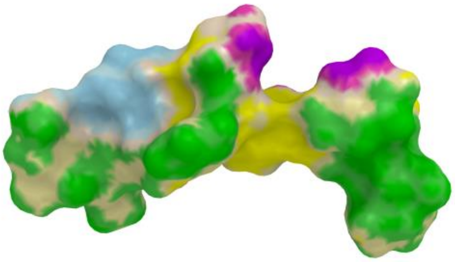

MUC1 - LLLLTVLTV

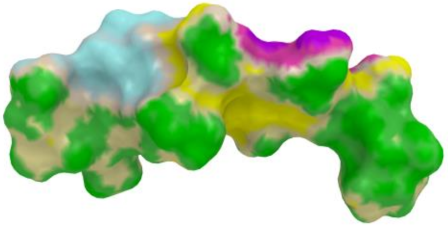

ORF 1AB- SLPGVFCGV

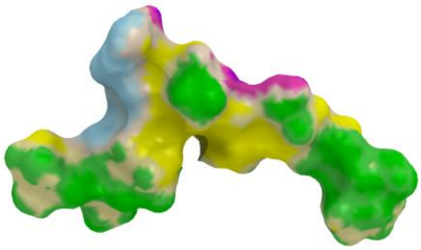

GNTV-VLPDVFIRV

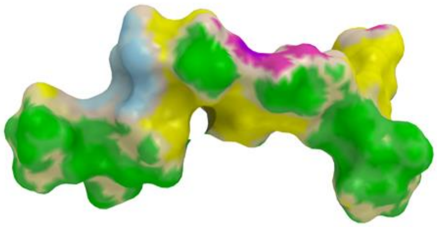

ORF 1AB- FLPRVFSAV

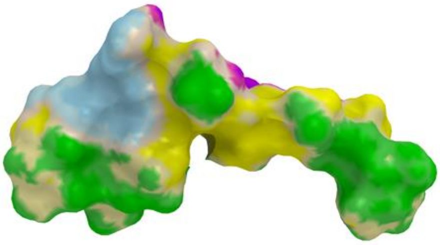

GNTV-VLPDVFIRV

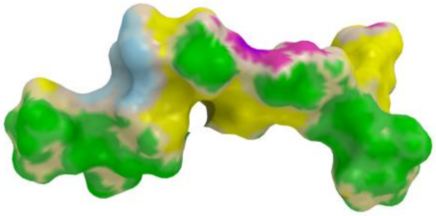

ORF 1 AB- LLLDDFVEI

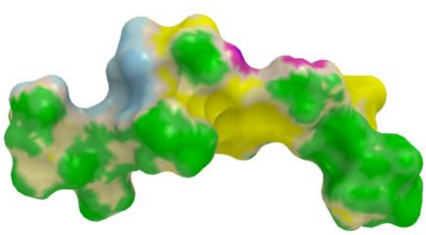

KIF20A- LLSDDDVVV

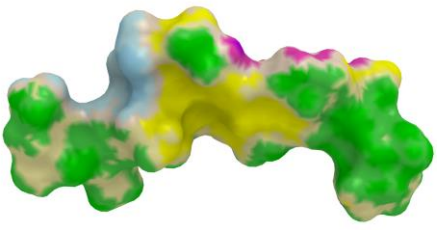

Suppl. Fig. 18

ORF 1 AB- ALWEIQQVV

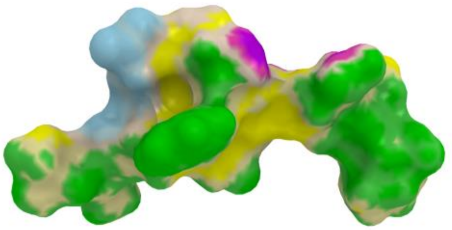

ID01- ALLEIASCL

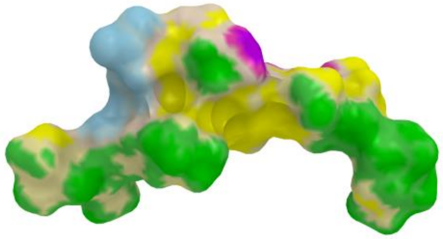

ORF 1AB - TLNDLNETL

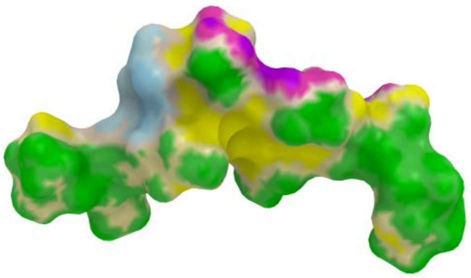

MELOE- TLNDECWPA

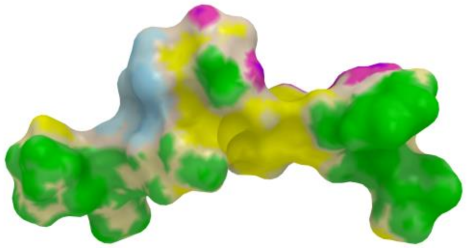

ORF 1AB- VLLAPLLSA

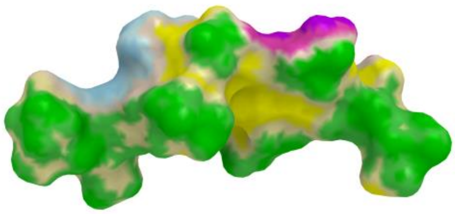

Nectin- 4 - VLVPPLPSL

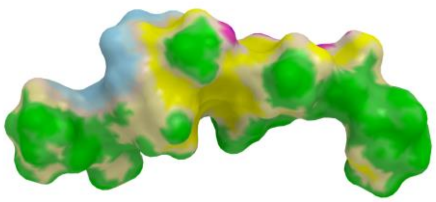

ORF 1AB- NVLTLVYKV

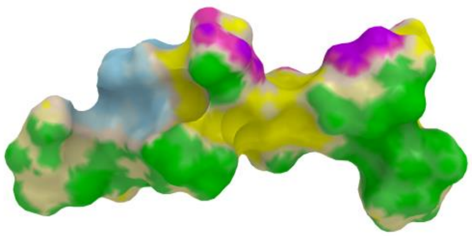

MAGE A1- KVLWYVIKV

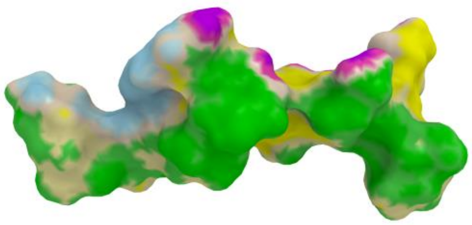

ORF 1AB- GVFCGVDAV

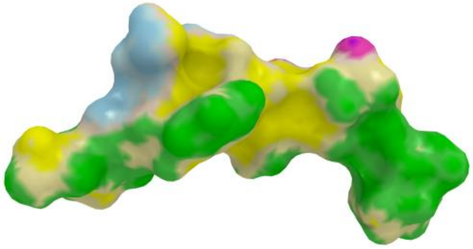

HEPSIN- GLQLGVQAV

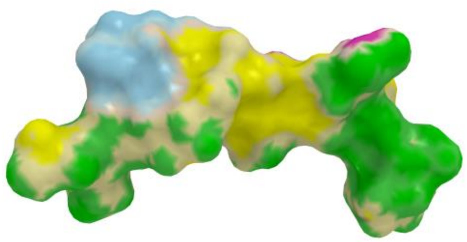

Suppl. Fig. 19

MEMB GLYCO - TLACFVLAA

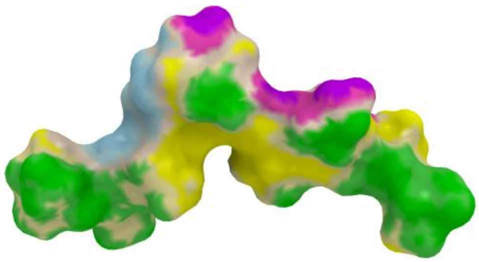

HEPCAM- RLAPFVYLL

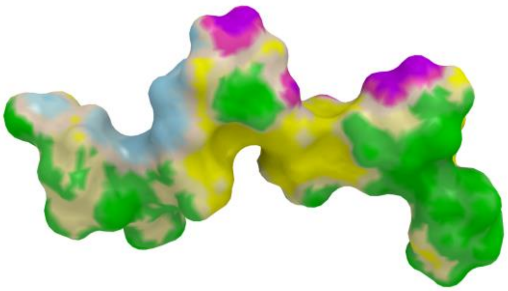

ORF7 -FLALITLAT

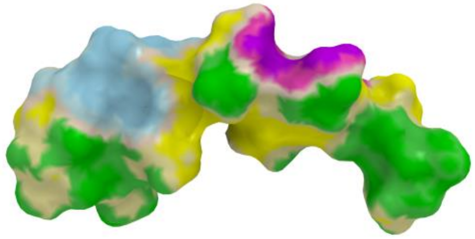

CALCA -FLALSILVL

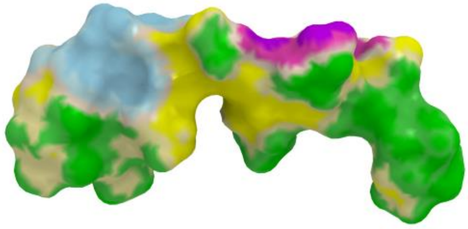

Suppl. Fig. 20

ORF1AB - FLNRFTTTL

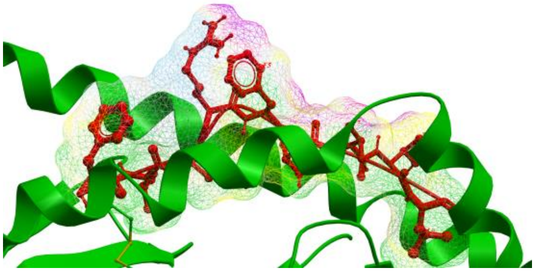

CD274 - LLNAFTVTV

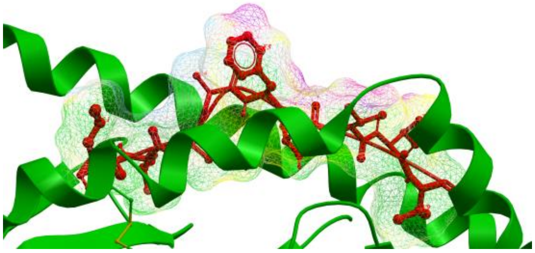

ORF1AB - YLNSTNVTI

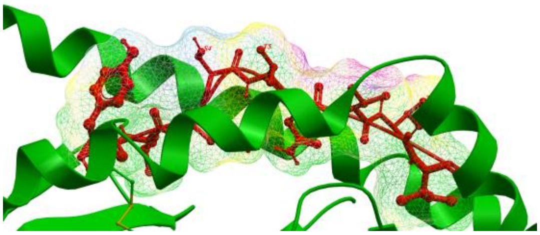

CD274 - LLNAFTVTV

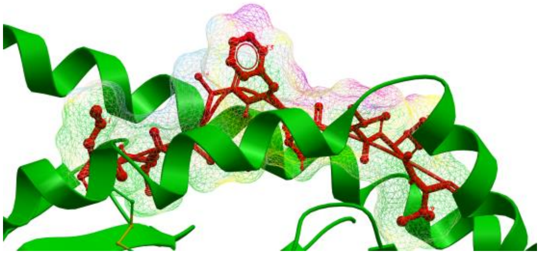

ORF1 AB - KLVNKFLAV

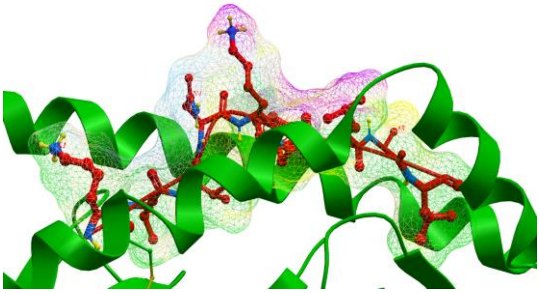

TELOMERASE- RLVDDFLLLV

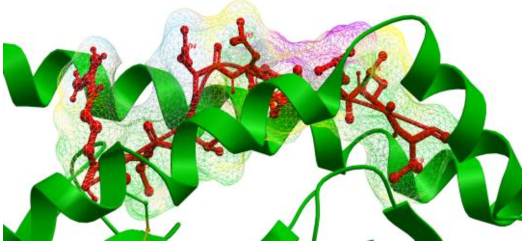

ORF 3A - ALLAVFHSA

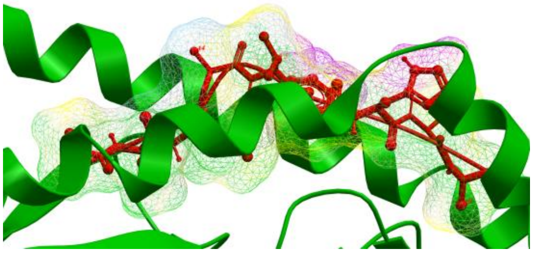

MIDKINE - ALLALTSAV

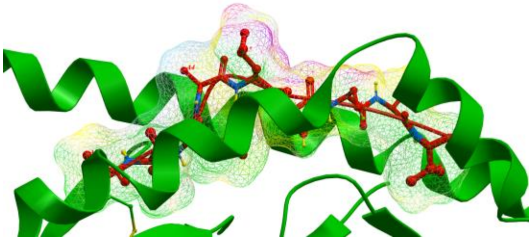

ORF 3A - ALLAVFQSA

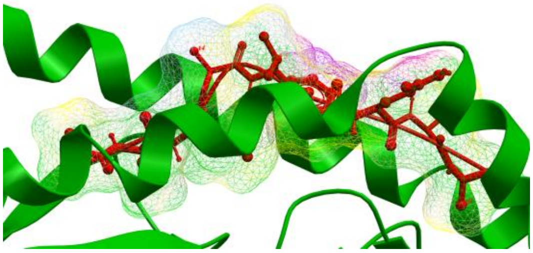

MIDKINE - ALLALTSAV

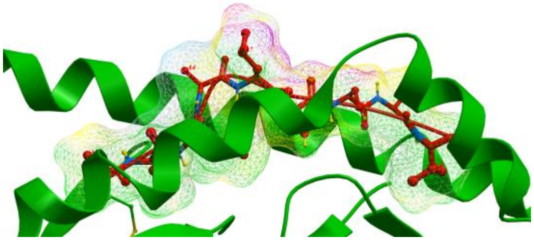

Suppl. Fig. 21

ORF1AB - FLNRFTTTL

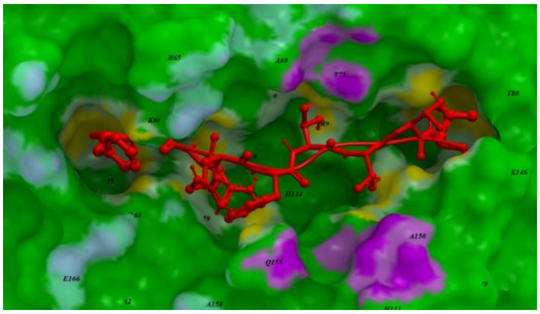

CD274 - LLNAFTVTV

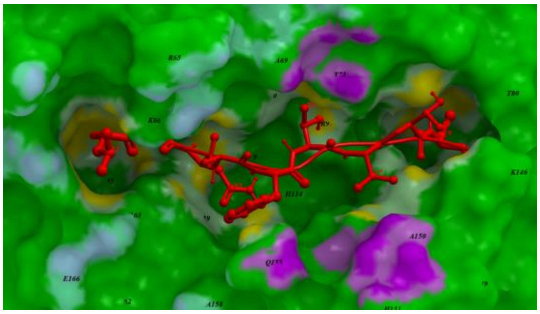

ORF1AB - YLNSTNVTI

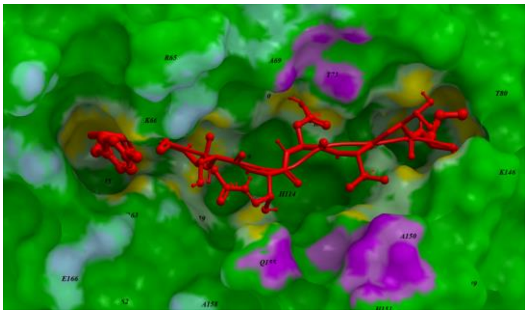

CD274 - LLNAFTVTV

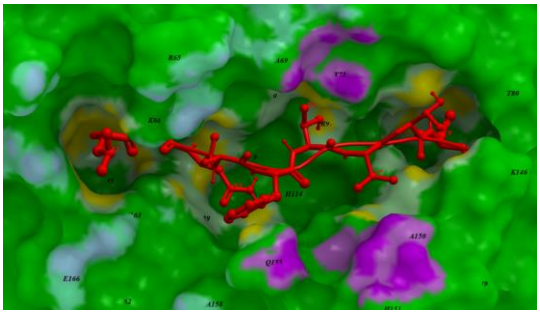

ORF1 AB - KLVNKFLAV

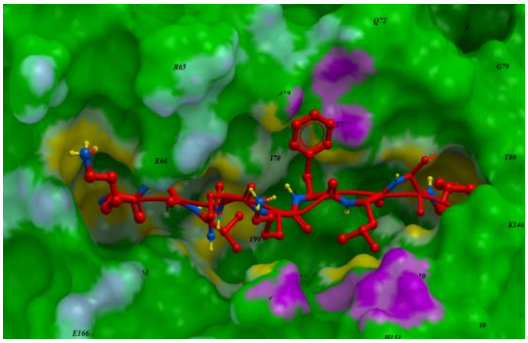

TELOMERASE- RLVDDFLLV

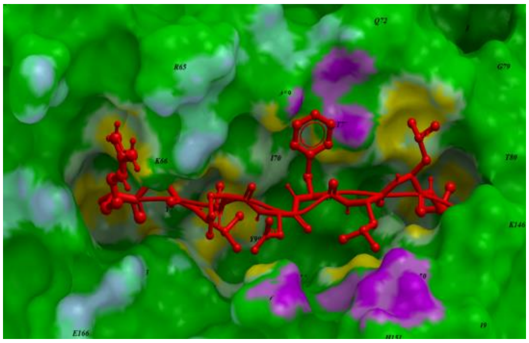

ORF 3A - ALLAVFHSA

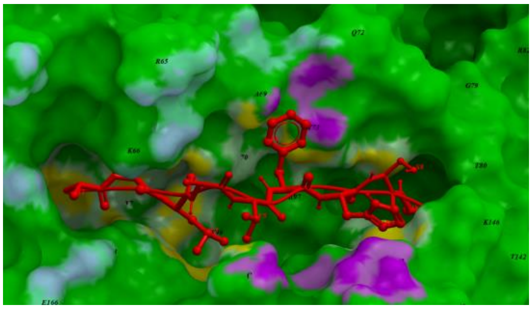

MIDKINE - ALLALTSAV

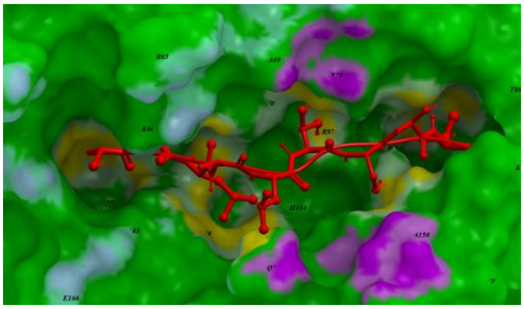

ORF 3A - ALLAVFQSA

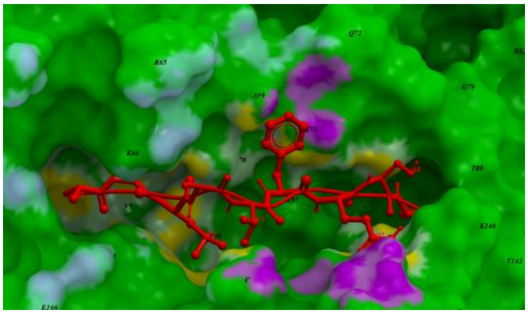

MIDKINE - ALLALTSAV

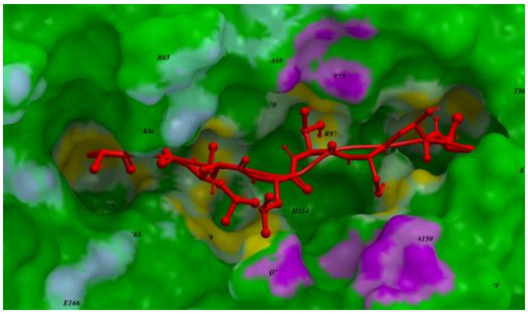

Suppl. Fig. 22

ORF 1AB- ALLSDLQDL

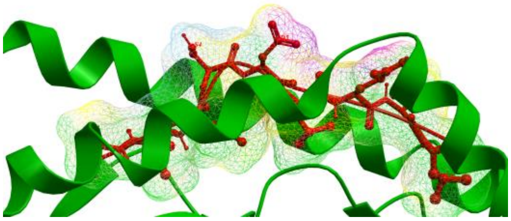

PRDX5- LLLDDLLVS

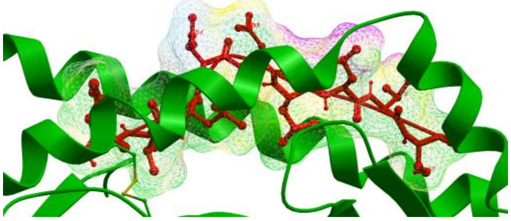

ORF 1AB- VLLAPLLSA

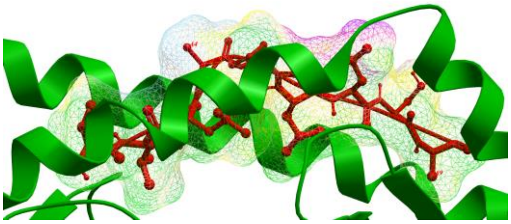

PRDX5- LLLDDLLVS

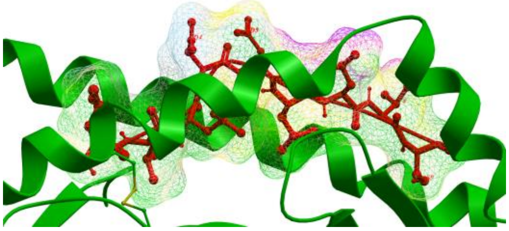

ORF 1AB- SLLSVLLSM

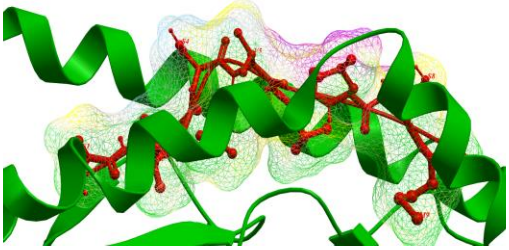

PRDX5- LLLDDLLVS

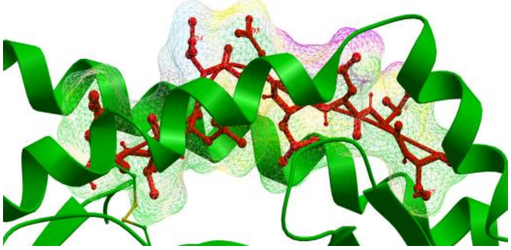

NUCLEOC- LLLDRLNQL

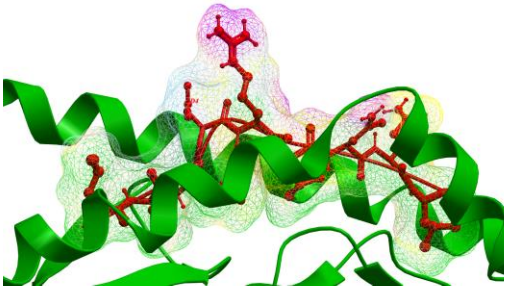

PRDX5- LLLDDLLVS

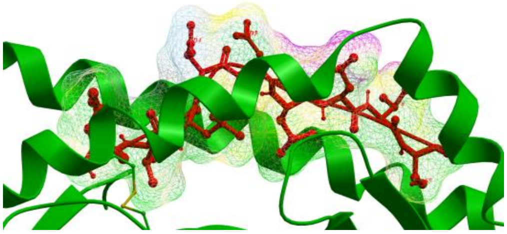

NUCLEOC -LLLDRLNQL

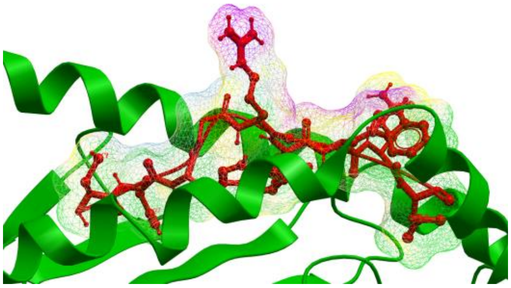

BING 4- CQWGRLWQL

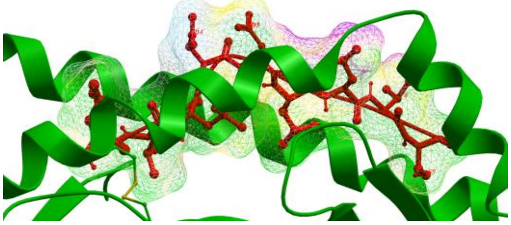

Suppl. Fig. 23

ORF 1AB- ALLSDLQDL

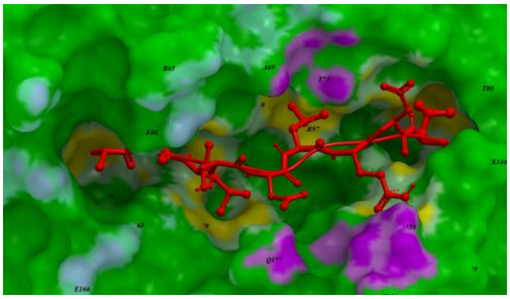

PRDX5- LLLDDLLVS

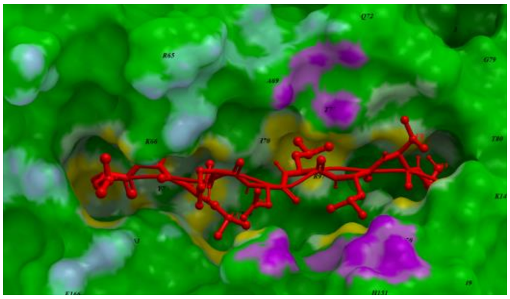

ORF 1AB- VLLAPLLSA

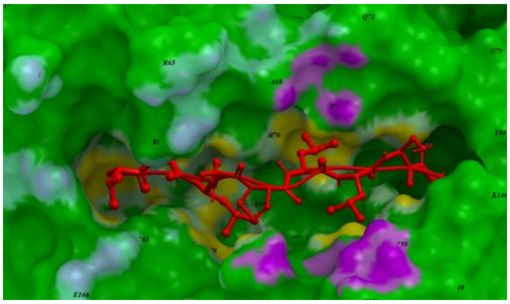

PRDX5- LLLDDLLVS

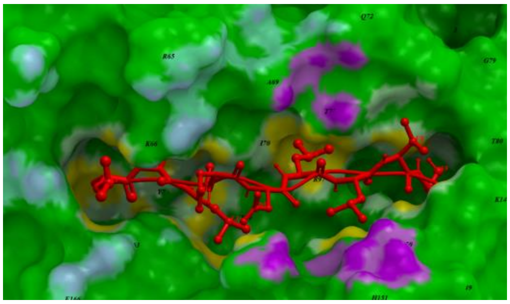

ORF 1AB- SLLSVLLSM

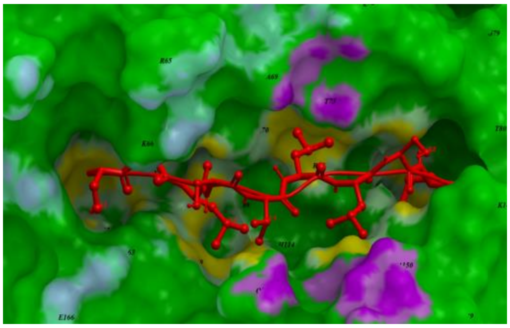

PRDX5- LLLDDLLVS

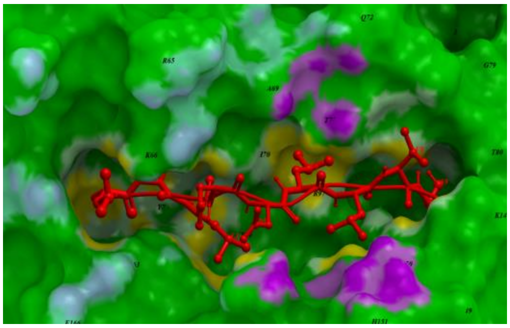

NUCLEOC- LLLDRLNQL

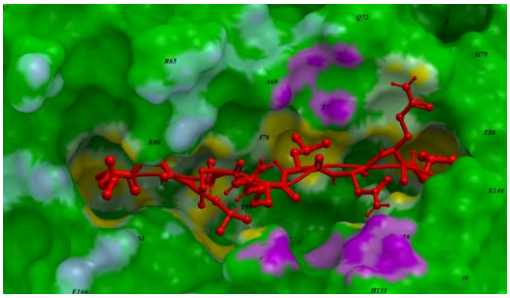

PRDX5- LLLDDLLVS

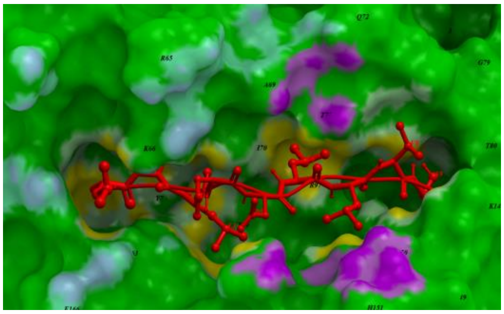

NUCLEOC -LLLDRLNQL

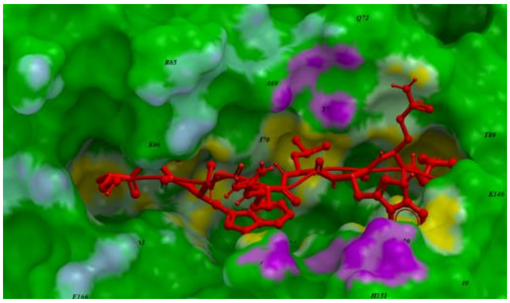

BING 4- CQWGRLWQL

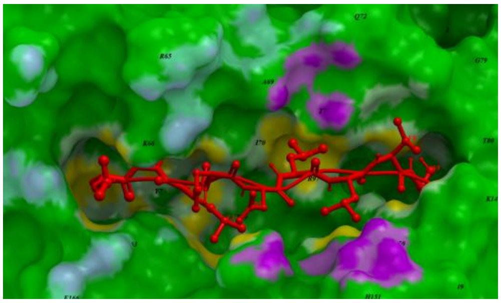

Suppl. Fig. 24

ORF 1AB-KLNEEIAII

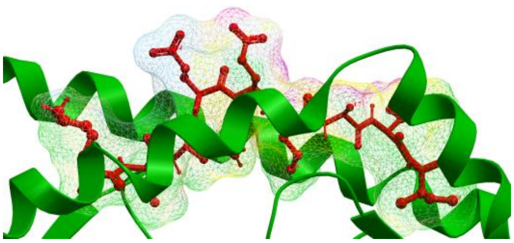

HAUS3- ILNAMIAKI

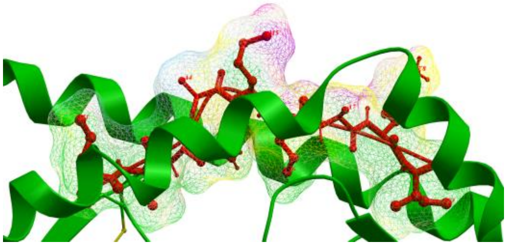

ORF 1AB- ILLLDQALV

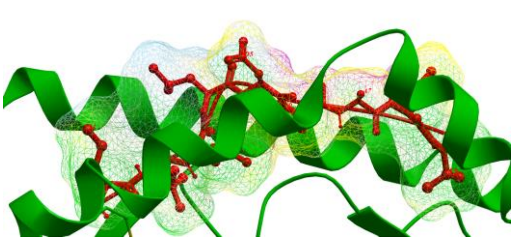

HER-2 -RLLQETELV

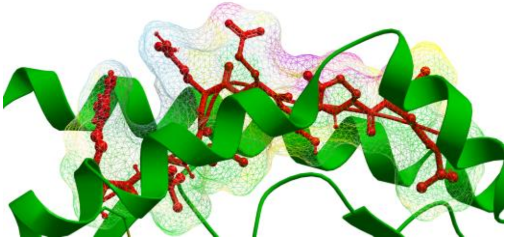

MEMB GLYCO- KLLEQWNLV

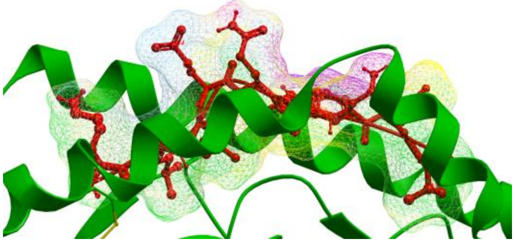

HER-2 -RLLQETELV

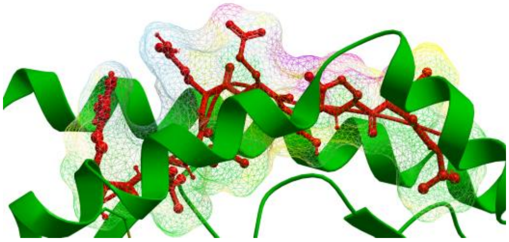

ORF1AB - ALLADKFPV

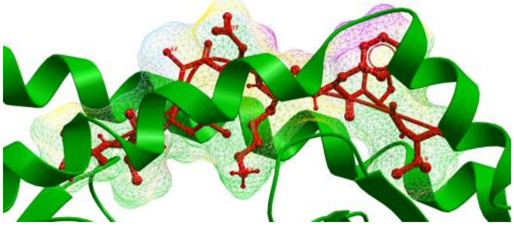

MDK - ALLALTSAV

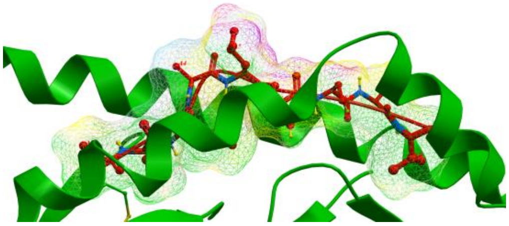

ORF 1AB - YLNTLTLAV

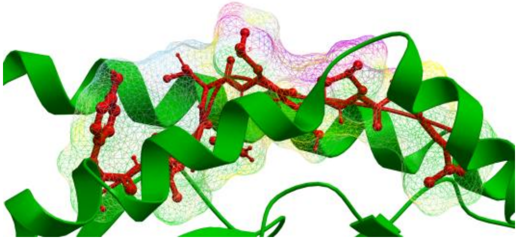

MDK - ALLALTSAV

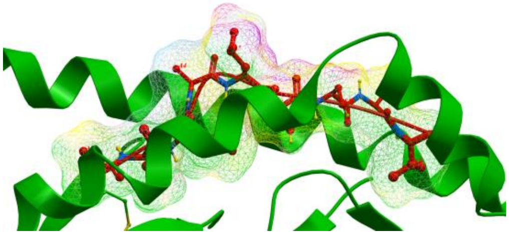

Suppl. Fig. 25

ORF 1AB-KLNEEIAII

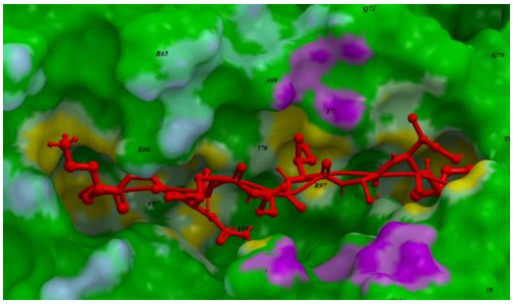

HAUS3- ILNAMIAKI

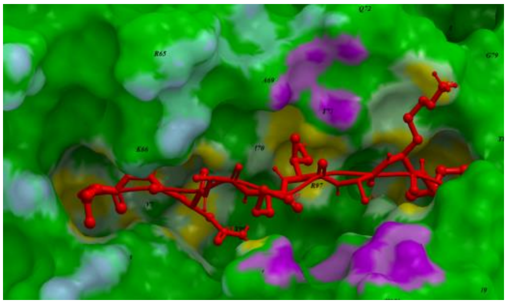

ORF 1AB- ILLLDQALV

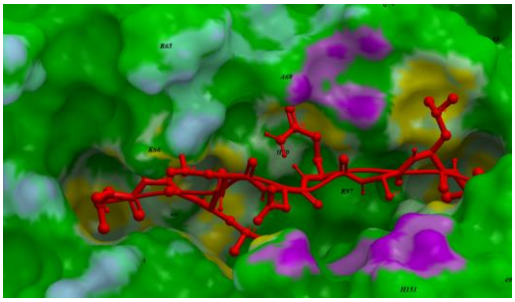

HER-2 -RLLQETELV

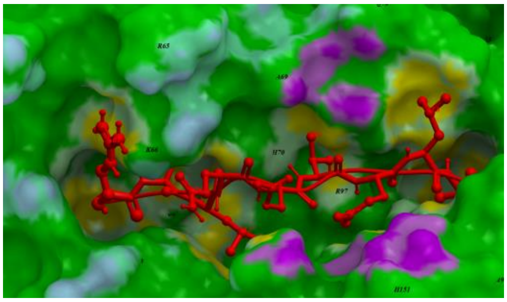

MEMB GLYCO- KLLEQWNLV

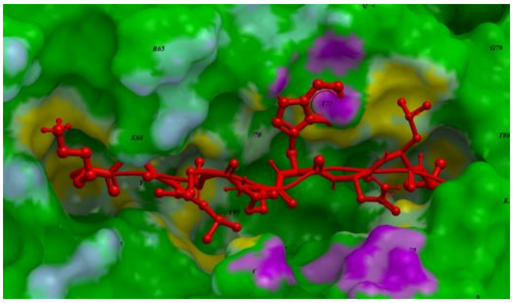

HER-2 -RLLQETELV

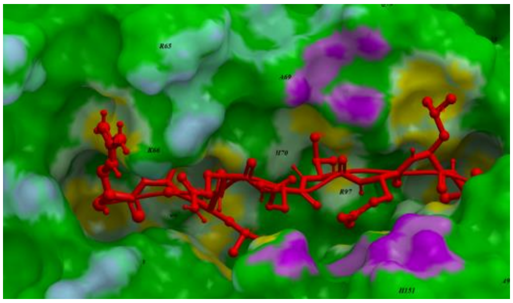

ORF1AB - ALLADKFPV

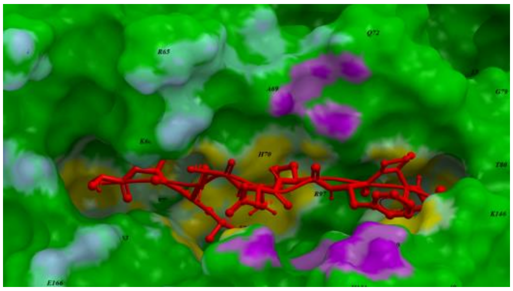

MDK - ALLALTSAV

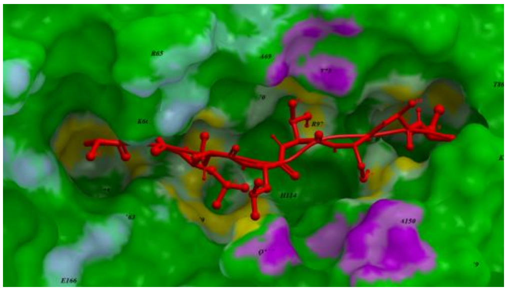

ORF 1AB - YLNTLTLAV

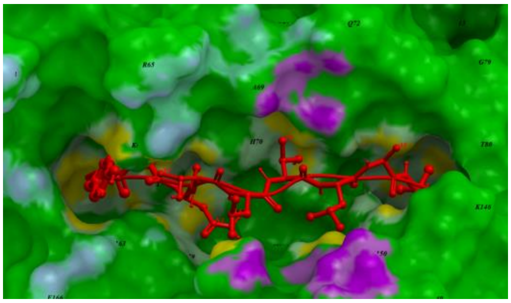

MDK - ALLALTSAV

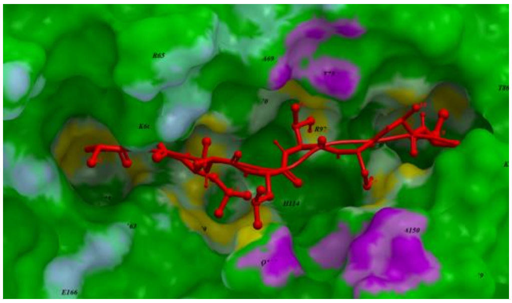

Suppl. Fig. 26

ORF 1AB - SLLMPILTL

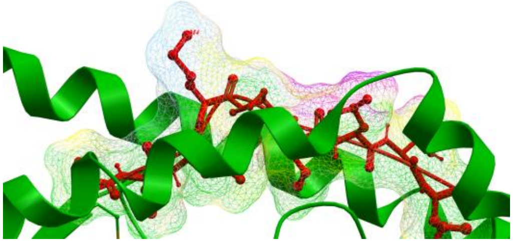

MUC1 - LLLLTVLTV

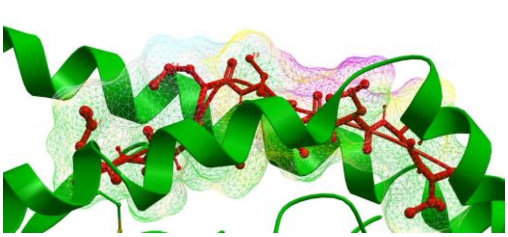

ORF 1AB - LLFLMSFTV

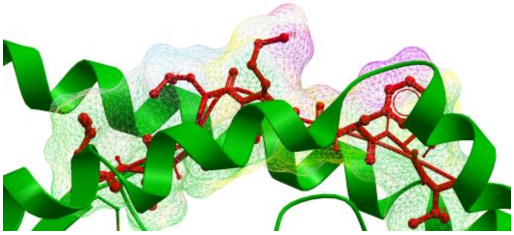

MUC1 - LLLLTVLTV

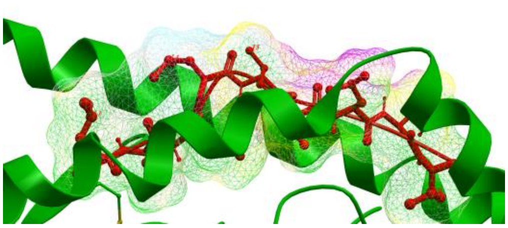

ORF 1AB- SLPGVFCGV

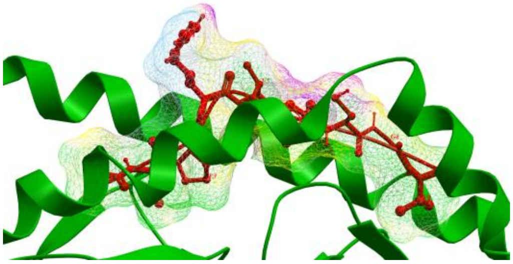

GNTV-VLPDVFIRV

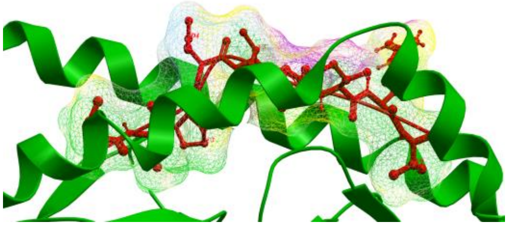

ORF 1AB- FLPRVFSAV

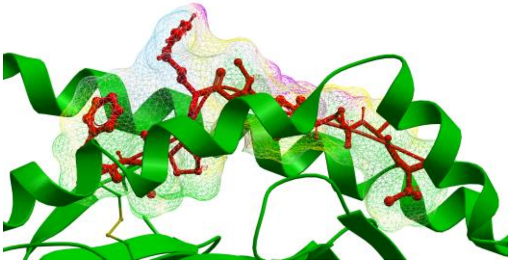

GNTV-VLPDVFIRV

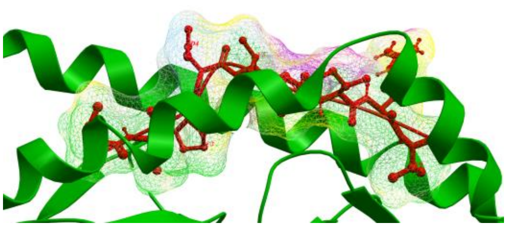

ORF 1 AB- LLLDDFVEI

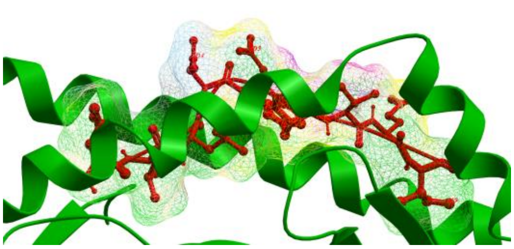

KIF20A- LLSDDDVVV

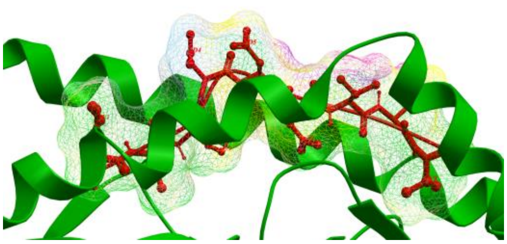

Suppl. Fig. 27

ORF 1AB - SLLMPILTL

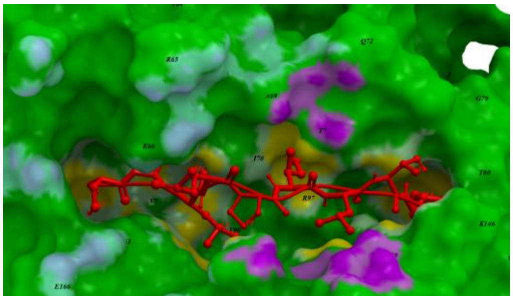

MUC1 - LLLLTVLTV

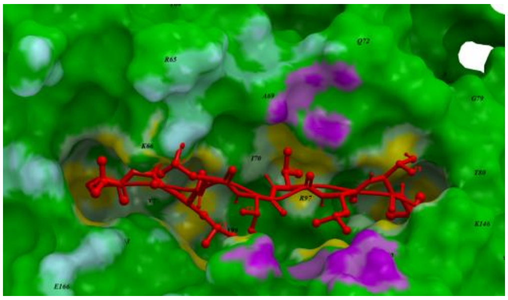

ORF 1AB - LLFLMSFTV

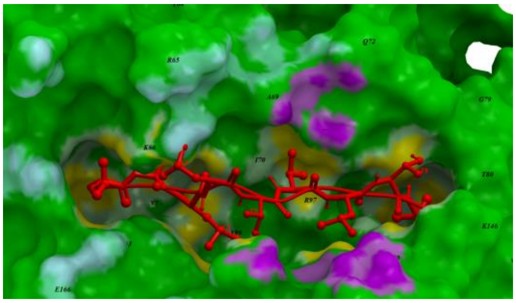

MUC1 - LLLLTVLTV

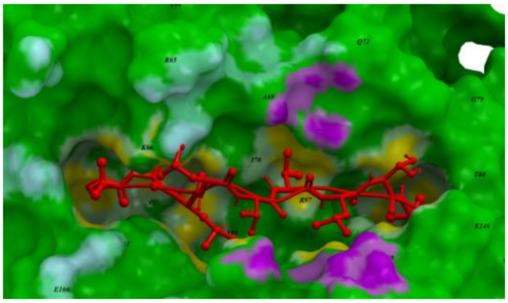

ORF 1AB- SLPGVFCGV

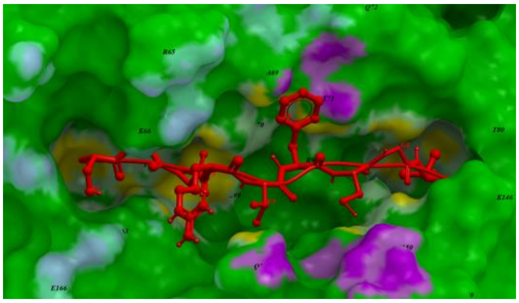

GNTV-VLPDVFIRV

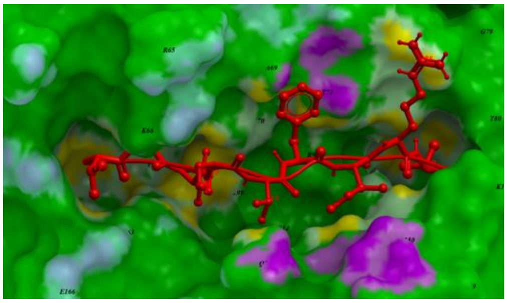

ORF 1AB- FLPRVFSAV

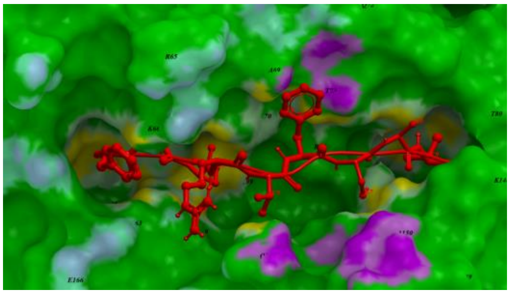

GNTV-VLPDVFIRV

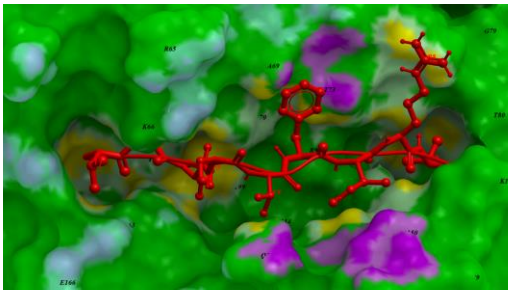

ORF 1 AB- LLLDDFVEI

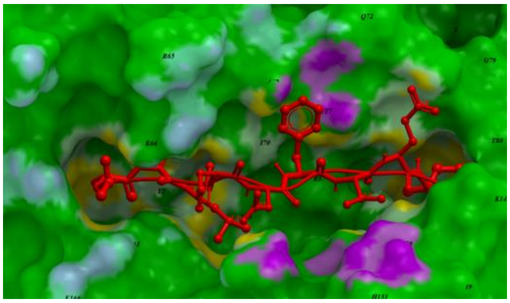

KIF20A- LLSDDDVVV

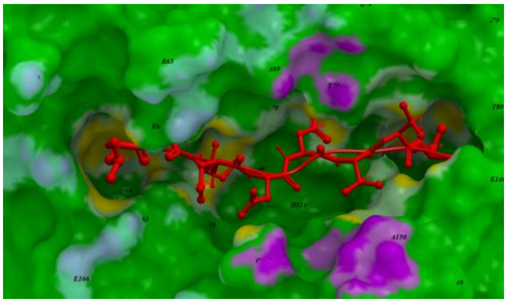

Suppl. Fig. 28

ORF 1 AB- ALWEIQQVV

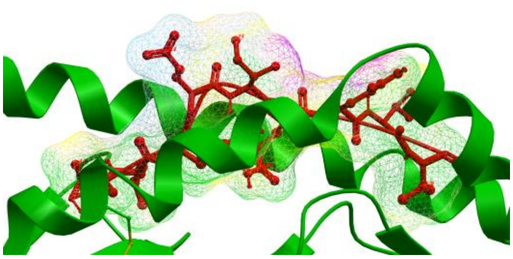

ID01- ALLEIASCL

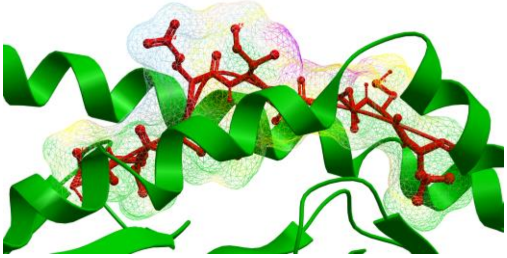

ORF 1AB - TLNDLNETL

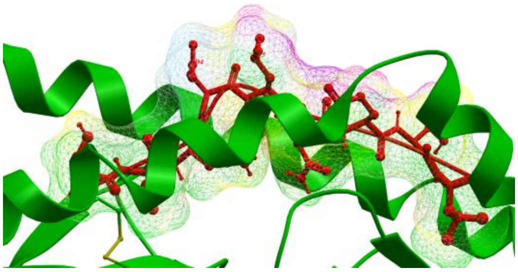

MELOE- TLNDECWPA

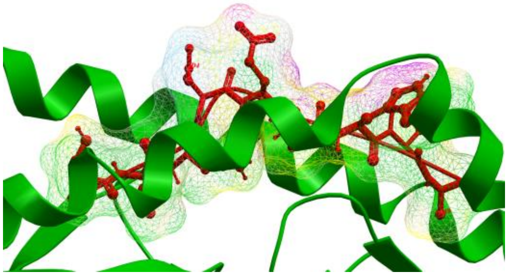

ORF 1AB- VLLAPLLSA

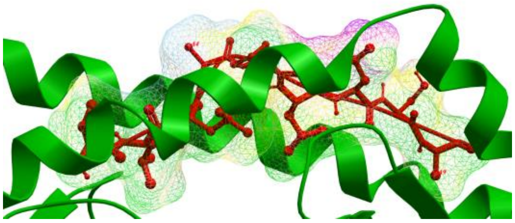

Nectin- 4 - VLVPPLPSL

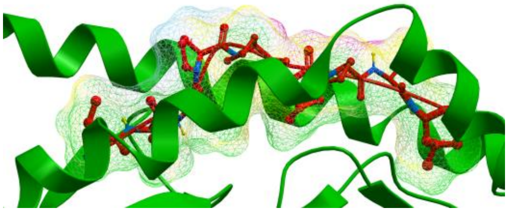

ORF 1AB- NVLTLVYKV

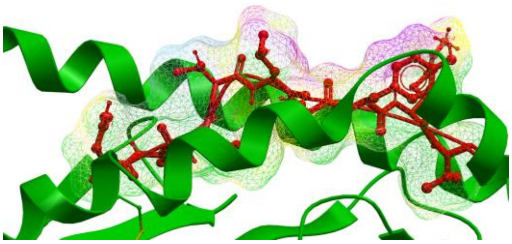

MAGE A1- KVLWYVIKV

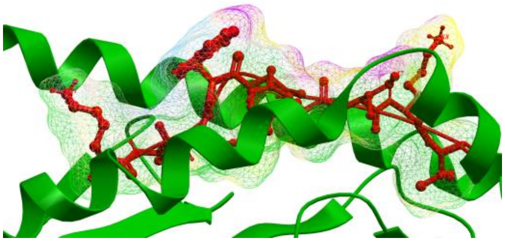

ORF 1AB- GVFCGVDAV

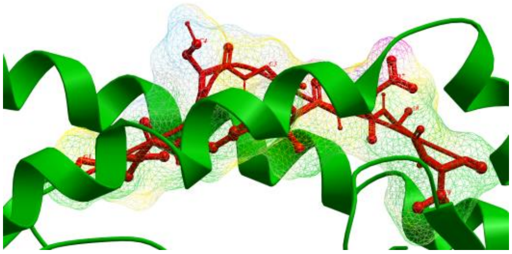

HEPSIN- GLQLGVQAV

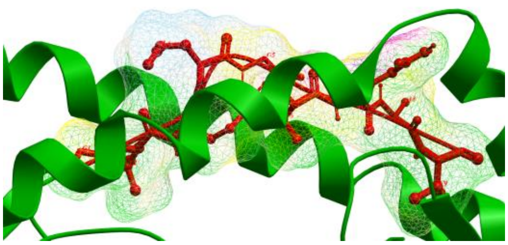

Suppl. Fig. 29

ORF 1 AB- ALWEIQQVV

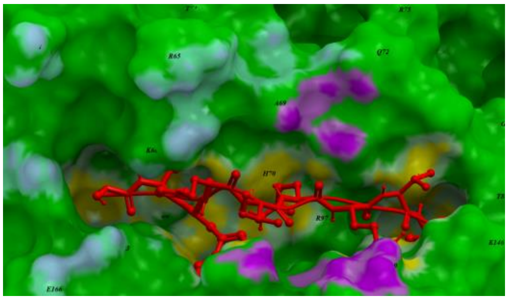

ID01- ALLEIASCL

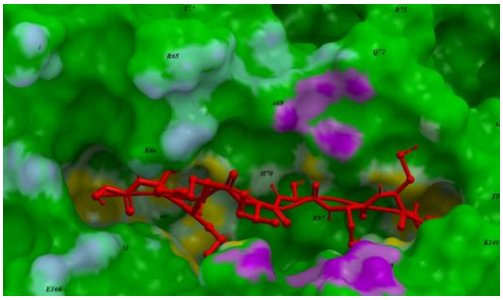

ORF 1AB - TLNDLNETL

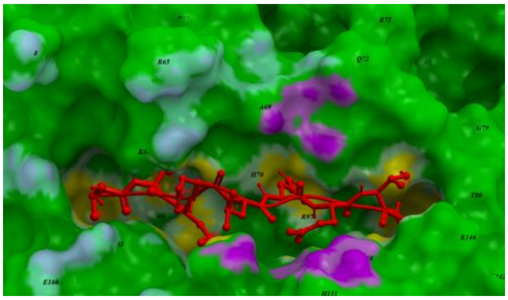

MELOE- TLNDECWPA

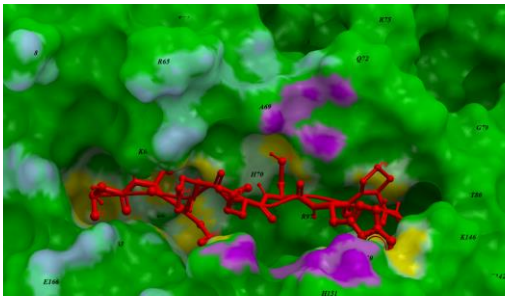

ORF 1AB- VLLAPLLSA

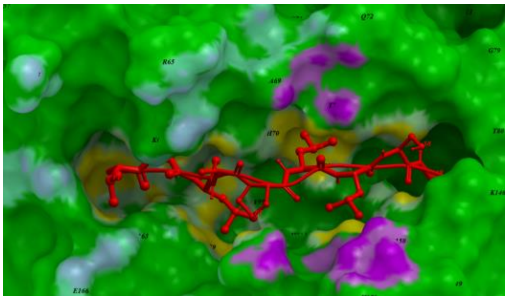

Nectin- 4 - VLVPPLPSL

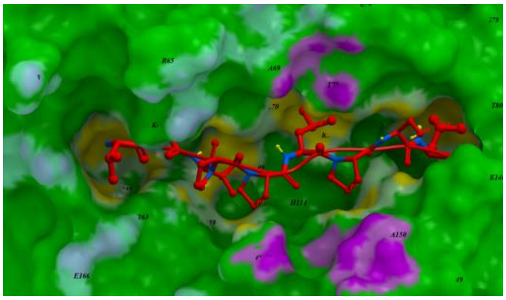

ORF 1AB- NVLTLVYKV

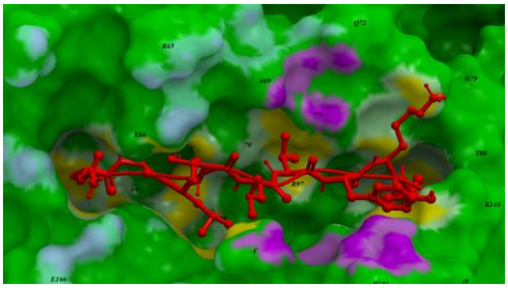

MAGE A1- KVLWYVIKV

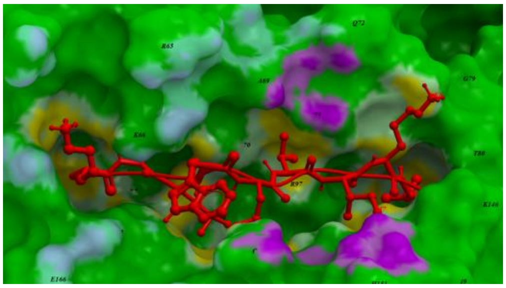

ORF 1AB- GVFCGVDAV

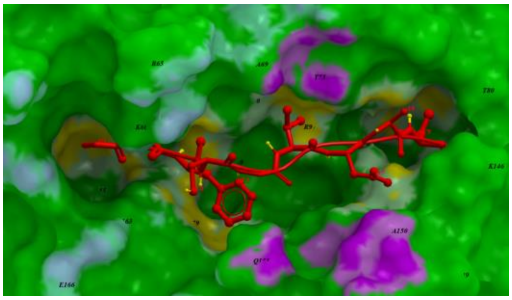

HEPSIN- GLQLGVQAV

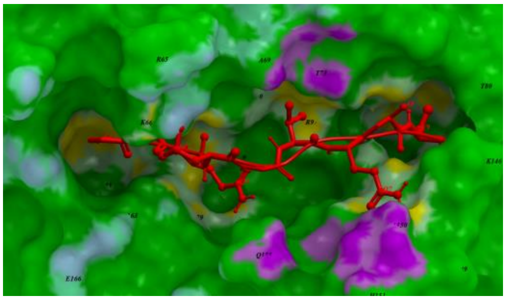

Suppl. Fig. 30

MEMB GLYCO - TLACFVLAA

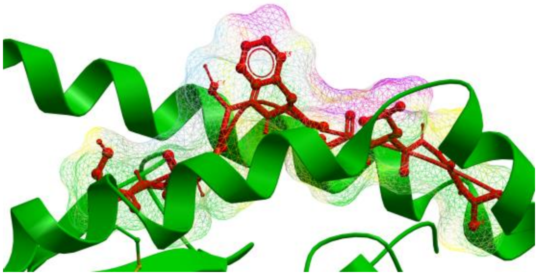

HEPCAM- RLAPFVYLL

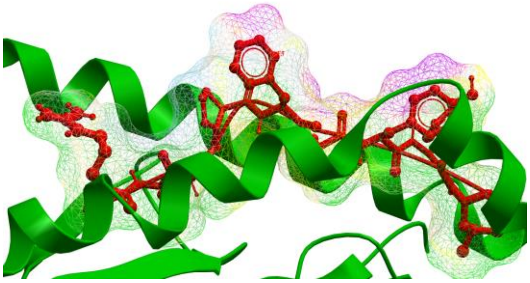

ORF7 -FLALITLAT

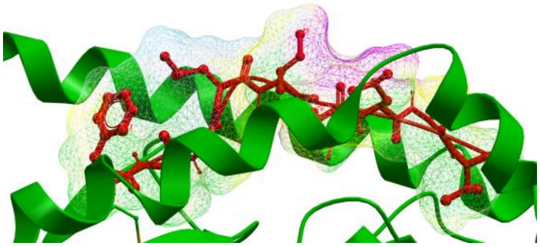

CALCA -FLALSILVL

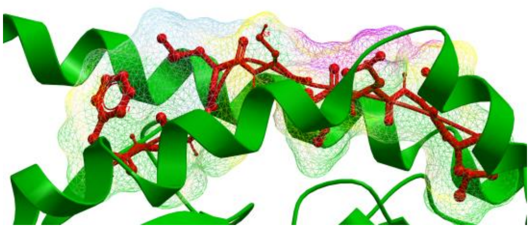

Suppl. Fig. 31

MEMB GLYCO - TLACFVLAA

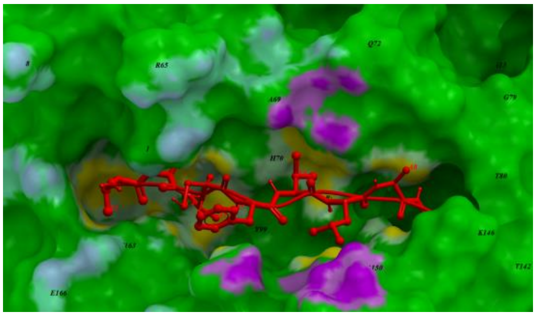

HEPCAM- RLAPFVYLL

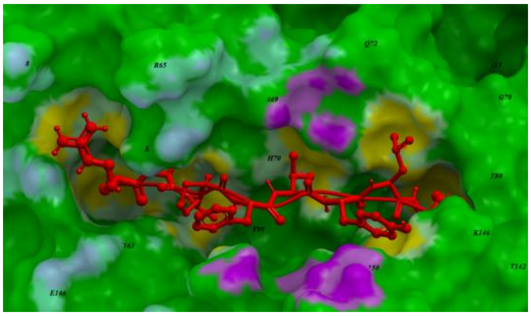

ORF7 -FLALITLAT

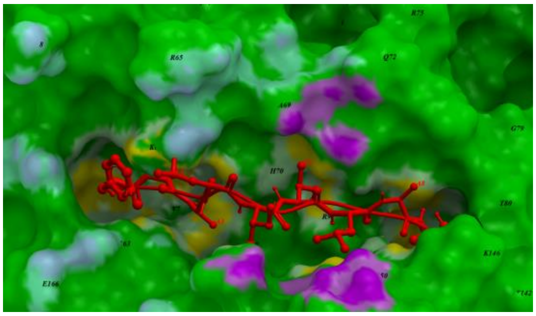

CALCA -FLALSILVL

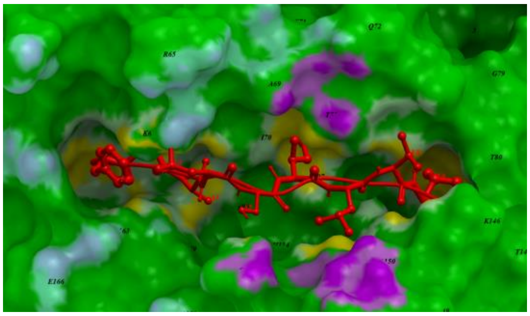

Suppl. Fig. 32

ORF1AB - FLNRFTTTL

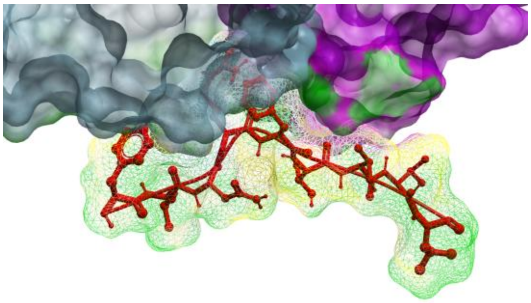

CD274 - LLNAFTVTV

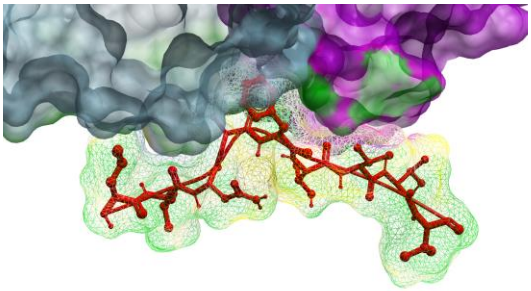

ORF1AB - YLNSTNVTI

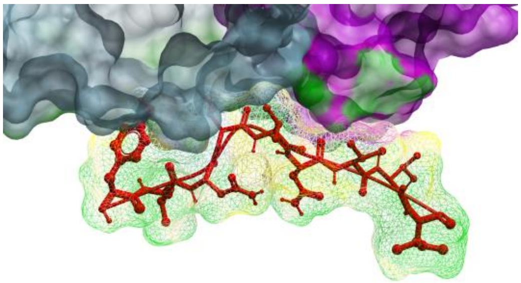

CD274 - LLNAFTVTV

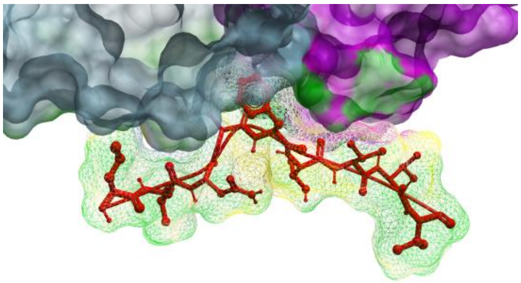

ORF1 AB - KLVNKFLAV

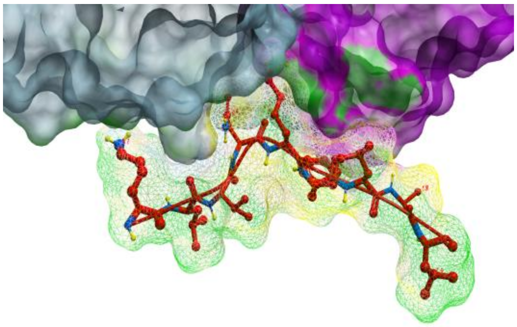

TELOMERASE- RLVDDFLLV

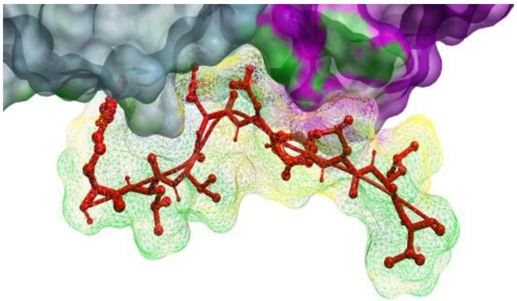

ORF 3A - ALLAVFHSA

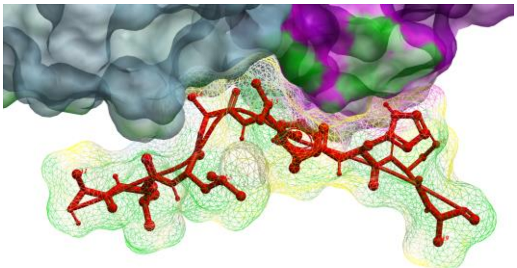

MIDKINE - ALLALTSAV

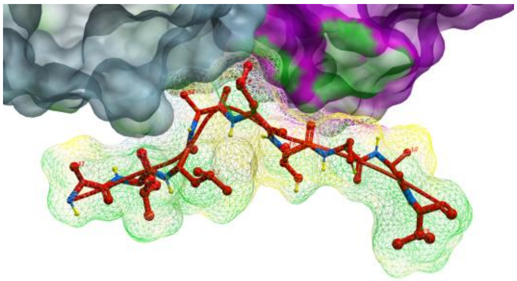

ORF 3A - ALLAVFQSA

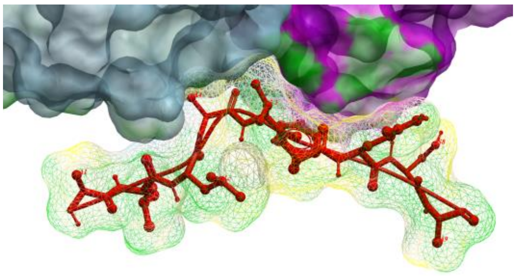

MIDKINE - ALLALTSAV

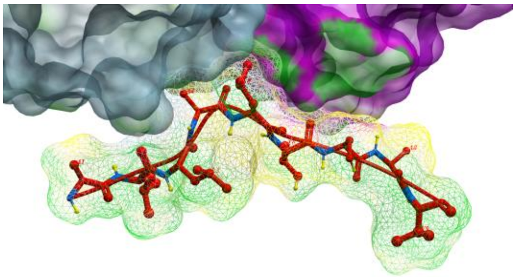

Suppl. Fig. 33

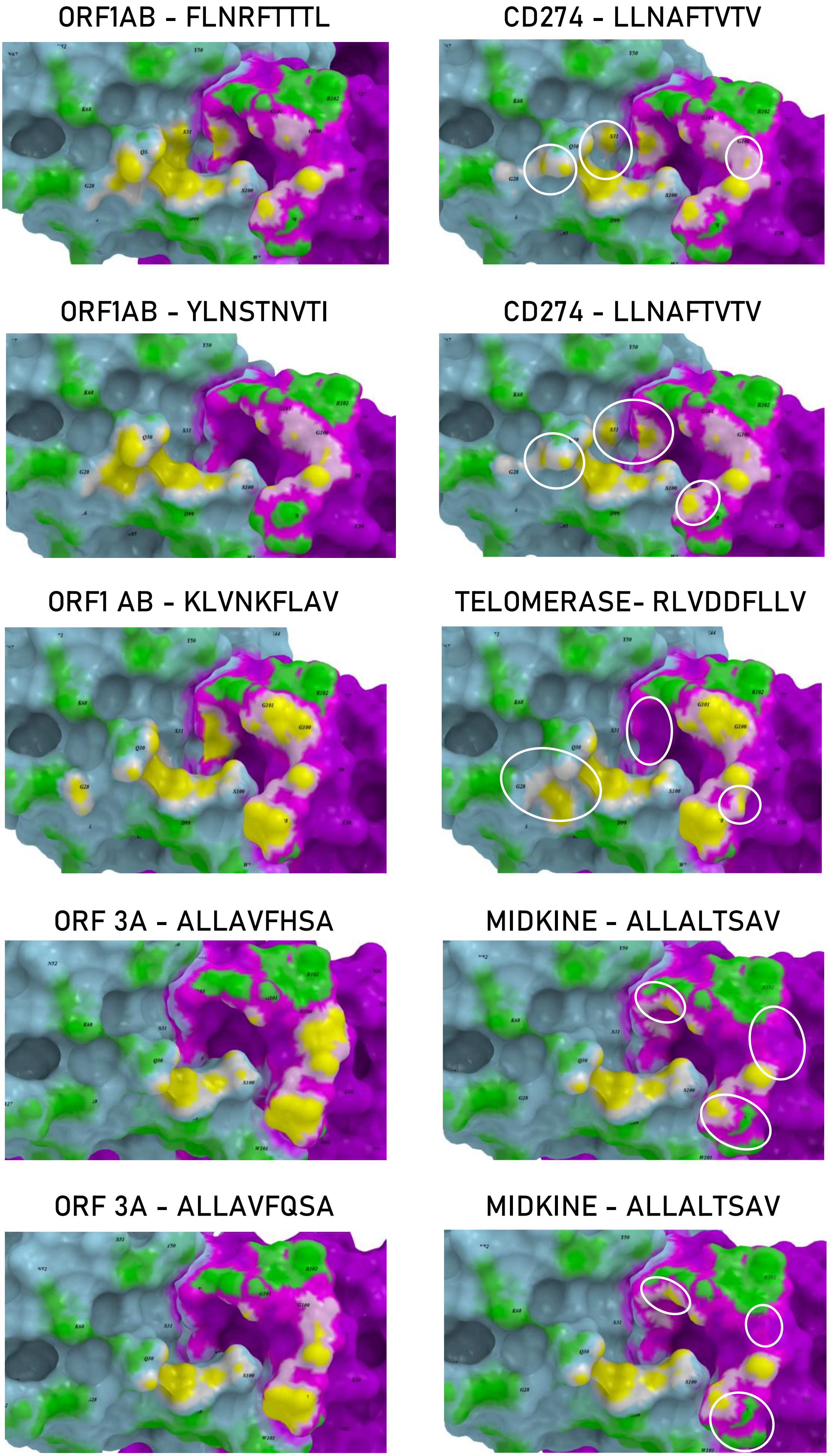
ORF1AB - FLNRFTTTL
CD274 - LLNAFTVTV
ORF1AB - YLNSTNVTI
CD274 - LLNAFTVTV
ORF1 AB - KLVNKFLAV
TELOMERASE- RLVDDFLLV
ORF 3A - ALLAVFHSA
MIDKINE - ALLALTSAV
ORF 3A - ALLAVFQSA
MIDKINE - ALLALTSAV

Suppl. Fig. 34

ORF 1AB- ALLSDLQDL

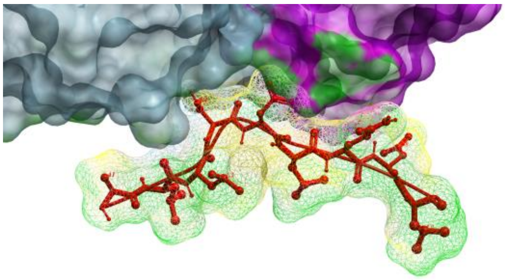

PRDX5- LLLDDLLVS

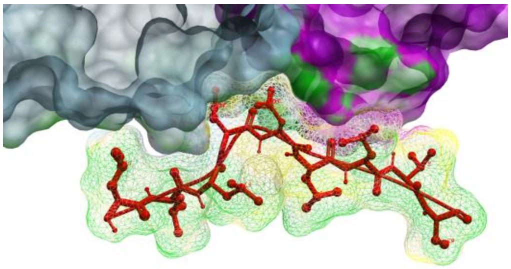

ORF 1AB- VLLAPLLSA

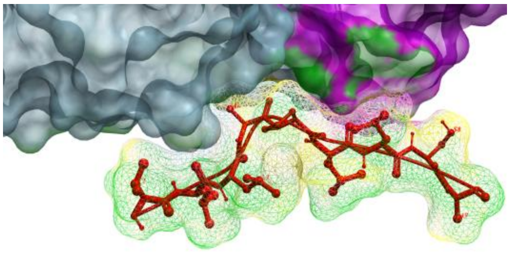

PRDX5- LLLDDLLVS

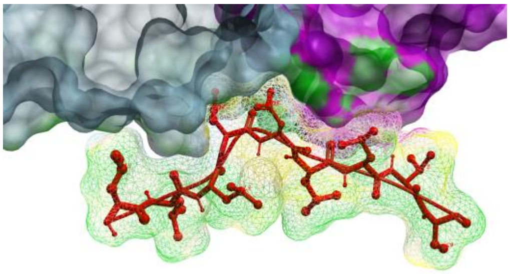

ORF 1AB- SLLSVLLSM

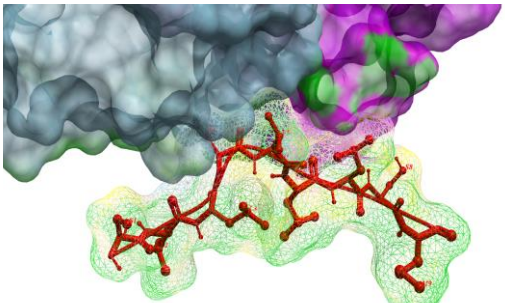

PRDX5- LLLDDLLVS

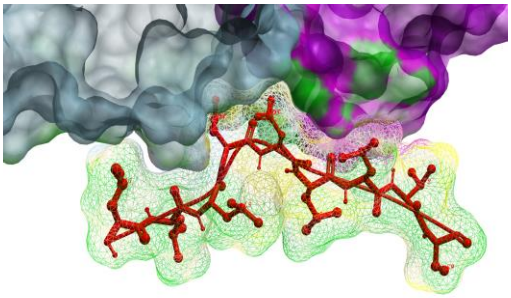

NUCLEOC- LLLDRLNQL

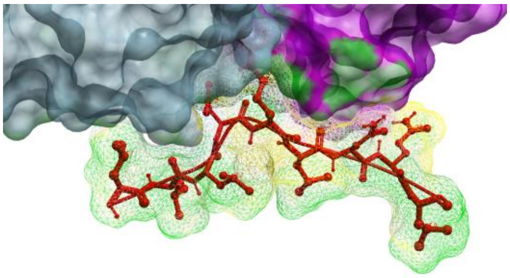

PRDX5- LLLDDLLVS

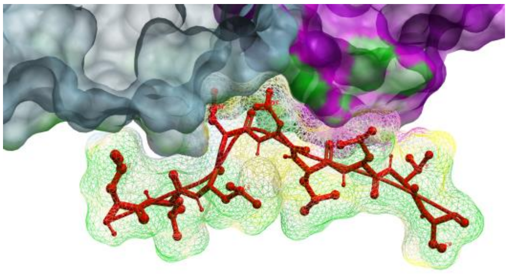

NUCLEOC -LLLDRLNQL

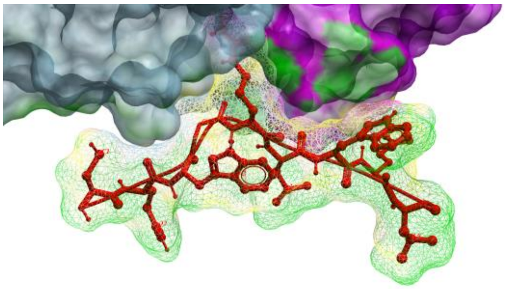

BING 4- CQWGRLWQL

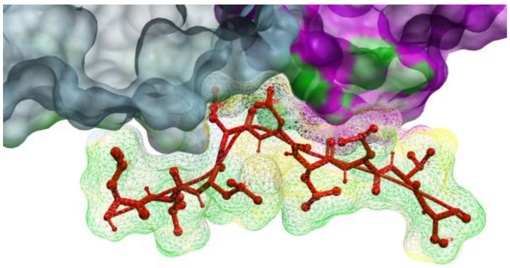

Suppl. Fig. 35

ORF 1AB- ALLSDLQDL

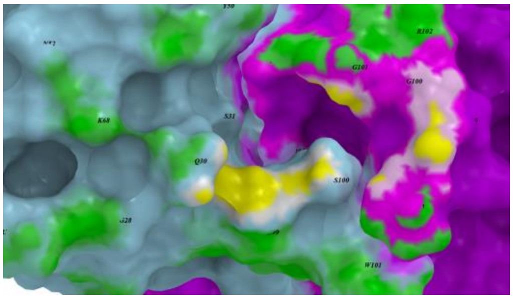

PRDX5- LLLDDLLVS

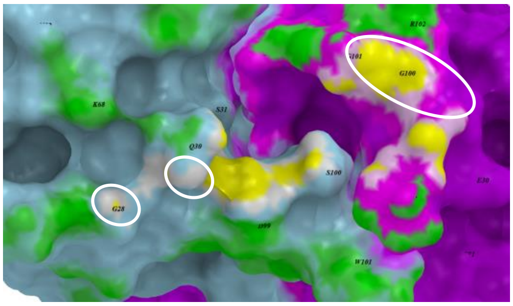

ORF 1AB- VLLAPLLSA

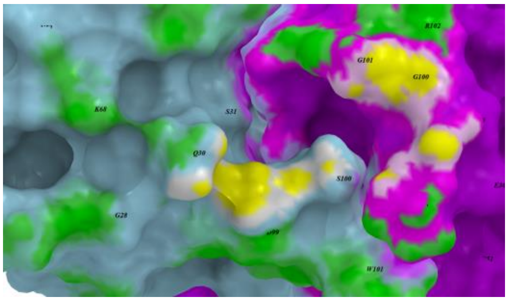

PRDX5- LLLDDLLVS

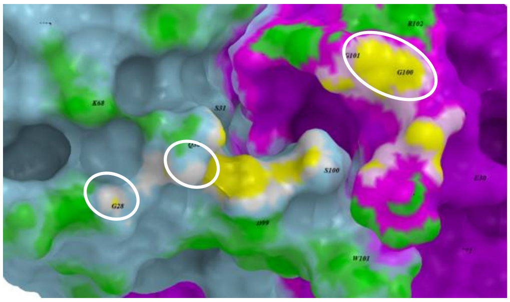

ORF 1AB- SLLSVLLSM

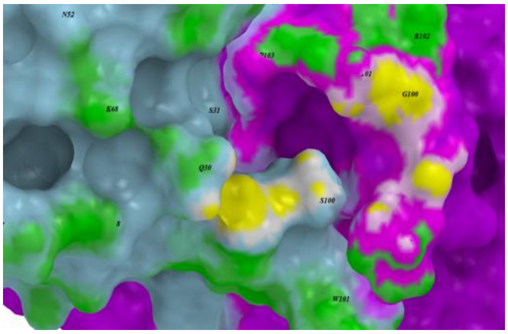

PRDX5- LLLDDLLVS

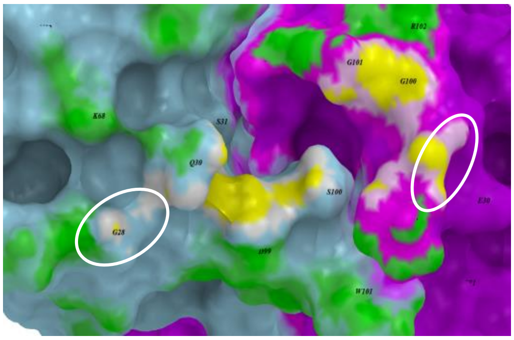

NUCLEOC- LLLDRLNQL

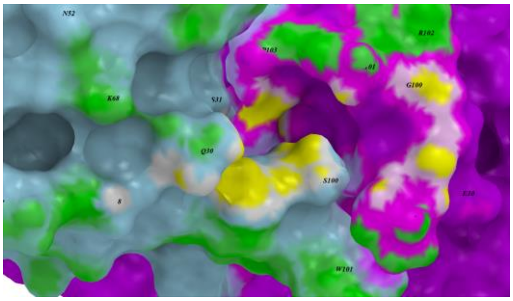

PRDX5- LLLDDLLVS

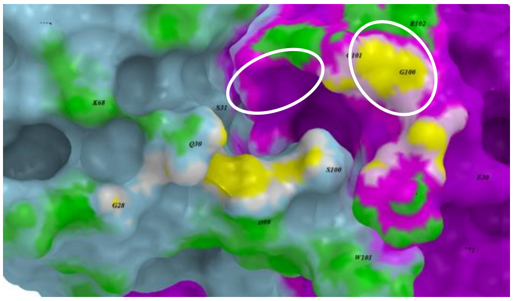

NUCLEOC -LLLDRLNQL

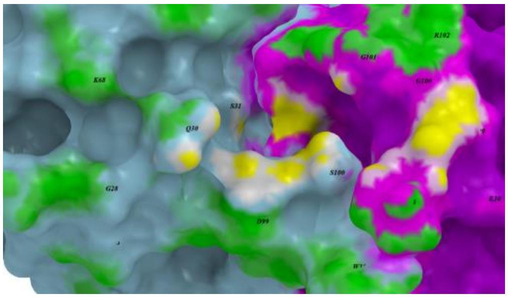

BING 4- CQWGRLWQL

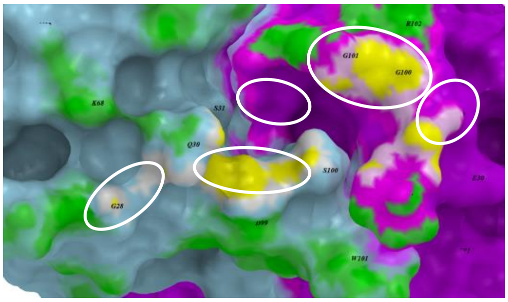

Suppl. Fig. 36

ORF 1AB-KLNEEIAII

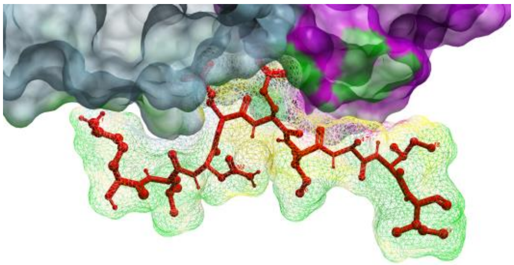

HAUS3- ILNAMIAKI

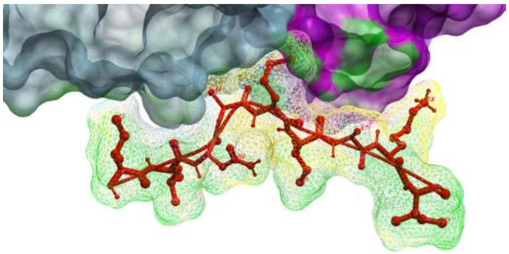

ORF 1AB- ILLLDQALV

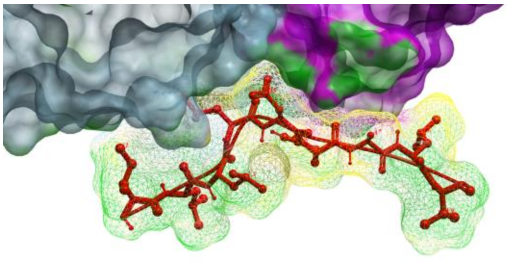

HER-2 -RLLQETELV

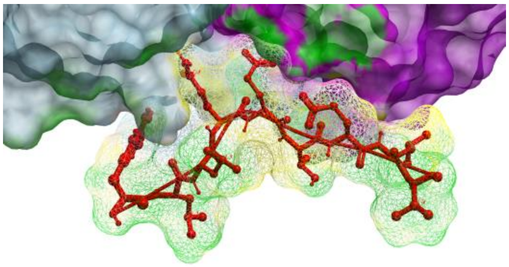

MEMB GLYCO- KLLEQWNLV

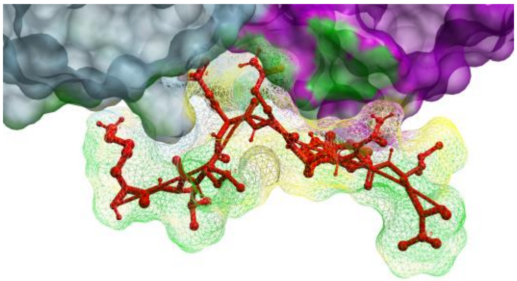

HER-2 -RLLQETELV

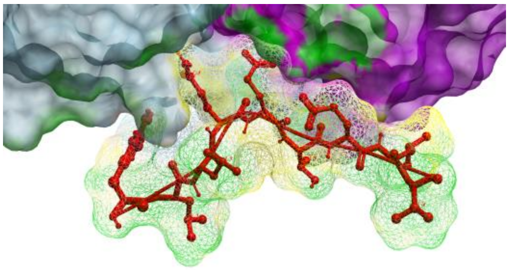

ORF1AB - ALLADKFPV

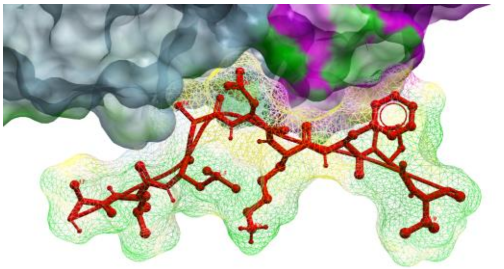

MDK - ALLALTSAV

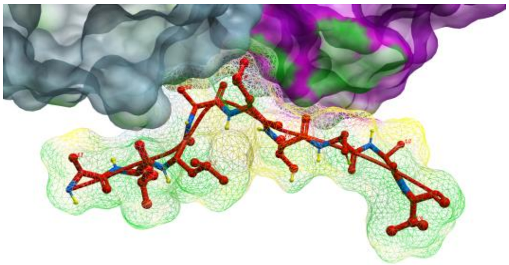

ORF 1AB - YLNTLTLAV

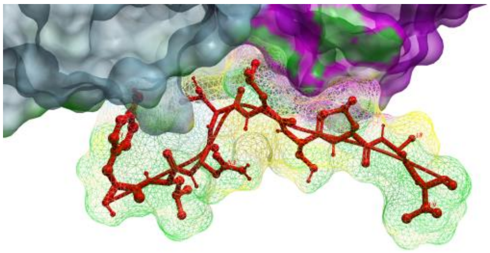

MDK - ALLALTSAV

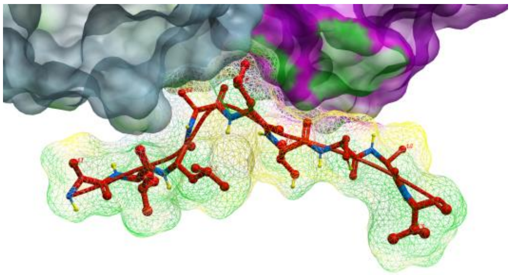

Suppl. Fig. 37

ORF 1AB-KLNEEIAII

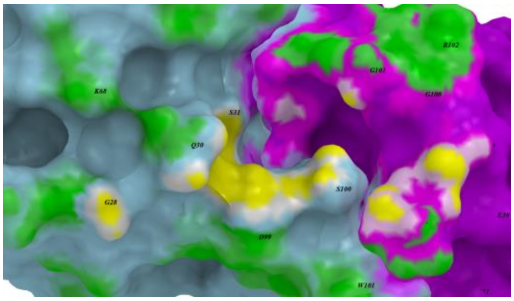

HAUS3- ILNAMIAKI

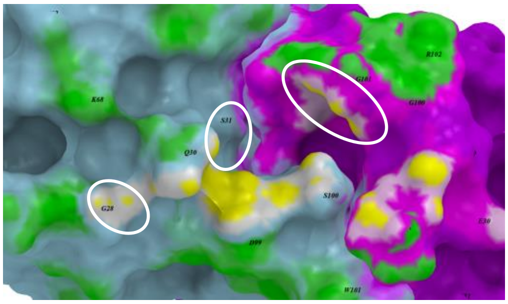

ORF 1AB- ILLLDQALV

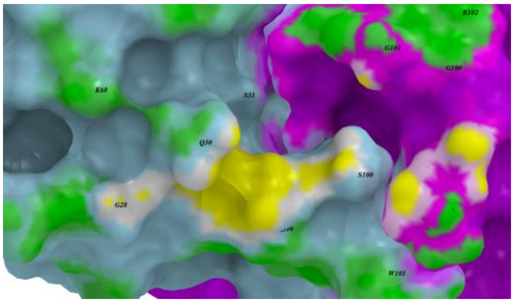

HER-2 -RLLQETELV

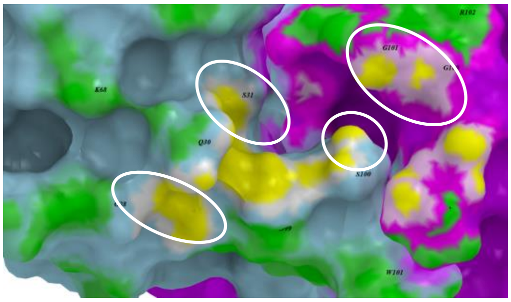

MEMB GLYCO- KLLEQWNLV

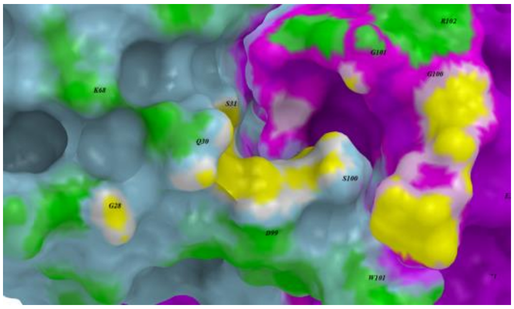

HER-2 -RLLQETELV

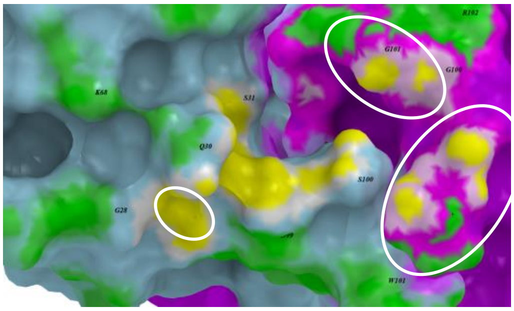

ORF1AB - ALLADKFPV

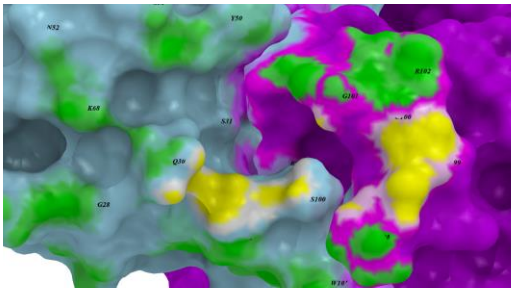

MDK - ALLALTSAV

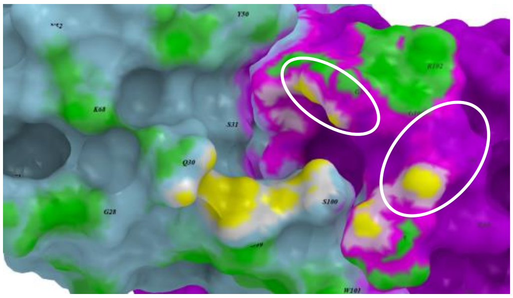

ORF 1AB - YLNTLTLAV

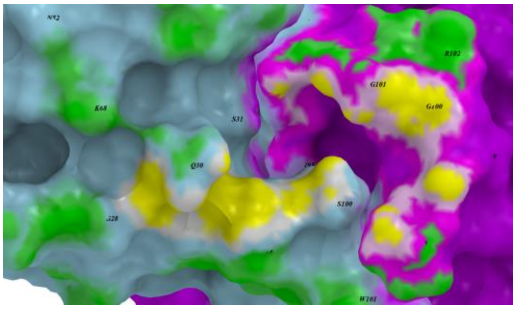

MDK - ALLALTSAV

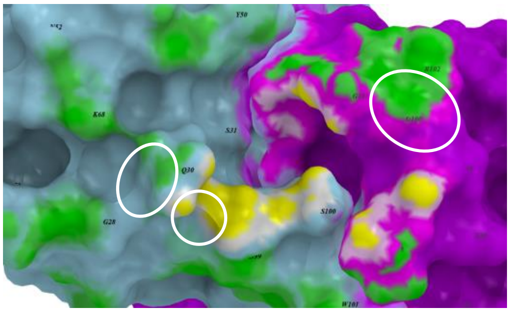

Suppl. Fig. 38

ORF 1AB - SLLMPILTL

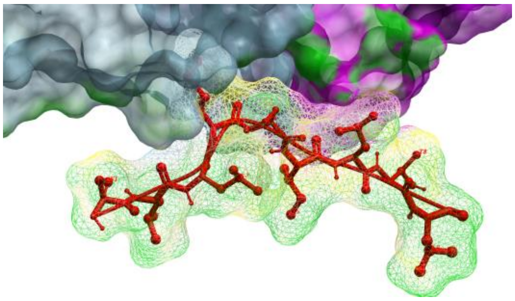

MUC1 - LLLLTVLTV

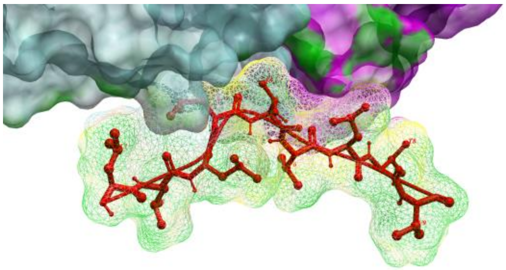

ORF 1AB - LLFLMSFTV

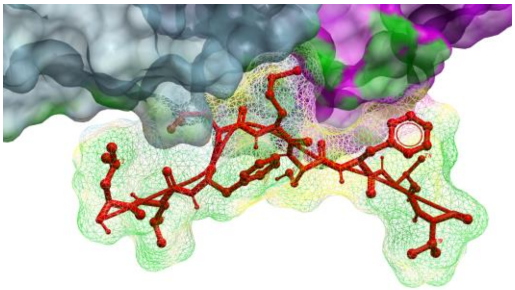

MUC1 - LLLLTVLTV

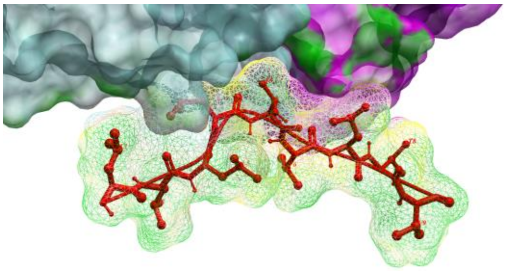

ORF 1AB- SLPGVFCGV

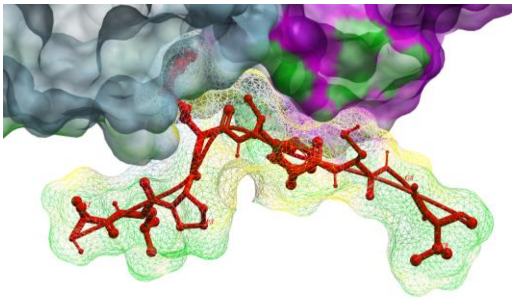

GNTV-VLPDVFIRV

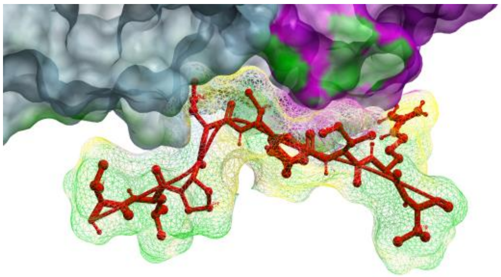

ORF 1AB- FLPRVFSAV

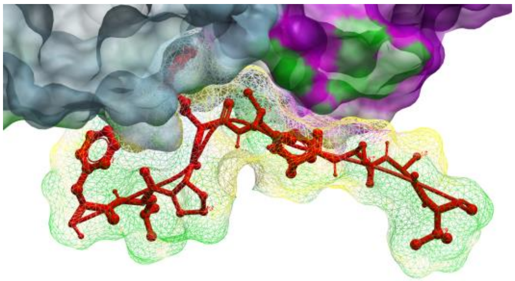

GNTV-VLPDVFIRV

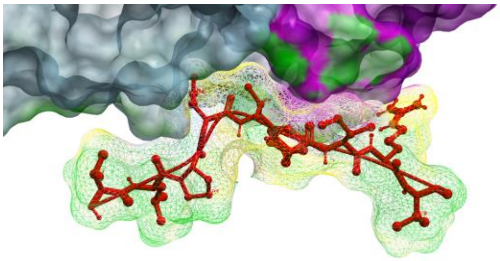

ORF 1 AB- LLLDDFVEI

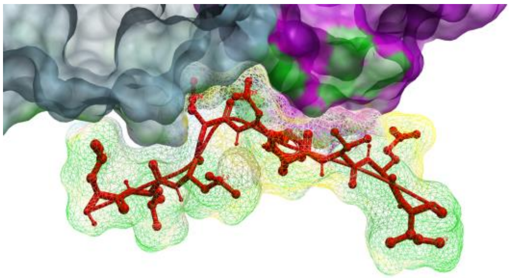

KIF20A- LLSDDDVVV

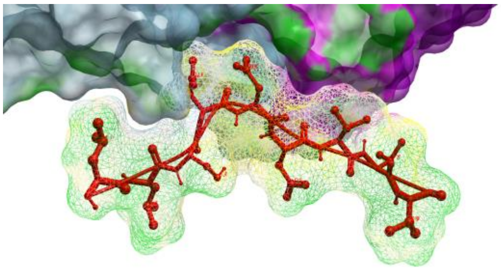

Suppl. Fig. 39

ORF 1AB - SLLMPILTL

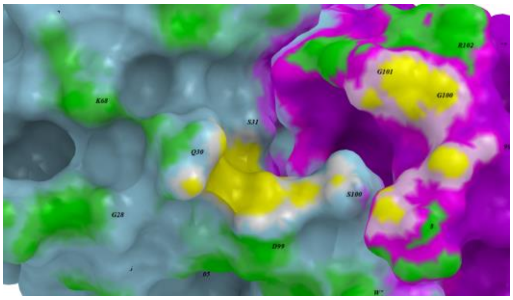

MUC1 - LLLLTVLTV

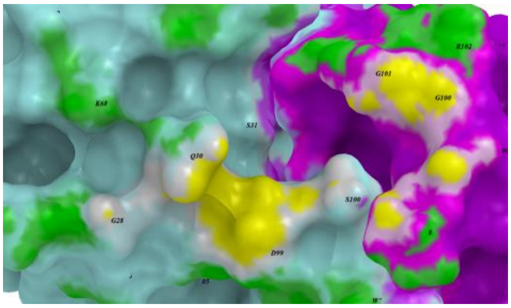

ORF 1AB - LLFLMSFTV

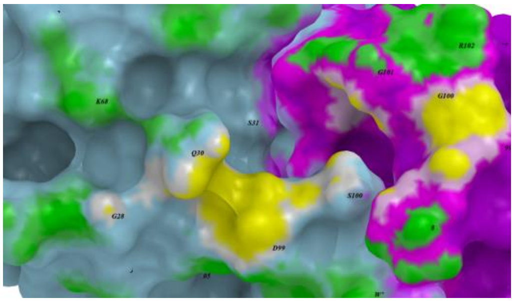

MUC1 - LLLLTVLTV

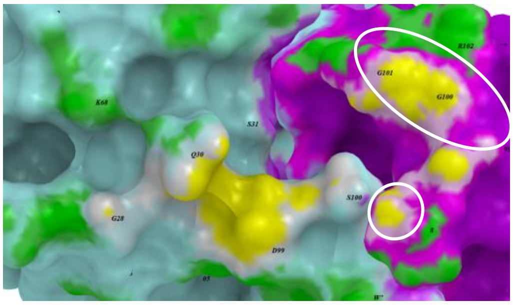

ORF 1AB- SLPGVFCGV

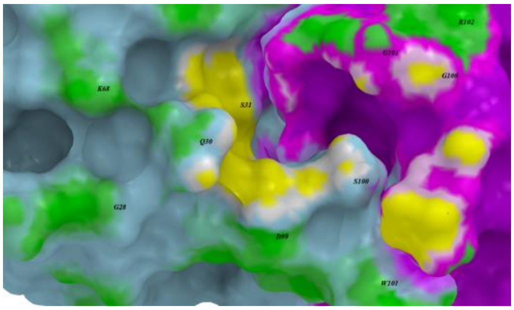

GNTV-VLPDVFIRV

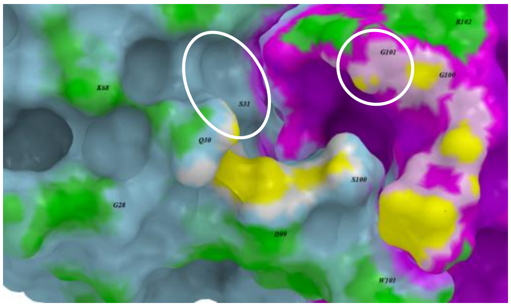

ORF 1AB- FLPRVFSAV

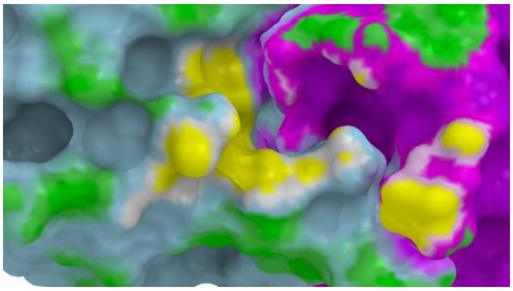

GNTV-VLPDVFIRV

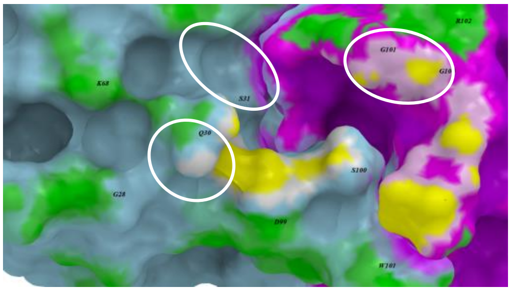

ORF 1 AB- LLLDDFVEI

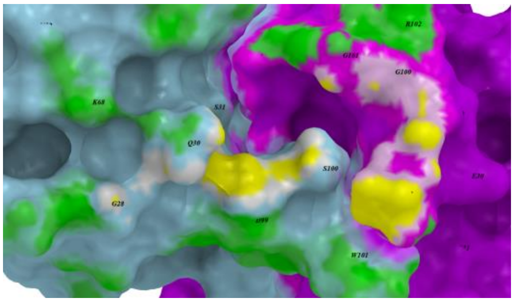

KIF20A- LLSDDDVVV

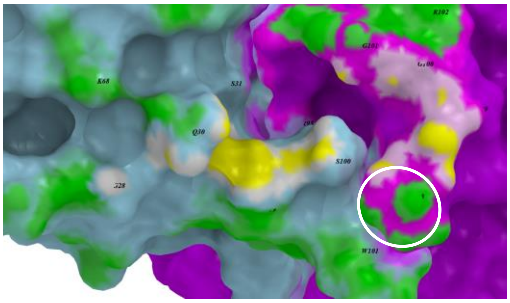

Suppl. Fig. 40

ORF 1 AB- ALWEIQQVV

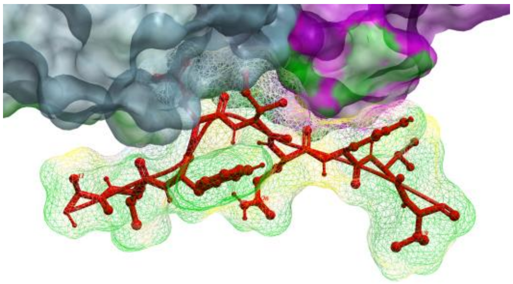

ID01- ALLEIASCL

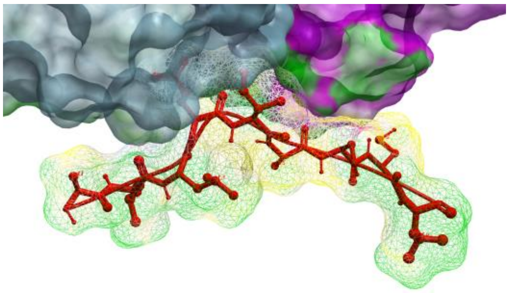

ORF 1AB - TLNDLNETL

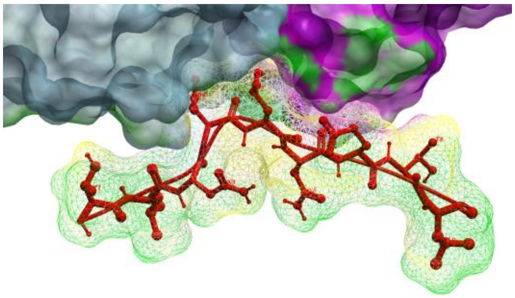

MELOE- TLNDECWPA

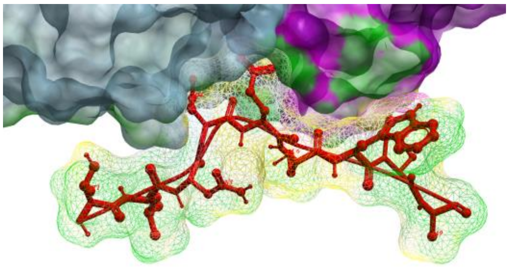

ORF 1AB- VLLAPLLSA

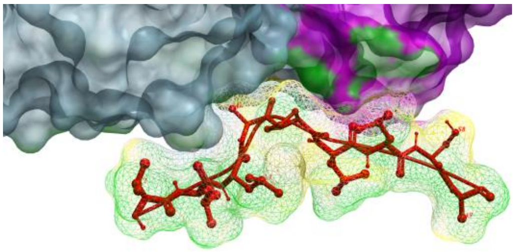

Nectin- 4 - VLVPPLPSL

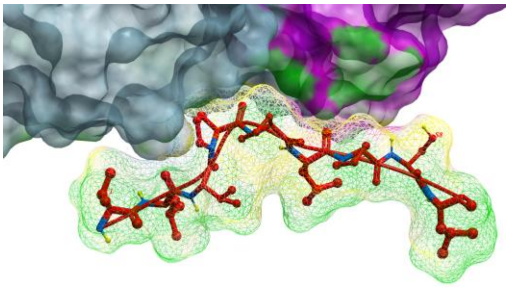

ORF 1AB- NVLTLVYKV

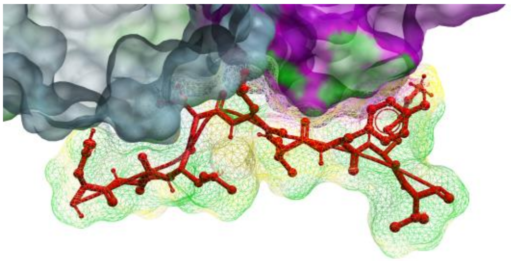

MAGE A1- KVLWYVIKV

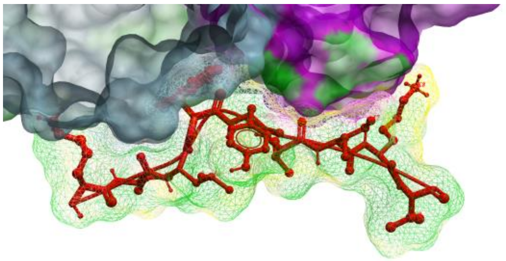

ORF 1AB- GVFCGVDAV

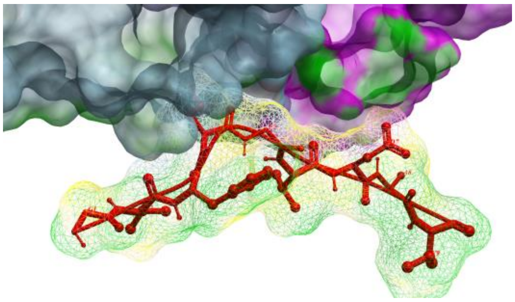

HEPSIN- GLQLGVQAV

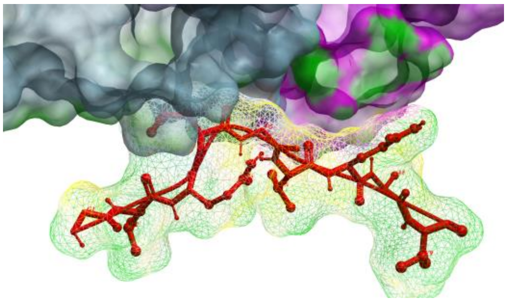

Suppl. Fig. 41

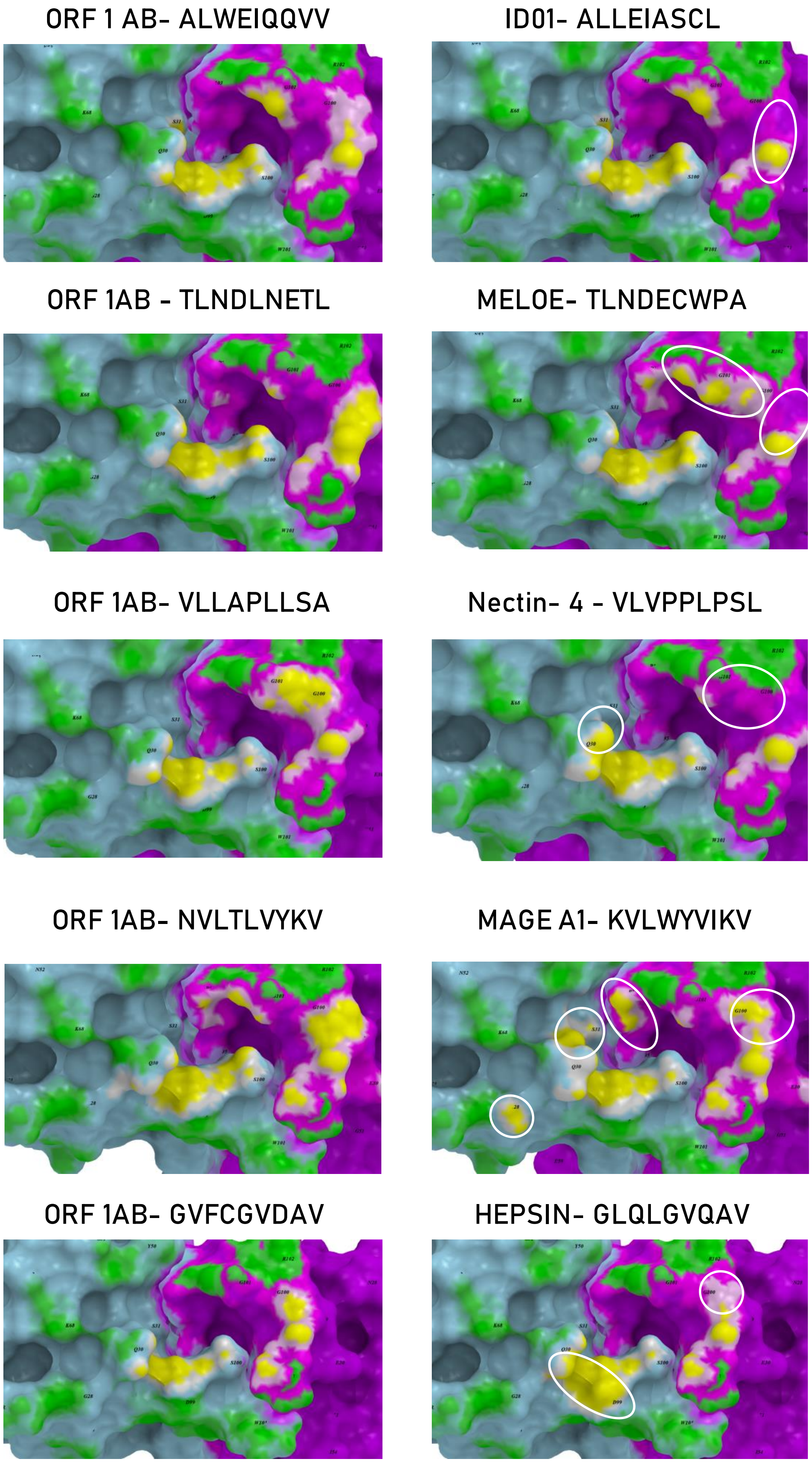
ORF 1 AB- ALWEIQQVV
ID01- ALLEIASCL
ORF 1AB - TLNDLNETL
MELOE- TLNDECWPA
ORF 1AB- VLLAPLLSA
Nectin- 4 - VLVPPLPSL
ORF 1AB- NVLTLVYKV
MAGE A1- KVLWYVIKV
ORF 1AB- GVFCGVDAV
HEPSIN- GLQLGVQAV

Suppl. Fig. 42

MEMB GLYCO - TLACFVLAA

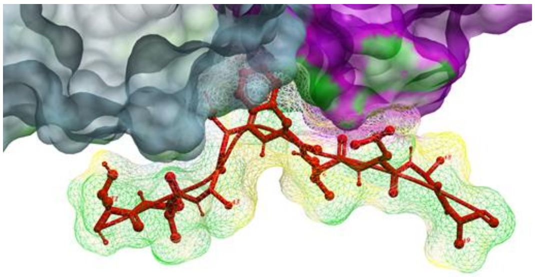

HEPCAM- RLAPFVYLL

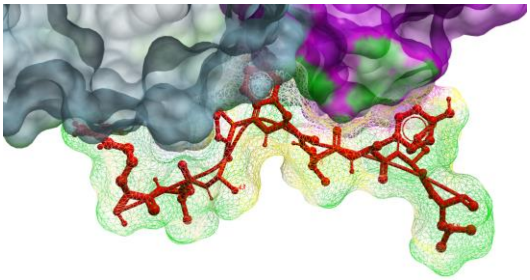

ORF7 -FLALITLAT

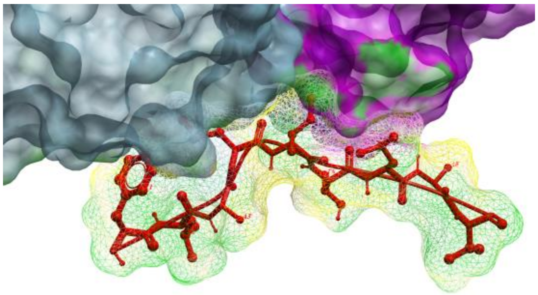

CALCA -FLALSILVL

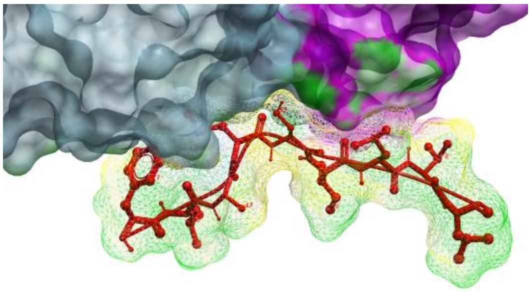

Suppl. Fig. 43

MEMB GLYCO - TLACFVLAA

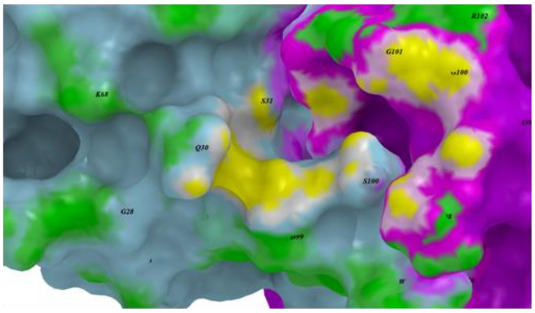

HEPCAM- RLAPFVYLL

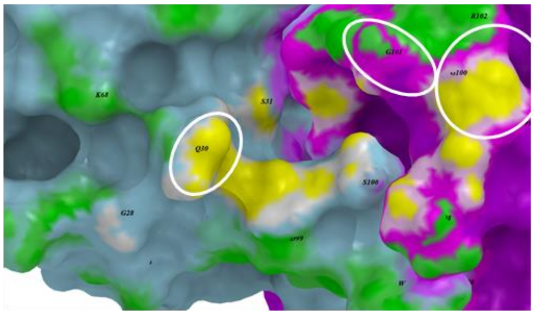

ORF7 -FLALITLAT

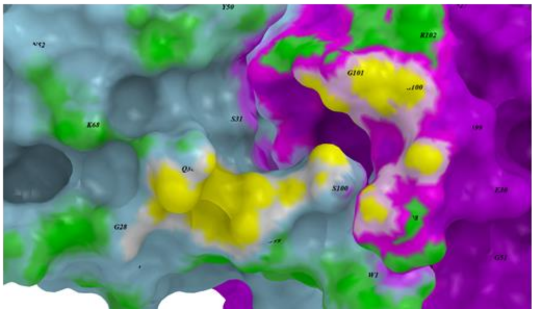

CALCA -FLALSILVL

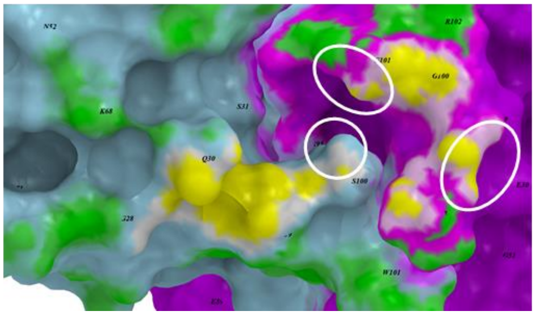

Suppl. Fig. 44

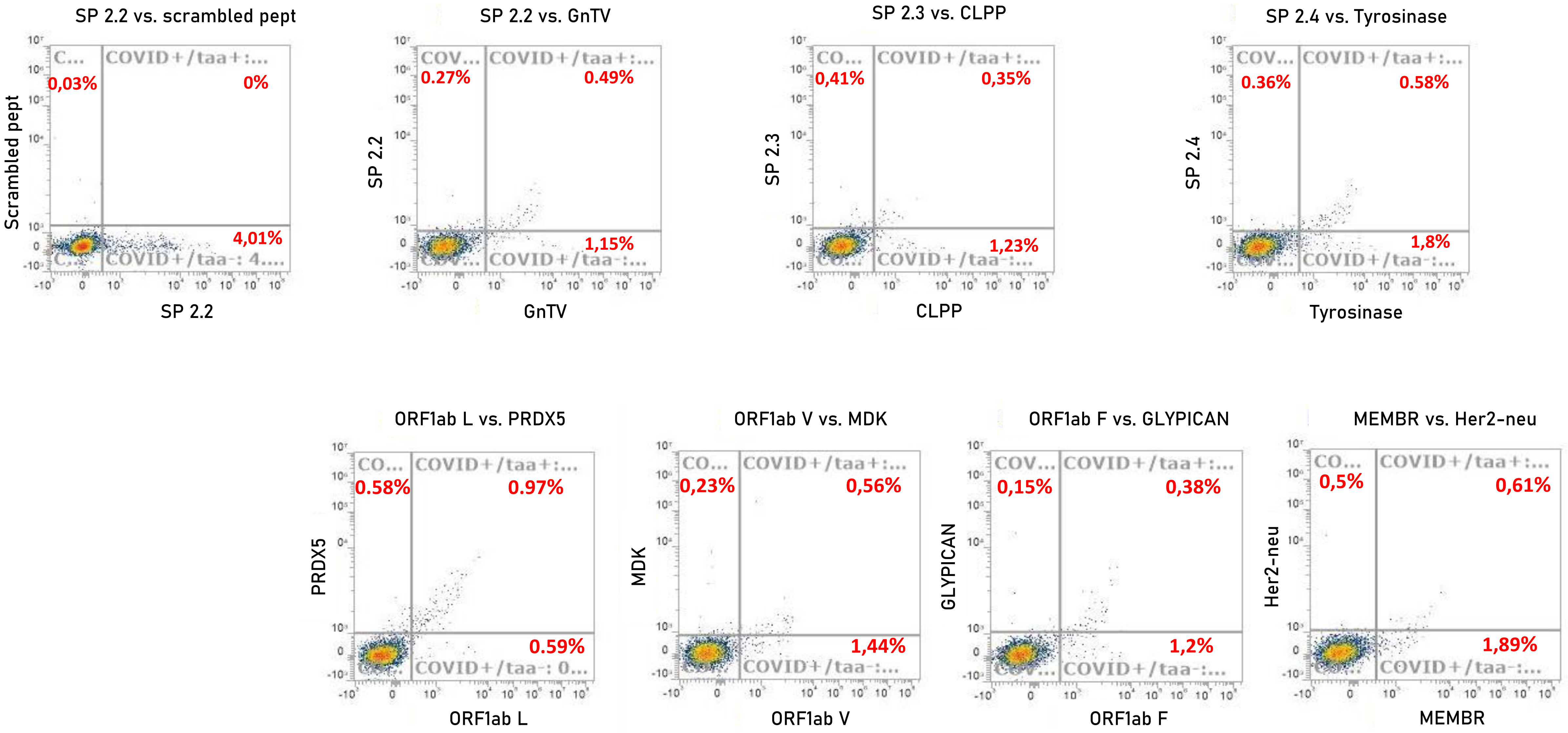
SP 2.2 vs. scrambled pept
Scrambled pept
SP 2.2
0,03%
0%
4,01%
SP 2.2 vs. GnTV
SP 2.2
GnTV
0.27%
0.49%
1,15%
SP 2.3 vs. CLPP
SP 2.3
CLPP
0,41%
0,35%
1,23%
SP 2.4 vs. Tyrosinase
SP 2.4
Tyrosinase
0.36%
0.58%
1,8%
ORF1ab L vs. PRDX5
PRDX5
ORF1ab L
0.58%
0.97%
0.59%
ORF1ab V vs. MDK
MDK
ORF1ab V
0,23%
0,56%
1,44%
ORF1ab F vs. GLYPICAN
GLYPICAN
ORF1ab F
0,15%
0,38%
1,2%
MEMBR vs. Her2-neu
Her2-neu
MEMBR
0,5%
0,61%
1,89%
COVID+/taa+:...
COVID+/taa-:...
