# Supplementary material for: Molecular mimicry of SARS-COV-2 antigens as a possible natural anti-cancer preventive immunization
Source: Front Immunol. 2024 Jun 14;15:1398002. doi: 10.3389/fimmu.2024.1398002 (PMC11211543; doi:10.3389/fimmu.2024.1398002)
Supplement: Supplementary file 2 [file Table_1.docx]

Suppl. Table 1 **List of 56 healthcare workers enrolled at the National Cancer Institute “Pascale” in Naples, ITALY and their HLA-A alleles.**

| **SAMPLE** | **HLA-A** |
| --- | --- |
| **1-AB** | 03:01/29:02 |
| **2-ASM** | 24:02/30:01 |
| **3-GS** | 01:01/30:01 |
| **4-FM** | 02:01/24:02 |
| **5-PP** | 02:01/25:01 |
| **6-FI** | 02:06/11:01 |
| **7-RDF** | 02:01/24:02 |
| **9-PM** | 01:01/30:01 |
| **10-PA** | 01:01/23:01P |
| **11-DAG** | 02:01/32:01 |
| **12-BV** | 03:01/24:02 |
| **13-GB** | 01:01/32:01 |
| **14-CM** | 30:01/32:01 |
| **15-CR** | 02:01/11:01 |
| **16-LB** | 02:01/26:01 |
| **17-FMB** | 02:01/26:01 |
| **18-ALT** | 02:01/03:01 |
| **19-MLT** | 02:01/32:01 |
| **20-GB** | 24:02/68:01 |
| **21-AI** | 01:01/02:01 |
| **22-SR** | 02:01/31:01 |
| **23-LM** | 02:01/69:01 |
| **24-DR** | 02:01/11:01 |
| **25-CE** | 02:01/11:01 |
| **27-ACP** | 01:01/69:01 |
| **28-LSS** | 01:01/29:02 |
| **29-IA** | 11:01/30:01 |
| **30-NS** | 02:01/26:01 |
| **SAMPLE** | **HLA-A** |
| **32-MT** | 03:01/24:02 |
| **34-RM** | 26:01/29:01 |
| **35-IC** | 01:01/68:02 |
| **36-DDA** | 03:01/68:01 |
| **40-FR** | 02:01/29:01 |
| **41-AP** | 02:01/32:01 |
| **45-AM** | 32:01/68:01 |
| **46-CM** | 24:02/32:01 |
| **47-SA** | 02:01/24:02 |
| **48-PF** | 02:01/01:01 |
| **49-LD** | 03:02/32:01 |
| **50-AC** | 03:01/68:02 |
| **51-RDM** | 24:02/32:01 |
| **54-DPM** | 30:02/32:01 |
| **55-BC** | 02:05/30:01 |
| **58-MT** | 23:01P/32:01 |
| **59-GA** | 01:01/24:02 |
| **60-ES** | 24:02/69:01 |
| **61-LDA** | 23:01P/29:01 |
| **62-RP** | 02:01 |
| **64-VDD** | 02:01/03:01 |
| **65-SM** | 02:01/25:01 |
| **66-AB** | 01:01/32:01 |
| **67-FMT** | 02:01 |
| **69-CM** | 03:01/68:01 |
| **70-CDN** | 02:01/33:01 |
| **71-PB** | 24:02/32:02 |
| **72-SM** | 02:01/03:02 |

Suppl. Table 2 **Sequence alignment of the spike proteins from the VOCs.** The spike proteins from all the major VOCs (<https://viralzone.expasy.org/>) were aligned. Chemical properties are indicated by color code. Asterisk (*) indicates identity between the residues in all proteins.

Wuhan MFVFLVLLPLVSSQCVNLTTRTQLPPAYTNSFTRGVYYPDKVFRSSVLHSTQDLFLPFFSNVTWFHAIHVSGTNGTKRFDNPVLPFNDGVYFASTEKSNI

Alfa MFVFLVLLPLVSSQCVNLTTRTQLPPAYTNSFTRGVYYPDKVFRSSVLHSTQDLFLPFFSNVTWFHAI--SGTNGTKRFDNPVLPFNDGVYFASTEKSNI

Beta MFVFLVLLPLVSSQCVNLTTRTQLPPAYTNSFTRGVYYPDKVFRSSVLHSTQDLFLPFFSNVTWFHAIHVSGTNGTKRFANPVLPFNDGVYFASTEKSNI

Gamma MFVFLVLLPLVSSQCVNFTNRTQLPSAYTNSFTRGVYYPDKVFRSSVLHSTQDLFLPFFSNVTWFHAIHVSGTNGTKRFDNPVLPFNDGVYFASTEKSNI

Delta MFVFLVLLPLVSSQCVNLRTRTQLPPAYTNSFTRGVYYPDKVFRSSVLHSTQDLFLPFFSNVTWFHAIHVSGTNGTTRFDNPVLPFNDGVYFASTEKSNI

Omicron MFVFLVLLPLVSSQCVNLTTRTQLPPAYTNSFTRGVYYPDKVFRSSVLHSTQDLFLPFFSNVTWFHVI--SGTNGTKRFDNPVLPFNDGVYFASIEKSNI

***************** ***** **************************************** * ****** ** ************** *****

Wuhan IRGWIFGTTLDSKTQSLLIVNNATNVVIKVCEFQFCNDPFLGVYYHKNNKSWMESEFRVYSSANNCTFEYVSQPFLMDLEGKQGNFKNLREFVFKNIDGY

Alfa IRGWIFGTTLDSKTQSLLIVNNATNVVIKVCEFQFCNDPFLGV-YHKNNKSWMESEFRVYSSANNCTFEYVSQPFLMDLEGKQGNFKNLREFVFKNIDGY

Beta IRGWIFGTTLDSKTQSLLIVNNATNVVIKVCEFQFCNDPFLGVYYHKNNKSWMESEFRVYSSANNCTFEYVSQPFLMDLEGKQGNFKNLREFVFKNIDGY

Gamma IRGWIFGTTLDSKTQSLLIVNNATNVVIKVCEFQFCNYPFLGVYYHKNNKSWMESEFRVYSSANNCTFEYVSQPFLMDLEGKQGNFKNLSEFVFKNIDGY

Delta IRGWIFGTTLDSKTQSLLIVNNATNVVIKVCEFQFCNDPFLGVYYHKNNKSWMES--GVYSSANNCTFEYVSQPFLMDLEGKQGNFKNLREFVFKNIDGY

Omicron IRGWIFGTTLDSKTQSLLIVNNATNVVIKVCEFQFCNDPFLD---HKNNKSWMESEFRVYSSANNCTFEYVSQPFLMDLEGKQGNFKNLREFVFKNIDGY

************************************* *** ********** ******************************* **********

Wuhan FKIYSKHTPINL--VRDLPQGFSALEPLVDLPIGINITRFQTLLALHRSYLTPGDSSSGWTAGAAAYYVGYLQPRTFLLKYNENGTITDAVDCALDPLSE

Alfa FKIYSKHTPINL--VRDLPQGFSALEPLVDLPIGINITRFQTLLALHRSYLTPGDSSSGWTAGAAAYYVGYLQPRTFLLKYNENGTITDAVDCALDPLSE

Beta FKIYSKHTPINL--VRGLPQGFSALEPLVDLPIGINITRFQT---LHRSYLTPGDSSSGWTAGAAAYYVGYLQPRTFLLKYNENGTITDAVDCALDPLSE

Gamma FKIYSKHTPINL--VRDLPQGFSALEPLVDLPIGINITRFQTLLALHRSYLTPGDSSSGWTAGAAAYYVGYLQPRTFLLKYNENGTITDAVDCALDPLSE

Delta FKIYSKHTPINL--VRDLPQGFSALEPLVDLPIGINITRFQTLLALHRSYLTPGDSSSGWTAGAAAYYVGYLQPRTFLLKYNENGTITDAVDCALDPLSE

Omicron FKIYSKHTPIIVREPEDLPQGFSALEPLVDLPIGINITRFQTLLALHRSYLTPGDSSSGWTAGAAAYYVGYLQPRTFLLKYNENGTITDAVDCALDPLSE

********** ************************* *******************************************************

Wuhan TKCTLKSFTVEKGIYQTSNFRVQPTESIVRFPNITNLCPFGEVFNATRFASVYAWNRKRISNCVADYSVLYNSASFSTFKCYGVSPTKLNDLCFTNVYAD

Alfa TKCTLKSFTVEKGIYQTSNFRVQPTESIVRFPNITNLCPFGEVFNATRFASVYAWNRKRISNCVADYSVLYNSASFSTFKCYGVSPTKLNDLCFTNVYAD

Beta TKCTLKSFTVEKGIYQTSNFRVQPTESIVRFPNITNLCPFGEVFNATRFASVYAWNRKRISNCVADYSVLYNSASFSTFKCYGVSPTKLNDLCFTNVYAD

Gamma TKCTLKSFTVEKGIYQTSNFRVQPTESIVRFPNITNLCPFGEVFNATRFASVYAWNRKRISNCVADYSVLYNSASFSTFKCYGVSPTKLNDLCFTNVYAD

Delta TKCTLKSFTVEKGIYQTSNFRVQPTESIVRFPNITNLCPFGEVFNATRFASVYAWNRKRISNCVADYSVLYNSASFSTFKCYGVSPTKLNDLCFTNVYAD

Omicron TKCTLKSFTVEKGIYQTSNFRVQPTESIVRFPNITNLCPFDEVFNATRFASVYAWNRKRISNCVADYSVLYNLAPFFTFKCYGVSPTKLNDLCFTNVYAD

**************************************** ******************************* * * ***********************

Wuhan SFVIRGDEVRQIAPGQTGKIADYNYKLPDDFTGCVIAWNSNNLDSKVGGNYNYLYRLFRKSNLKPFERDISTEIYQAGSTPCNGVEGFNCYFPLQSYGFQ

Alfa SFVIRGDEVRQIAPGQTGKIADYNYKLPDDFTGCVIAWNSNNLDSKVGGNYNYLYRLFRKSNLKPFERDISTEIYQAGSTPCNGVEGFNCYFPLQSYGFQ

Beta SFVIRGDEVRQIAPGQTGNIADYNYKLPDDFTGCVIAWNSNNLDSKVGGNYNYLYRLFRKSNLKPFERDISTEIYQAGSTPCNGVKGFNCYFPLQSYGFQ

Gamma SFVIRGDEVRQIAPGQTGTIADYNYKLPDDFTGCVIAWNSNNLDSKVGGNYNYLYRLFRKSNLKPFERDISTEIYQAGSTPCNGVKGFNCYFPLQSYGFQ

Delta SFVIRGDEVRQIAPGQTGKIADYNYKLPDDFTGCVIAWNSNNLDSKVGGNYNYRYRLFRKSNLKPFERDISTEIYQAGSKPCNGVEGFNCYFPLQSYGFQ

Omicron SFVIRGDEVRQIAPGQTGNIADYNYKLPDDFTGCVIAWNSNKLDSKVSGNYNYLYRLFRKSNLKPFERDISTEIYQAGNKPCNGVAGFNCYFPLRSYSFR

******************.**********************:*****.***** ************************..***** ********:**.*:

Wuhan PTNGVGYQPYRVVVLSFELLHAPATVCGPKKSTNLVKNKCVNFNFNGLTGTGVLTESNKKFLPFQQFGRDIADTTDAVRDPQTLEILDITPCSFGGVSVI

Alfa PTYGVGYQPYRVVVLSFELLHAPATVCGPKKSTNLVKNKCVNFNFNGLTGTGVLTESNKKFLPFQQFGRDIDDTTDAVRDPQTLEILDITPCSFGGVSVI

Beta PTYGVGYQPYRVVVLSFELLHAPATVCGPKKSTNLVKNKCVNFNFNGLTGTGVLTESNKKFLPFQQFGRDIADTTDAVRDPQTLEILDITPCSFGGVSVI

Gamma PTYGVGYQPYRVVVLSFELLHAPATVCGPKKSTNLVKNKCVNFNFNGLTGTGVLTESNKKFLPFQQFGRDIADTTDAVRDPQTLEILDITPCSFGGVSVI

Delta PTNGVGYQPYRVVVLSFELLHAPATVCGPKKSTNLVKNKCVNFNFNGLTGTGVLTESNKKFLPFQQFGRDIADTTDAVRDPQTLEILDITPCSFGGVSVI

Omicron PTYGVGHQPYRVVVLSFELLHAPATVCGPKKSTNLVKNKCVNFNFNGLKGTGVLTESNKKFLPFQQFGRDIADTTDAVRDPQTLEILDITPCSFGGVSVI

** *** ***************************************** ********************** ****************************

Wuhan TPGTNTSNQVAVLYQDVNCTEVPVAIHADQLTPTWRVYSTGSNVFQTRAGCLIGAEHVNNSYECDIPIGAGICASYQTQTNSPRRARSVASQSIIAYTMS

Alfa TPGTNTSNQVAVLYQGVNCTEVPVAIHADQLTPTWRVYSTGSNVFQTRAGCLIGAEHVNNSYECDIPIGAGICASYQTQTNSHRRARSVASQSIIAYTMS

Beta TPGTNTSNQVAVLYQGVNCTEVPVAIHADQLTPTWRVYSTGSNVFQTRAGCLIGAEHVNNSYECDIPIGAGICASYQTQTNSPRRARSVASQSIIAYTMS

Gamma TPGTNTSNQVAVLYQGVNCTEVPVAIHADQLTPTWRVYSTGSNVFQTRAGCLIGAEYVNNSYECDIPIGAGICASYQTQTNSPRRARSVASQSIIAYTMS

Delta TPGTNTSNQVAVLYQGVNCTEVPVAIHADQLTPTWRVYSTGSNVFQTRAGCLIGAEHVNNSYECDIPIGAGICASYQTQTNSRRRARSVASQSIIAYTMS

Omicron TPGTNTSNQVAVLYQGVNCTEVPVAIHADQLTPTWRVYSTGSNVFQTRAGCLIGAEYVNNSYECDIPIGAGICASYQTQTKSHRRARSVASQSIIAYTMS

*************** **************************************** *********************** * *****************

Wuhan LGAENSVAYSNNSIAIPTNFTISVTTEILPVSMTKTSVDCTMYICGDSTECSNLLLQYGSFCTQLNRALTGIAVEQDKNTQEVFAQVKQIYKTPPIKDFG

Alfa LGAENSVAYSNNSIAIPINFTISVTTEILPVSMTKTSVDCTMYICGDSTECSNLLLQYGSFCTQLNRALTGIAVEQDKNTQEVFAQVKQIYKTPPIKDFG

Beta LGVENSVAYSNNSIAIPTNFTISVTTEILPVSMTKTSVDCTMYICGDSTECSNLLLQYGSFCTQLNRALTGIAVEQDKNTQEVFAQVKQIYKTPPIKDFG

Gamma LGAENSVAYSNNSIAIPTNFTISVTTEILPVSMTKTSVDCTMYICGDSTECSNLLLQYGSFCTQLNRALTGIAVEQDKNTQEVFAQVKQIYKTPPIKDFG

Delta LGAENSVAYSNNSIAIPTNFTISVTTEILPVSMTKTSVDCTMYICGDSTECSNLLLQYGSFCTQLNRALTGIAVEQDKNTQEVFAQVKQIYKTPPIKDFG

Omicron LGAENSVAYSNNSIAIPTNFTISVTTEILPVSMTKTSVDCTMYICGDSTECSNLLLQYGSFCTQLKRALTGIAVEQDKNTQEVFAQVKQIYKTPPIKYFG

** ************** *********************************************** ******************************* **

Wuhan GFNFSQILPDPSKPSKRSFIEDLLFNKVTLADAGFIKQYGDCLGDIAARDLICAQKFNGLTVLPPLLTDEMIAQYTSALLAGTITSGWTFGAGAALQIPF

Alfa GFNFSQILPDPSKPSKRSFIEDLLFNKVTLADAGFIKQYGDCLGDIAARDLICAQKFNGLTVLPPLLTDEMIAQYTSALLAGTITSGWTFGAGAALQIPF

Beta GFNFSQILPDPSKPSKRSFIEDLLFNKVTLADAGFIKQYGDCLGDIAARDLICAQKFNGLTVLPPLLTDEMIAQYTSALLAGTITSGWTFGAGAALQIPF

Gamma GFNFSQILPDPSKPSKRSFIEDLLFNKVTLADAGFIKQYGDCLGDIAARDLICAQKFNGLTVLPPLLTDEMIAQYTSALLAGTITSGWTFGAGAALQIPF

Delta GFNFSQILPDPSKPSKRSFIEDLLFNKVTLADAGFIKQYGDCLGDIAARDLICAQKFNGLTVLPPLLTDEMIAQYTSALLAGTITSGWTFGAGAALQIPF

Omicron GFNFSQILPDPSKPSKRSFIEDLLFNKVTLADAGFIKQYGDCLGDIAARDLICAQKFKGLTVLPPLLTDEMIAQYTSALLAGTITSGWTFGAGAALQIPF

********************************************************* ******************************************

Wuhan AMQMAYRFNGIGVTQNVLYENQKLIANQFNSAIGKIQDSLSSTASALGKLQDVVNQNAQALNTLVKQLSSNFGAISSVLNDILSRLDKVEAEVQIDRLIT

Alfa AMQMAYRFNGIGVTQNVLYENQKLIANQFNSAIGKIQDSLSSTASALGKLQDVVNQNAQALNTLVKQLSSNFGAISSVLNDILARLDKVEAEVQIDRLIT

Beta AMQMAYRFNGIGVTQNVLYENQKLIANQFNSAIGKIQDSLSSTASALGKLQDVVNQNAQALNTLVKQLSSNFGAISSVLNDILSRLDKVEAEVQIDRLIT

Gamma AMQMAYRFNGIGVTQNVLYENQKLIANQFNSAIGKIQDSLSSTASALGKLQDVVNQNAQALNTLVKQLSSNFGAISSVLNDILSRLDKVEAEVQIDRLIT

Delta AMQMAYRFNGIGVTQNVLYENQKLIANQFNSAIGKIQDSLSSTASALGKLQNVVNQNAQALNTLVKQLSSNFGAISSVLNDILSRLDKVEAEVQIDRLIT

Omicron AMQMAYRFNGIGVTQNVLYENQKLIANQFNSAIGKIQDSLSSTASALGKLQDVVNHNAQALNTLVKQLSSKFGAISSVLNDIFSRLDKVEAEVQIDRLIT

*************************************************** *** ************** *********** ****************

Wuhan GRLQSLQTYVTQQLIRAAEIRASANLAATKMSECVLGQSKRVDFCGKGYHLMSFPQSAPHGVVFLHVTYVPAQEKNFTTAPAICHDGKAHFPREGVFVSN

Alfa GRLQSLQTYVTQQLIRAAEIRASANLAATKMSECVLGQSKRVDFCGKGYHLMSFPQSAPHGVVFLHVTYVPAQEKNFTTAPAICHDGKAHFPREGVFVSN

Beta GRLQSLQTYVTQQLIRAAEIRASANLAATKMSECVLGQSKRVDFCGKGYHLMSFPQSAPHGVVFLHVTYVPAQEKNFTTAPAICHDGKAHFPREGVFVSN

Gamma GRLQSLQTYVTQQLIRAAEIRASANLAAIKMSECVLGQSKRVDFCGKGYHLMSFPQSAPHGVVFLHVTYVPAQEKNFTTAPAICHDGKAHFPREGVFVSN

Delta GRLQSLQTYVTQQLIRAAEIRASANLAATKMSECVLGQSKRVDFCGKGYHLMSFPQSAPHGVVFLHVTYVPAQEKNFTTAPAICHDGKAHFPREGVFVSN

Omicron GRLQSLQTYVTQQLIRAAEIRASANLAATKMSECVLGQSKRVDFCGKGYHLMSFPQSAPHGVVFLHVTYVPAQEKNFTTAPAICHDGKAHFPREGVFVSN

**************************** ***********************************************************************

Wuhan GTHWFVTQRNFYEPQIITTDNTFVSGNCDVVIGIVNNTVYDPLQPELDSFKEELDKYFKNHTSPDVDLGDISGINASVVNIQKEIDRLNEVAKNLNESLI

Alfa GTHWFVTQRNFYEPQIITTHNTFVSGNCDVVIGIVNNTVYDPLQPELDSFKEELDKYFKNHTSPDVDLGDISGINASVVNIQKEIDRLNEVAKNLNESLI

Beta GTHWFVTQRNFYEPQIITTDNTFVSGNCDVVIGIVNNTVYDPLQPELDSFKEELDKYFKNHTSPDVDLGDISGINASVVNIQKEIDRLNEVAKNLNESLI

Gamma GTHWFVTQRNFYEPQIITTDNTFVSGNCDVVIGIVNNTVYDPLQPELDSFKEELDKYFKNHTSPDVDLGDISGINASFVNIQKEIDRLNEVAKNLNESLI

Delta GTHWFVTQRNFYEPQIITTDNTFVSGNCDVVIGIVNNTVYDPLQPELDSFKEELDKYFKNHTSPDVDLGDISGINASVVNIQKEIDRLNEVAKNLNESLI

Omicron GTHWFVTQRNFYEPQIITTDNTFVSGNCDVVIGIVNNTVYDPLQPELDSFKEELDKYFKNHTSPDVDLGDISGINASVVNIQKEIDRLNEVAKNLNESLI

******************* ********************************************************* **********************

Wuhan DLQELGKYEQYIKWPWYIWLGFIAGLIAIVMVTIMLCCMTSCCSCLKGCCSCGSCCKFDEDDSEPVLKGVKLHYT

Alfa DLQELGKYEQYIKWPWYIWLGFIAGLIAIVMVTIMLCCMTSCCSCLKGCCSCGSCCKFDEDDSEPVLKGVKLHYT

Beta DLQELGKYEQYIKWPWYIWLGFIAGLIAIVMVTIMLCCMTSCCSCLKGCCSCGSCCKFDEDDSEPVLKGVKLHYT

Gamma DLQELGKYEQYIKWPWYIWLGFIAGLIAIVMVTIMLCCMTSCCSCLKGCCSCGSCCKFDEDDSEPVLKGVKLHYT

Delta DLQELGKYEQYIKWPWYIWLGFIAGLIAIVMVTIMLCCMTSCCSCLKGCCSCGSCCKFDEDDSEPVLKGVKLHYT

Omicron DLQELGKYEQYIKWPWYIWLGFIAGLIAIVMVTIMLCCMTSCCSCLKGCCSCGSCCKFDEDDSEPVLKGVKLHYT

***************************************************************************

Suppl. Table 3 **Sequence homology between SARS-CoV-2 VOCs.** The entire proteome of the clinically relevant VOCs of the SARS-CoV-2 have been aligned and the percentage of homology is shown.

|  | Wuhan | Omicron | Delta | Gamma | Alfa | Beta | Average |
| --- | --- | --- | --- | --- | --- | --- | --- |
| Wuhan | 100 | 97,4 | 99,37 | 99,06 | 99,45 | 99,45 | 98,946 |
| Omicron | 97,4 | 100 | 97,31 | 97,08 | 97,32 | 97,39 | 97,3 |
| Delta | 99,37 | 97,31 | 100 | 98,58 | 99,05 | 98,97 | 98,656 |
| Gamma | 99,06 | 97,08 | 98,58 | 100 | 98,82 | 99,06 | 98,52 |
| Alfa | 99,45 | 97,32 | 99,05 | 98,82 | 100 | 99,21 | 98,77 |
| Beta | 99,45 | 97,39 | 98,97 | 99,06 | 99,21 | 100 | 98,816 |

Suppl. Table 4 **Strong binders predicted in the spike proteins from the VOCs.** The SBs predicted in the spike proteins from all the major VOCs are reported. For each of them is indicated the position along the sequence, the VOC and the affinity value (expressed in nanomolarity, nM).

| Pos | MHC | Core | Identity | Aff(nM) | BindLevel |
| --- | --- | --- | --- | --- | --- |
| 107 | HLA-A*02:01 | TLDSKTQSL | Alfa | 175,75 | SB |
| 109 | HLA-A*02:01 | TLDSKTQSL | Beta | 175,75 | SB |
| 109 | HLA-A*02:01 | TLDSKTQSL | Delta | 175,75 | SB |
| 109 | HLA-A*02:01 | TLDSKTQSL | Gamma | 175,75 | SB |
| 107 | HLA-A*02:01 | TLDSKTQSL | Omicron | 175,75 | SB |
| 109 | HLA-A*02:01 | TLDSKTQSL | Wuhan | 175,75 | SB |
|  |  |  |  |  |  |
| 199 | HLA-A*02:01 | KIYSKHTPV | Omicron | 65,37 | SB |
|  |  |  |  |  |  |
| 266 | HLA-A*02:01 | YLQPRTFLL | Alfa | 4,3 | SB |
| 266 | HLA-A*02:01 | YLQPRTFLL | Beta | 4,3 | SB |
| 267 | HLA-A*02:01 | YLQPRTFLL | Delta | 4,3 | SB |
| 269 | HLA-A*02:01 | YLQPRTFLL | Gamma | 4,3 | SB |
| 266 | HLA-A*02:01 | YLQPRTFLL | Omicron | 4,3 | SB |
| 269 | HLA-A*02:01 | YLQPRTFLL | Wuhan | 4,3 | SB |
|  |  |  |  |  |  |
| 414 | HLA-A*02:01 | KIADYNYKL | Alfa | 23,08 | SB |
| 414 | HLA-A*02:01 | NIADYNYKL | Beta | 110,85 | SB |
| 415 | HLA-A*02:01 | KIADYNYKL | Delta | 23,08 | SB |
| 417 | HLA-A*02:01 | TIADYNYKL | Gamma | 69,17 | SB |
| 414 | HLA-A*02:01 | NIADYNYKL | Omicron | 110,85 | SB |
| 417 | HLA-A*02:01 | KIADYNYKL | Wuhan | 23,08 | SB |
|  |  |  |  |  |  |
| 688 | HLA-A*02:01 | SIIAYTMSL | Alfa | 20,36 | SB |
| 688 | HLA-A*02:01 | SIIAYTMSL | Beta | 20,36 | SB |
| 689 | HLA-A*02:01 | SIIAYTMSL | Delta | 20,36 | SB |
| 691 | HLA-A*02:01 | SIIAYTMSL | Gamma | 20,36 | SB |
| 688 | HLA-A*02:01 | SIIAYTMSL | Omicron | 20,36 | SB |
| 691 | HLA-A*02:01 | SIIAYTMSL | Wuhan | 20,36 | SB |
|  |  |  |  |  |  |
| 818 | HLA-A*02:01 | LLFNKVTLA | Alfa | 12,82 | SB |
| 818 | HLA-A*02:01 | LLFNKVTLA | Beta | 12,82 | SB |
| 819 | HLA-A*02:01 | LLFNKVTLA | Delta | 12,82 | SB |
| 821 | HLA-A*02:01 | LLFNKVTLA | Gamma | 12,82 | SB |
| 818 | HLA-A*02:01 | LLFNKVTLA | Omicron | 12,82 | SB |
| 821 | HLA-A*02:01 | LLFNKVTLA | Wuhan | 12,82 | SB |
|  |  |  |  |  |  |
| 854 | HLA-A*02:01 | GLTVLPPLL | Alfa | 100,5 | SB |
| 854 | HLA-A*02:01 | GLTVLPPLL | Beta | 100,5 | SB |
| 855 | HLA-A*02:01 | GLTVLPPLL | Delta | 100,5 | SB |
| 857 | HLA-A*02:01 | GLTVLPPLL | Gamma | 100,5 | SB |
| 854 | HLA-A*02:01 | GLTVLPPLL | Omicron | 100,5 | SB |
| 857 | HLA-A*02:01 | GLTVLPPLL | Wuhan | 100,5 | SB |
|  |  |  |  |  |  |
| 912 | HLA-A*02:01 | VLYENQKLI | Alfa | 359,26 | SB |
| 912 | HLA-A*02:01 | VLYENQKLI | Beta | 359,26 | SB |
| 913 | HLA-A*02:01 | VLYENQKLI | Delta | 359,26 | SB |
| 915 | HLA-A*02:01 | VLYENQKLI | Gamma | 359,26 | SB |
| 912 | HLA-A*02:01 | VLYENQKLI | Omicron | 359,26 | SB |
| 915 | HLA-A*02:01 | VLYENQKLI | Wuhan | 359,26 | SB |
|  |  |  |  |  |  |
| 941 | HLA-A*02:01 | SALGKLQNV | Delta | 642,72 | SB |
|  |  |  |  |  |  |
| 955 | HLA-A*02:01 | ALNTLVKQL | Alfa | 563,85 | SB |
| 955 | HLA-A*02:01 | ALNTLVKQL | Beta | 563,85 | SB |
| 956 | HLA-A*02:01 | ALNTLVKQL | Delta | 563,85 | SB |
| 958 | HLA-A*02:01 | ALNTLVKQL | Gamma | 563,85 | SB |
| 955 | HLA-A*02:01 | ALNTLVKQL | Omicron | 563,85 | SB |
| 958 | HLA-A*02:01 | ALNTLVKQL | Wuhan | 563,85 | SB |
|  |  |  |  |  |  |
| 973 | HLA-A*02:01 | VLNDILARL | Alfa | 12,95 | SB |
| 973 | HLA-A*02:01 | VLNDILSRL | Beta | 22,77 | SB |
| 974 | HLA-A*02:01 | VLNDILSRL | Delta | 22,77 | SB |
| 976 | HLA-A*02:01 | VLNDILSRL | Gamma | 22,77 | SB |
| 973 | HLA-A*02:01 | VLNDIFSRL | Omicron | 24,47 | SB |
| 976 | HLA-A*02:01 | VLNDILSRL | Wuhan | 22,77 | SB |
|  |  |  |  |  |  |
| 980 | HLA-A*02:01 | RLDKVEAEV | Alfa | 46,73 | SB |
| 980 | HLA-A*02:01 | RLDKVEAEV | Beta | 46,73 | SB |
| 981 | HLA-A*02:01 | RLDKVEAEV | Delta | 46,73 | SB |
| 983 | HLA-A*02:01 | RLDKVEAEV | Gamma | 46,73 | SB |
| 980 | HLA-A*02:01 | RLDKVEAEV | Omicron | 46,73 | SB |
| 983 | HLA-A*02:01 | RLDKVEAEV | Wuhan | 46,73 | SB |
|  |  |  |  |  |  |
| 997 | HLA-A*02:01 | RLQSLQTYV | Alfa | 11,92 | SB |
| 997 | HLA-A*02:01 | RLQSLQTYV | Beta | 11,92 | SB |
| 998 | HLA-A*02:01 | RLQSLQTYV | Delta | 11,92 | SB |
| 1000 | HLA-A*02:01 | RLQSLQTYV | Gamma | 11,92 | SB |
| 997 | HLA-A*02:01 | RLQSLQTYV | Omicron | 11,92 | SB |
| 1000 | HLA-A*02:01 | RLQSLQTYV | Wuhan | 11,92 | SB |
|  |  |  |  |  |  |
| 1045 | HLA-A*02:01 | HLMSFPQSA | Alfa | 41,79 | SB |
| 1045 | HLA-A*02:01 | HLMSFPQSA | Beta | 41,79 | SB |
| 1046 | HLA-A*02:01 | HLMSFPQSA | Delta | 41,79 | SB |
| 1048 | HLA-A*02:01 | HLMSFPQSA | Gamma | 41,79 | SB |
| 1045 | HLA-A*02:01 | HLMSFPQSA | Omicron | 41,79 | SB |
| 1048 | HLA-A*02:01 | HLMSFPQSA | Wuhan | 41,79 | SB |
|  |  |  |  |  |  |
| 1057 | HLA-A*02:01 | VVFLHVTYV | Alfa | 17,07 | SB |
| 1057 | HLA-A*02:01 | VVFLHVTYV | Beta | 17,07 | SB |
| 1058 | HLA-A*02:01 | VVFLHVTYV | Delta | 17,07 | SB |
| 1060 | HLA-A*02:01 | VVFLHVTYV | Gamma | 17,07 | SB |
| 1057 | HLA-A*02:01 | VVFLHVTYV | Omicron | 17,07 | SB |
| 1060 | HLA-A*02:01 | VVFLHVTYV | Wuhan | 17,07 | SB |
|  |  |  |  |  |  |
| 1182 | HLA-A*02:01 | RLNEVAKNL | Alfa | 246,13 | SB |
| 1182 | HLA-A*02:01 | RLNEVAKNL | Beta | 246,13 | SB |
| 1183 | HLA-A*02:01 | RLNEVAKNL | Delta | 246,13 | SB |
| 1185 | HLA-A*02:01 | RLNEVAKNL | Gamma | 246,13 | SB |
| 1182 | HLA-A*02:01 | RLNEVAKNL | Omicron | 246,13 | SB |
| 1185 | HLA-A*02:01 | RLNEVAKNL | Wuhan | 246,13 | SB |
|  |  |  |  |  |  |
| 1189 | HLA-A*02:01 | NLNESLIDL | Alfa | 89,53 | SB |
| 1189 | HLA-A*02:01 | NLNESLIDL | Beta | 89,53 | SB |
| 1190 | HLA-A*02:01 | NLNESLIDL | Delta | 89,53 | SB |
| 1192 | HLA-A*02:01 | NLNESLIDL | Gamma | 89,53 | SB |
| 1189 | HLA-A*02:01 | NLNESLIDL | Omicron | 89,53 | SB |
| 1192 | HLA-A*02:01 | NLNESLIDL | Wuhan | 89,53 | SB |
|  |  |  |  |  |  |
| 1217 | HLA-A*02:01 | FIAGLIAIV | Alfa | 6,61 | SB |
| 1217 | HLA-A*02:01 | FIAGLIAIV | Beta | 6,61 | SB |
| 1218 | HLA-A*02:01 | FIAGLIAIV | Delta | 6,61 | SB |
| 1220 | HLA-A*02:01 | FIAGLIAIV | Gamma | 6,61 | SB |
| 1217 | HLA-A*02:01 | FIAGLIAIV | Omicron | 6,61 | SB |
| 1220 | HLA-A*02:01 | FIAGLIAIV | Wuhan | 6,61 | SB |

Suppl. Table 5 **Weak binders predicted in the spike proteins from the VOCs.** The WBs predicted in the spike proteins from all the major VOCs are reported. For each of them is indicated the position along the sequence, the VOC and the affinity value (expressed in nanomolarity, nM).

| Pos | MHC | Core | Identity | Aff(nM) | BindLevel |
| --- | --- | --- | --- | --- | --- |
| 28 | HLA-A*02:01 | YTNSFTRGV | Alfa | 328 | WB |
| 28 | HLA-A*02:01 | YTNSFTRGV | Beta | 328 | WB |
| 28 | HLA-A*02:01 | YTNSFTRGV | Delta | 328 | WB |
| 28 | HLA-A*02:01 | YTNSFTRGV | Gamma | 328 | WB |
| 28 | HLA-A*02:01 | YTNSFTRGV | Omicron | 328 | WB |
| 28 | HLA-A*02:01 | YTNSFTRGV | Wuhan | 328 | WB |
|  |  |  |  |  |  |
| 62 | HLA-A*02:01 | VTWFHAIHV | Beta | 134,02 | WB |
| 62 | HLA-A*02:01 | VTWFHAIHV | Delta | 134,02 | WB |
| 62 | HLA-A*02:01 | VTWFHAIHV | Gamma | 134,02 | WB |
| 59 | HLA-A*02:01 | VTWFHAIHV | Omicron | 134,02 | WB |
| 62 | HLA-A*02:01 | VTWFHAIHV | Wuhan | 134,02 | WB |
|  |  |  |  |  |  |
| 131 | HLA-A*02:01 | FQFCNDPFL | Alfa | 10,54 | WB |
| 133 | HLA-A*02:01 | FQFCNDPFL | Beta | 10,54 | WB |
| 133 | HLA-A*02:01 | FQFCNDPFL | Delta | 10,54 | WB |
| 133 | HLA-A*02:01 | FQFCNYPFL | Gamma | 6,76 | WB |
| 131 | HLA-A*02:01 | FQFCNDPFL | Omicron | 10,54 | WB |
| 133 | HLA-A*02:01 | FQFCNDPFL | Wuhan | 10,54 | WB |
|  |  |  |  |  |  |
| 133 | HLA-A*02:01 | FCNDPFLGV | Alfa | 555,89 | WB |
| 135 | HLA-A*02:01 | FCNDPFLGV | Beta | 555,89 | WB |
| 135 | HLA-A*02:01 | FCNDPFLGV | Delta | 555,89 | WB |
| 135 | HLA-A*02:01 | FCNDPFLGV | Wuhan | 555,89 | WB |
|  |  |  |  |  |  |
| 199 | HLA-A*02:01 | KIYSKHTPI | Alfa | 463,64 | WB |
| 202 | HLA-A*02:01 | KIYSKHTPI | Beta | 463,64 | WB |
| 200 | HLA-A*02:01 | KIYSKHTPI | Delta | 463,64 | WB |
| 202 | HLA-A*02:01 | KIYSKHTPI | Gamma | 463,64 | WB |
| 197 | HLA-A*02:01 | KIYSKHTPI | Omicron | 463,64 | WB |
| 202 | HLA-A*02:01 | KIYSKHTPI | Wuhan | 463,64 | WB |
|  |  |  |  |  |  |
| 215 | HLA-A*02:01 | GLPQGFSAL | Beta | 307,63 | WB |
|  |  |  |  |  |  |
| 222 | HLA-A*02:01 | PLVDLPIGI | Alfa | 1130,35 | WB |
| 225 | HLA-A*02:01 | PLVDLPIGI | Beta | 1130,35 | WB |
| 223 | HLA-A*02:01 | PLVDLPIGI | Delta | 1130,35 | WB |
| 225 | HLA-A*02:01 | PLVDLPIGI | Gamma | 1130,35 | WB |
| 222 | HLA-A*02:01 | PLVDLPIGI | Omicron | 1130,35 | WB |
| 225 | HLA-A*02:01 | PLVDLPIGI | Wuhan | 1130,35 | WB |
|  |  |  |  |  |  |
| 238 | HLA-A*02:01 | LLALHRSYL | Alfa | 353,28 | WB |
| 239 | HLA-A*02:01 | LLALHRSYL | Delta | 353,28 | WB |
| 241 | HLA-A*02:01 | LLALHRSYL | Gamma | 353,28 | WB |
| 238 | HLA-A*02:01 | LLALHRSYL | Omicron | 353,28 | WB |
| 241 | HLA-A*02:01 | LLALHRSYL | Wuhan | 353,28 | WB |
|  |  |  |  |  |  |
| 364 | HLA-A*02:01 | VLYNLAPFF | Omicron | 614,19 | WB |
|  |  |  |  |  |  |
| 421 | HLA-A*02:01 | KLPDDFTGC | Alfa | 450,41 | WB |
| 421 | HLA-A*02:01 | KLPDDFTGC | Beta | 450,41 | WB |
| 422 | HLA-A*02:01 | KLPDDFTGC | Delta | 450,41 | WB |
| 424 | HLA-A*02:01 | KLPDDFTGC | Gamma | 450,41 | WB |
| 421 | HLA-A*02:01 | KLPDDFTGC | Omicron | 450,41 | WB |
| 424 | HLA-A*02:01 | KLPDDFTGC | Wuhan | 450,41 | WB |
|  |  |  |  |  |  |
| 441 | HLA-A*02:01 | KVSGNYNYL | Omicron | 990,33 | WB |
|  |  |  |  |  |  |
| 492 | HLA-A*02:01 | YGFQPTYGV | Alfa | 459,54 | WB |
| 492 | HLA-A*02:01 | YGFQPTYGV | Beta | 459,54 | WB |
| 495 | HLA-A*02:01 | YGFQPTYGV | Gamma | 459,54 | WB |
| 492 | HLA-A*02:01 | YSFRPTYGV | Omicron | 87,67 | WB |
|  |  |  |  |  |  |
| 498 | HLA-A*02:01 | GVGHQPYRV | Omicron | 3189,76 | WB |
|  |  |  |  |  |  |
| 502 | HLA-A*02:01 | YQPYRVVVL | Alfa | 1377,75 | WB |
| 502 | HLA-A*02:01 | YQPYRVVVL | Beta | 1377,75 | WB |
| 503 | HLA-A*02:01 | YQPYRVVVL | Delta | 1377,75 | WB |
| 505 | HLA-A*02:01 | YQPYRVVVL | Gamma | 1377,75 | WB |
| 505 | HLA-A*02:01 | YQPYRVVVL | Wuhan | 1377,75 | WB |
|  |  |  |  |  |  |
| 509 | HLA-A*02:01 | VLSFELLHA | Alfa | 118,62 | WB |
| 509 | HLA-A*02:01 | VLSFELLHA | Beta | 118,62 | WB |
| 510 | HLA-A*02:01 | VLSFELLHA | Delta | 118,62 | WB |
| 512 | HLA-A*02:01 | VLSFELLHA | Gamma | 118,62 | WB |
| 509 | HLA-A*02:01 | VLSFELLHA | Omicron | 118,62 | WB |
| 512 | HLA-A*02:01 | VLSFELLHA | Wuhan | 118,62 | WB |
|  |  |  |  |  |  |
| 513 | HLA-A*02:01 | ELLHAPATV | Alfa | 386,28 | WB |
| 513 | HLA-A*02:01 | ELLHAPATV | Beta | 386,28 | WB |
| 514 | HLA-A*02:01 | ELLHAPATV | Delta | 386,28 | WB |
| 516 | HLA-A*02:01 | ELLHAPATV | Gamma | 386,28 | WB |
| 513 | HLA-A*02:01 | ELLHAPATV | Omicron | 386,28 | WB |
| 516 | HLA-A*02:01 | ELLHAPATV | Wuhan | 386,28 | WB |
|  |  |  |  |  |  |
| 581 | HLA-A*02:01 | ILDITPCSF | Alfa | 2524,42 | WB |
| 581 | HLA-A*02:01 | ILDITPCSF | Beta | 2524,42 | WB |
| 582 | HLA-A*02:01 | ILDITPCSF | Delta | 2524,42 | WB |
| 584 | HLA-A*02:01 | ILDITPCSF | Gamma | 2524,42 | WB |
| 581 | HLA-A*02:01 | ILDITPCSF | Omicron | 2524,42 | WB |
| 584 | HLA-A*02:01 | ILDITPCSF | Wuhan | 2524,42 | WB |
|  |  |  |  |  |  |
| 607 | HLA-A*02:01 | VLYQGVNCT | Alfa | 733,09 | WB |
| 607 | HLA-A*02:01 | VLYQGVNCT | Beta | 733,09 | WB |
| 608 | HLA-A*02:01 | VLYQGVNCT | Delta | 733,09 | WB |
| 610 | HLA-A*02:01 | VLYQGVNCT | Gamma | 733,09 | WB |
| 607 | HLA-A*02:01 | VLYQGVNCT | Omicron | 733,09 | WB |
| 610 | HLA-A*02:01 | VLYQDVNCT | Wuhan | 1342,97 | WB |
|  |  |  |  |  |  |
| 612 | HLA-A*02:01 | YQDVNCTEV | Wuhan | 75,67 | WB |
|  |  |  |  |  |  |
| 631 | HLA-A*02:01 | RVYSTGSNV | Alfa | 1163,21 | WB |
| 631 | HLA-A*02:01 | RVYSTGSNV | Beta | 1163,21 | WB |
| 632 | HLA-A*02:01 | RVYSTGSNV | Delta | 1163,21 | WB |
| 634 | HLA-A*02:01 | RVYSTGSNV | Gamma | 1163,21 | WB |
| 631 | HLA-A*02:01 | RVYSTGSNV | Omicron | 1163,21 | WB |
| 634 | HLA-A*02:01 | RVYSTGSNV | Wuhan | 1163,21 | WB |
|  |  |  |  |  |  |
| 709 | HLA-A*02:01 | IAIPINFTI | Alfa | 325,6 | WB |
|  |  |  |  |  |  |
| 715 | HLA-A*02:01 | FTISVTTEI | Alfa | 58,89 | WB |
| 715 | HLA-A*02:01 | FTISVTTEI | Beta | 58,89 | WB |
| 716 | HLA-A*02:01 | FTISVTTEI | Delta | 58,89 | WB |
| 718 | HLA-A*02:01 | FTISVTTEI | Gamma | 58,89 | WB |
| 715 | HLA-A*02:01 | FTISVTTEI | Omicron | 58,89 | WB |
| 718 | HLA-A*02:01 | FTISVTTEI | Wuhan | 58,89 | WB |
|  |  |  |  |  |  |
| 718 | HLA-A*02:01 | SVTTEILPV | Alfa | 147,18 | WB |
| 718 | HLA-A*02:01 | SVTTEILPV | Beta | 147,18 | WB |
| 719 | HLA-A*02:01 | SVTTEILPV | Delta | 147,18 | WB |
| 721 | HLA-A*02:01 | SVTTEILPV | Gamma | 147,18 | WB |
| 718 | HLA-A*02:01 | SVTTEILPV | Omicron | 147,18 | WB |
| 721 | HLA-A*02:01 | SVTTEILPV | Wuhan | 147,18 | WB |
|  |  |  |  |  |  |
| 759 | HLA-A*02:01 | QLNRALTGI | Alfa | 513,78 | WB |
| 759 | HLA-A*02:01 | QLNRALTGI | Beta | 513,78 | WB |
| 760 | HLA-A*02:01 | QLNRALTGI | Delta | 513,78 | WB |
| 762 | HLA-A*02:01 | QLNRALTGI | Gamma | 513,78 | WB |
| 762 | HLA-A*02:01 | QLNRALTGI | Wuhan | 513,78 | WB |
|  |  |  |  |  |  |
| 770 | HLA-A*02:01 | EQDKNTQEV | Alfa | 8085,04 | WB |
| 770 | HLA-A*02:01 | EQDKNTQEV | Beta | 8085,04 | WB |
| 771 | HLA-A*02:01 | EQDKNTQEV | Delta | 8085,04 | WB |
| 773 | HLA-A*02:01 | EQDKNTQEV | Gamma | 8085,04 | WB |
| 770 | HLA-A*02:01 | EQDKNTQEV | Omicron | 8085,04 | WB |
| 773 | HLA-A*02:01 | EQDKNTQEV | Wuhan | 8085,04 | WB |
|  |  |  |  |  |  |
| 774 | HLA-A*02:01 | NTQEVFAQV | Alfa | 497,1 | WB |
| 774 | HLA-A*02:01 | NTQEVFAQV | Beta | 497,1 | WB |
| 775 | HLA-A*02:01 | NTQEVFAQV | Delta | 497,1 | WB |
| 777 | HLA-A*02:01 | NTQEVFAQV | Gamma | 497,1 | WB |
| 774 | HLA-A*02:01 | NTQEVFAQV | Omicron | 497,1 | WB |
| 777 | HLA-A*02:01 | NTQEVFAQV | Wuhan | 497,1 | WB |
|  |  |  |  |  |  |
| 783 | HLA-A*02:01 | KQIYKTPPI | Alfa | 118,46 | WB |
| 783 | HLA-A*02:01 | KQIYKTPPI | Beta | 118,46 | WB |
| 784 | HLA-A*02:01 | KQIYKTPPI | Delta | 118,46 | WB |
| 786 | HLA-A*02:01 | KQIYKTPPI | Gamma | 118,46 | WB |
| 783 | HLA-A*02:01 | KQIYKTPPI | Omicron | 118,46 | WB |
| 786 | HLA-A*02:01 | KQIYKTPPI | Wuhan | 118,46 | WB |
|  |  |  |  |  |  |
| 866 | HLA-A*02:01 | MIAQYTSAL | Alfa | 105,71 | WB |
| 866 | HLA-A*02:01 | MIAQYTSAL | Beta | 105,71 | WB |
| 867 | HLA-A*02:01 | MIAQYTSAL | Delta | 105,71 | WB |
| 869 | HLA-A*02:01 | MIAQYTSAL | Gamma | 105,71 | WB |
| 866 | HLA-A*02:01 | MIAQYTSAL | Omicron | 105,71 | WB |
| 869 | HLA-A*02:01 | MIAQYTSAL | Wuhan | 105,71 | WB |
|  |  |  |  |  |  |
| 873 | HLA-A*02:01 | ALLAGTITS | Alfa | 729,27 | WB |
| 873 | HLA-A*02:01 | ALLAGTITS | Beta | 729,27 | WB |
| 874 | HLA-A*02:01 | ALLAGTITS | Delta | 729,27 | WB |
| 876 | HLA-A*02:01 | ALLAGTITS | Gamma | 729,27 | WB |
| 873 | HLA-A*02:01 | ALLAGTITS | Omicron | 729,27 | WB |
| 876 | HLA-A*02:01 | ALLAGTITS | Wuhan | 729,27 | WB |
|  |  |  |  |  |  |
| 891 | HLA-A*02:01 | LQIPFAMQM | Alfa | 863,81 | WB |
| 891 | HLA-A*02:01 | LQIPFAMQM | Beta | 863,81 | WB |
| 892 | HLA-A*02:01 | LQIPFAMQM | Delta | 863,81 | WB |
| 894 | HLA-A*02:01 | LQIPFAMQM | Gamma | 863,81 | WB |
| 891 | HLA-A*02:01 | LQIPFAMQM | Omicron | 863,81 | WB |
| 894 | HLA-A*02:01 | LQIPFAMQM | Wuhan | 863,81 | WB |
|  |  |  |  |  |  |
| 934 | HLA-A*02:01 | SLSSTASAL | Alfa | 435,97 | WB |
| 934 | HLA-A*02:01 | SLSSTASAL | Beta | 435,97 | WB |
| 935 | HLA-A*02:01 | SLSSTASAL | Delta | 435,97 | WB |
| 937 | HLA-A*02:01 | SLSSTASAL | Gamma | 435,97 | WB |
| 934 | HLA-A*02:01 | SLSSTASAL | Omicron | 435,97 | WB |
| 937 | HLA-A*02:01 | SLSSTASAL | Wuhan | 435,97 | WB |
|  |  |  |  |  |  |
| 941 | HLA-A*02:01 | ALGKLQDVV | Alfa | 852,18 | WB |
| 941 | HLA-A*02:01 | ALGKLQDVV | Beta | 852,18 | WB |
| 942 | HLA-A*02:01 | ALGKLQNVV | Delta | 604,78 | WB |
| 944 | HLA-A*02:01 | ALGKLQDVV | Gamma | 852,18 | WB |
| 941 | HLA-A*02:01 | ALGKLQDVV | Omicron | 852,18 | WB |
| 944 | HLA-A*02:01 | ALGKLQDVV | Wuhan | 852,18 | WB |
|  |  |  |  |  |  |
| 993 | HLA-A*02:01 | LITGRLQSL | Alfa | 3501,5 | WB |
| 993 | HLA-A*02:01 | LITGRLQSL | Beta | 3501,5 | WB |
| 994 | HLA-A*02:01 | LITGRLQSL | Delta | 3501,5 | WB |
| 996 | HLA-A*02:01 | LITGRLQSL | Gamma | 3501,5 | WB |
| 993 | HLA-A*02:01 | LITGRLQSL | Omicron | 3501,5 | WB |
| 996 | HLA-A*02:01 | LITGRLQSL | Wuhan | 3501,5 | WB |
|  |  |  |  |  |  |
| 1004 | HLA-A*02:01 | YVTQQLIRA | Alfa | 3246,41 | WB |
| 1004 | HLA-A*02:01 | YVTQQLIRA | Beta | 3246,41 | WB |
| 1005 | HLA-A*02:01 | YVTQQLIRA | Delta | 3246,41 | WB |
| 1007 | HLA-A*02:01 | YVTQQLIRA | Gamma | 3246,41 | WB |
| 1004 | HLA-A*02:01 | YVTQQLIRA | Omicron | 3246,41 | WB |
| 1007 | HLA-A*02:01 | YVTQQLIRA | Wuhan | 3246,41 | WB |
|  |  |  |  |  |  |
| 1059 | HLA-A*02:01 | FLHVTYVPA | Alfa | 34,61 | WB |
| 1059 | HLA-A*02:01 | FLHVTYVPA | Beta | 34,61 | WB |
| 1060 | HLA-A*02:01 | FLHVTYVPA | Delta | 34,61 | WB |
| 1062 | HLA-A*02:01 | FLHVTYVPA | Gamma | 34,61 | WB |
| 1059 | HLA-A*02:01 | FLHVTYVPA | Omicron | 34,61 | WB |
| 1062 | HLA-A*02:01 | FLHVTYVPA | Wuhan | 34,61 | WB |
|  |  |  |  |  |  |
| 1111 | HLA-A*02:01 | IITTHNTFV | Alfa | 391,41 | WB |
|  |  |  |  |  |  |
| 1134 | HLA-A*02:01 | VYDPLQPEL | Alfa | 8641,52 | WB |
| 1134 | HLA-A*02:01 | VYDPLQPEL | Beta | 8641,52 | WB |
| 1135 | HLA-A*02:01 | VYDPLQPEL | Delta | 8641,52 | WB |
| 1137 | HLA-A*02:01 | VYDPLQPEL | Gamma | 8641,52 | WB |
| 1134 | HLA-A*02:01 | VYDPLQPEL | Omicron | 8641,52 | WB |
| 1137 | HLA-A*02:01 | VYDPLQPEL | Wuhan | 8641,52 | WB |
|  |  |  |  |  |  |
| 1141 | HLA-A*02:01 | ELDSFKEEL | Alfa | 4841,91 | WB |
| 1141 | HLA-A*02:01 | ELDSFKEEL | Beta | 4841,91 | WB |
| 1142 | HLA-A*02:01 | ELDSFKEEL | Delta | 4841,91 | WB |
| 1144 | HLA-A*02:01 | ELDSFKEEL | Gamma | 4841,91 | WB |
| 1141 | HLA-A*02:01 | ELDSFKEEL | Omicron | 4841,91 | WB |
| 1144 | HLA-A*02:01 | ELDSFKEEL | Wuhan | 4841,91 | WB |
|  |  |  |  |  |  |
| 1168 | HLA-A*02:01 | GINASVVNI | Alfa | 1383,16 | WB |
| 1168 | HLA-A*02:01 | GINASVVNI | Beta | 1383,16 | WB |
| 1169 | HLA-A*02:01 | GINASVVNI | Delta | 1383,16 | WB |
| 1168 | HLA-A*02:01 | GINASVVNI | Omicron | 1383,16 | WB |
| 1171 | HLA-A*02:01 | GINASVVNI | Wuhan | 1383,16 | WB |

Suppl. Table 6 **New predicted weak binders (WB) in VOCs other than Wuhan.** Green background indicates unique predicted WBs; blue background indicates shared WBs. Values in the boxes indicate affinity to HLA-A*02:01 allele.

| **Position** | **Beta** | **Omicron** | **Alfa** | **Gamma** |
| --- | --- | --- | --- | --- |
| 215 | **307.63** |  |  |  |
| 364 |  | **614.19** |  |  |
| 441 |  | **990.33** |  |  |
| 492 | **459.54** | **87.67** | **459.54** | **459.54** |
| 498 |  | **3189.76** |  |  |
| 709 |  |  | **325.60** |  |
| 1111 |  |  | **391.41** |  |

Suppl. Table 7 **Compared prediction of spike SB+WB by all algorithms.** The prediction of SB and WB spike epitopes predicted by NetMHC – Pan4.1 (<100nM) was assessed also with the indicated algorithms. Ranking in the first 16 positions in all methods is indicated. 12 epitopes predicted by NetMHC – Pan4.1 are found to be best ranking also in the other methods

| **Peptide** | **NetMHC 4.1** | **ANN 4.0** | **SMM** | **MHC Flurry 2.0** |
| --- | --- | --- | --- | --- |
| **YLQPRTFLL** | 1 | 1 | 1 | 1 |
| **FIAGLIAIV** | 2 | 3 | 2 | 2 |
| **FQFCNDPFL** | 3 | 2 | 7 | 7 |
| **LLFNKVTLA** | 5 | 7 | 9 | 9 |
| **VVFLHVTYV** | 6 | 10 | 8 | 6 |
| **SIIAYTMSL** | 7 | 4 | 11 | 10 |
| **KIADYNYKL** | 8 | 9 | 4 | 3 |
| **VLNDIFSRL** | 9 | 13 | 10 | 4 |
| **FLHVTYVPA** | 10 | 14 | 13 | 14 |
| **RLDKVEAEV** | 12 | 11 | 6 | 17 |
| **FTISVTTEI** | 13 | 6 | 15 | 8 |
| **GLTVLPPLL** | 16 | 19 | 20 | 19 |

.

Suppl. Table 8 **Strong binders predicted in the SARS-CoV-2 (no spike) proteins from the VOCs.** The SBs predicted in the proteins (no spike) from all the major VOCs are reported. For each protein are indicated the epitope sequences, and the affinity value (expressed in nanomolarity, nM). Green color indicates the omicron variant; Red color the alpha variant.

| **PROTEIN** | **EPITOPES** | **AFF.nM** | **BINDING LEVEL** |
| --- | --- | --- | --- |
| **ORF1ab** | FLAHIQWMV | 2,01 | SB |
|  | ILFTRFFYV | 2,32 | SB |
|  | FLLPSLATV | 2,41 | SB |
|  | YLDAYNMMI | 2,63 | SB |
|  | FLLNKEMYL | 2,67 | SB |
|  | TLMNVLTLV | 3,31 | SB |
|  | YLNTLTLAV | 3,42 | SB |
|  | SMWALIISV | 3,8 | SB |
|  | TMADLVYAL | 4,08 | SB |
|  | VLFSTVFPL | 4,23 | SB |
|  | LLLDDFVEI | 4,5 | SB |
|  | VLWAHGFEL | 4,54 | SB |
|  | ALLADKFPV | 4,7 | SB |
|  | NLIDSYFVV | 4,85 | SB |
|  | FVNEFYAYL | 4,97 | SB |
|  | FLPRVFSAV | 5,52 | SB |
|  | ALWEIQQVV | 5,73 | SB |
|  | RIMTWLDMV | 6,02 | SB |
|  | FLARGIVFM | 6,13 | SB |
|  | YLATALLTL | 6,4 | SB |
|  | YTMADLVYA | 6,42 | SB |
|  | KLIEYTDFA | 6,45 | SB |
|  | LMIERFVSL | 6,57 | SB |
|  | FLNRFTTTL | 6,61 | SB |
|  | FLARGVVFM | 6,68 | SB |
|  | ILTSLLVLV | 6,75 | SB |
|  | MMISAGFSL | 6,83 | SB |
|  | MLDMYSVML | 6,9 | SB |
|  | KLNVGDYFV | 7,35 | SB |
|  | YLTNDVSFL | 7,38 | SB |
|  | SLPGVFCGV | 7,48 | SB |
|  | FVDGVPFVV | 7,54 | SB |
|  | KLSYGIATV | 8,63 | SB |
|  | KLWAQCVQL | 8,97 | SB |
|  | VMVELVAEL | 10,56 | SB |
|  | NLSDRVVFV | 10,8 | SB |
|  | VMCGGSLYV | 11,11 | SB |
|  | TLIGDCATV | 11,38 | SB |
|  | LLLTILTSL | 11,67 | SB |
|  | YLNSTNVTI | 12,5 | SB |
|  | LVLSVNPYV | 12,85 | SB |
|  | ILSPLYAFA | 13,11 | SB |
|  | QLMCQPILL | 13,21 | SB |
|  | LLADKFPVL | 13,31 | SB |
|  | YVWKSYVHV | 14,63 | SB |
|  | GLNDNLLEI | 15,5 | SB |
|  | LQLGFSTGV | 15,73 | SB |
|  | ILGTVSWNL | 16,02 | SB |
|  | QMAPISAMV | 16,04 | SB |
|  | LLMPILTLT | 16,52 | SB |
|  | RLIDAMMFT | 16,55 | SB |
|  | RQLLFVVEV | 17,02 | SB |
|  | SLLSVLLSM | 17,23 | SB |
|  | YLASGGQPI | 17,3 | SB |
|  | ILLLDQALV | 17,57 | SB |
|  | VLLSVLQQL | 17,88 | SB |
|  | KLKDCVMYA | 18,2 | SB |
|  | TLVPQEHYV | 19,5 | SB |
|  | RLANECAQV | 19,62 | SB |
|  | AVIKTLQPV | 20,84 | SB |
|  | FLKKDAPYI | 21,76 | SB |
|  | WLDMVDTSL | 23,07 | SB |
|  | NLLKDCPAV | 23,28 | SB |
|  | KLVNKFLAL | 25,98 | SB |
|  | SLENVAFNV | 26,04 | SB |
|  | SQLGGLHLL | 27,34 | SB |
|  | KLMPVCVET | 27,59 | SB |
|  | NLWNTFTRL | 31,07 | SB |
|  | YLITPVHVM | 31,75 | SB |
|  | TLGVLVPHV | 31,99 | SB |
|  | MLAKALRKV | 32,09 | SB |
|  | TLSEQLDFI | 34,09 | SB |
|  | TLNDLNETL | 34,61 | SB |
|  | KLNEEIAII | 34,66 | SB |
|  | KMFDAYVNT | 34,86 | SB |
|  | KLNIKLLGV | 35,39 | SB |
|  | FLRDGWEIV | 36,71 | SB |
|  | LLDDFVEII | 38,08 | SB |
|  | VLGSLAATV | 43,61 | SB |
|  | VLLAPLLSA | 46,66 | SB |
|  | SLLMPILTL | 47,58 | SB |
|  | ALLSDLQDL | 48,1 | SB |
|  | SMQNCVLKL | 50,58 | SB |
|  | YITGGVVQL | 51,78 | SB |
|  | MLSDTLKNL | 52,66 | SB |
|  | TIIQTIVEV | 53,12 | SB |
|  | SLINTLNDL | 54,87 | SB |
|  | KLVSSFLEM | 58,34 | SB |
|  | ALNLGETFV | 58,55 | SB |
|  | NLAKHCLHV | 61,81 | SB |
|  | YLAVFDKNL | 61,9 | SB |
|  | KLKPVLDWL | 63,18 | SB |
|  | GLFKDCSKV | 65,7 | SB |
|  | TVYEKLKPV | 66,87 | SB |
|  | FITESKPSV | 71,77 | SB |
|  | NVLTLVYKV | 73,8 | SB |
|  | GVYDYLVST | 75,07 | SB |
|  | ALQDAYYRA | 80,3 | SB |
|  | FLNKVVSTT | 83,03 | SB |
|  | NLYDKLVSS | 92,69 | SB |
|  | VLSDRELHL | 93,65 | SB |
| ENVELOPE | FLAFVVFLL | 5,97 | SB |
|  | SLVKPSFYV | 8,97 | SB |
| ORF3A | LLYDANYFL | 2,39 | SB |
|  | YLYALVYFL | 2,72 | SB |
|  | ALSKGVHFV | 8,9 | SB |
|  | TVYSHLLLV | 15,05 | SB |
|  | ALVYFLQSI | 20,25 | SB |
|  | ALLAVFHSA | 21,8 | SB |
|  | ALLAVFQSA | 42,35 | SB |
| MEMBRANE | FVLAAVYRI | 9,17 | SB |
|  | GLMWLSYFI | 3,98 | SB |
|  | KLLEEWNLV | 6,49 | SB |
|  | KLLEQWNLV | 7,24 | SB |
|  | SMWSFNPET | 29,89 | SB |
| NUCLEOCAPSIDE | GMSRIGMEV | 46,4 | SB |
|  | LLLDRLNQL | 10,55 | SB |
| ORF6 | HLVDFQVTI | 18,68 | SB |
| ORF7a | ELYSPIFLI | 99,51 | SB |
|  | ILFLALITL | 66,03 | SB |
|  | KLFIRQEEV | 24,25 | SB |
| ORF8 | FLEYHDVRV | 22,17 | SB |
|  | YIDIGNYTV | 8,07 | SB |
| ORF10 | NVFAFPFTI | 11 | SB |

Suppl. Table 9 **Weak binders predicted in the SARS-CoV-2 (no spike) proteins from the VOCs.** The SBs predicted in the proteins (no spike) from all the major VOCs are reported. For each protein are indicated the epitope sequences, and the affinity value (expressed in nanomolarity, nM). Red color indicates the alpha variant.

| **PROTEIN** | **EPITOPES** | **AFF.nM** | **BINDING LEVEL** |
| --- | --- | --- | --- |
| **ORF1ab** | QLFFSYFAV | 7,15 | WB |
|  | ILHCANFNV | 7,37 | WB |
|  | VLSFCAFAV | 7,66 | WB |
|  | VLAWLYAAV | 9,1 | WB |
|  | WLMWLIINL | 9,18 | WB |
|  | FVAAIFYLI | 9,83 | WB |
|  | LLFLMSFTV | 9,96 | WB |
|  | WMVMFTPLV | 10,24 | WB |
|  | YMPYFFTLL | 10,78 | WB |
|  | AIFYLITPV | 12,49 | WB |
|  | FLGRYMSAL | 12,85 | WB |
|  | LLSAGIFGA | 15,71 | WB |
|  | FLALCADSI | 16,26 | WB |
|  | LLTNMFTPL | 16,66 | WB |
|  | TQWSLFFFL | 17,16 | WB |
|  | FLNGSCGSV | 19,55 | WB |
|  | TLGVYDYLV | 20,8 | WB |
|  | NVLAWLYAA | 24,16 | WB |
|  | WLPTGTLLV | 26,47 | WB |
|  | MLWCKDGHV | 27,64 | WB |
|  | AMQTMLFTM | 29,18 | WB |
|  | WLTNIFGTV | 29,49 | WB |
|  | SLAIDAYPL | 30,08 | WB |
|  | FLPGVYSVI | 30,71 | WB |
|  | YVFCTVNAL | 32,02 | WB |
|  | KMVSLLSVL | 33,15 | WB |
|  | VMAYITGGV | 34,74 | WB |
|  | RIMASLVLA | 41,28 | WB |
|  | SLPSYAAFA | 42,23 | WB |
|  | SLIYSTAAL | 42,67 | WB |
|  | IIWFLLLSV | 42,84 | WB |
|  | FLYENAFLP | 43,34 | WB |
|  | GVFCGVDAV | 44,69 | WB |
|  | NMLRIMASL | 46,48 | WB |
|  | CLLNRYFRL | 46,52 | WB |
|  | AIMTRCLAV | 47,18 | WB |
|  | LMWLIINLV | 48,1 | WB |
|  | TIWFLLLSV | 49,39 | WB |
|  | GLVAEWFLA | 52,09 | WB |
|  | NTFSSTFNV | 53,97 | WB |
|  | RILGAGCFV | 55,38 | WB |
|  | FSASTSAFV | 56,04 | WB |
|  | GLALYYPSA | 56,13 | WB |
|  | MLFTMLRKL | 57,54 | WB |
|  | QLLFVVEVV | 58,87 | WB |
|  | VLSTFISAA | 61,11 | WB |
|  | SVFNICQAV | 61,15 | WB |
|  | VMYASAVVL | 64,38 | WB |
|  | VLQAVGACV | 69,01 | WB |
|  | CLTPVYSFL | 69,07 | WB |
|  | IVDTVSALV | 69,15 | WB |
|  | ILAYCNKTV | 69,62 | WB |
|  | FVLALLSDL | 70,4 | WB |
|  | FLMSFTVLC | 70,8 | WB |
|  | LQNNELSPV | 75,31 | WB |
|  | YASAVVLLI | 80,2 | WB |
|  | LLTILTSLL | 82,75 | WB |
|  | VLLSMQGAV | 86,01 | WB |
|  | KLLKSIAAT | 92,07 | WB |
|  | KLQFTSLEI | 95,1 | WB |
|  | RLSFKELLV | 99,68 | WB |
| ENVELOPE | FLLVTLAIL | 14,58 | WB |
|  | IVNSVLLFL | 74,79 | WB |
|  | SVLLFLAFV | 25,28 | WB |
|  | VLLFLAFVV | 30,41 | WB |
| ORF3A | WLIVGVALL | 26,18 | WB |
|  | NLLLLFVTV | 41,27 | WB |
|  | FVTVYSHLL | 67,33 | WB |
| MEMBRANE | FIASFRLFA | 48,46 | WB |
|  | FLFLTWICL | 59,1 | WB |
|  | TLACFVLAA | 25,06 | WB |
|  | WLLWPVTLA | 33,1 | WB |
| ORF6 | LIIMRTFKV | 76,91 | WB |
|  | SIWNLDYII | 51,71 | WB |
| ORF7a | FAFACPDGV | 33,12 | WB |
|  | FLALITLAT | 65,85 | WB |
| ORF7b | FLAFLLFLV | 6,21 | WB |
|  | YLCFLAFLL | 6,76 | WB |
|  | IIFWFSLEL | 18,42 | WB |
|  | ELSLIDFYL | 72,09 | WB |
| ORF8 | YVVDDPCPI | 59,49 | WB |

Suppl. Table 10 **Compared prediction of non-spike SB+WB by all algorithms.** The prediction of SB and WB non-spike epitopes predicted by NetMHC – Pan4.1 (<100nM) was assessed also with the indicated algorithms. Ranking in the first 16 positions in all methods is indicated. 12 epitopes predicted by NetMHC – Pan4.1 are found to be best ranking also in the other methods

| **PROTEIN** | **Peptide** | **NetMHC pan 4.1** | **ANN 4.0** | **SMM** | **MHC Flurry 2.0** |
| --- | --- | --- | --- | --- | --- |
| ORF 1ab | **FLAHIQWMV** | 1 | 1 | 5 | 2 |
|  | **ILFTRFFYV** | 2 | 2 | 2 | 5 |
|  | **FLLPSLATV** | 3 | 10 | 7 | 1 |
|  | **YLDAYNMMI** | 4 | 11 | 18 | 65 |
|  | **FLLNKEMYL** | 5 | 12 | 19 | 24 |
|  | **TLMNVLTLV** | 6 | 8 | 6 | 4 |
|  | **YLNTLTLAV** | 7 | 7 | 15 | 3 |
|  | **SMWALIISV** | 8 | 4 | 4 | 6 |
|  | **TMADLVYAL** | 9 | 6 | 14 | 8 |
|  | **LLLDDFVEI** | 10 | 3 | 10 | 35 |
|  | **VLWAHGFEL** | 11 | 13 | 9 | 20 |
|  | **ALLADKFPV** | 12 | 9 | 17 | 31 |
|  | **NLIDSYFVV** | 13 | 15 | 27 | 18 |
|  | **FVNEFYAYL** | 14 | 23 | 55 | 13 |
|  | **FLPRVFSAV** | 15 | 41 | 25 | 9 |
|  | **ALWEIQQVV** | 16 | 24 | 12 | 52 |
|  | **RIMTWLDMV** | 17 | 37 | 50 | 22 |
|  | **FLARGIVFM** | 18 | 22 | 28 | 34 |
|  | **YLATALLTL** | 19 | 5 | 13 | 7 |
|  | **YTMADLVYA** | 20 | 25 | 60 | 61 |
|  | **KLIEYTDFA** | 21 | 14 | 20 | 17 |
|  | **LMIERFVSL** | 22 | 32 | 32 | 27 |
|  | **FLNRFTTTL** | 23 | 27 | 46 | 10 |
|  | **ILTSLLVLV** | 24 | 45 | 44 | 32 |
|  | **MMISAGFSL** | 25 | 16 | 40 | 15 |
|  | **MLDMYSVML** | 26 | 57 | 45 | 147 |
|  | **QLFFSYFAV** | 27 | 36 | 24 | 42 |
|  | **KLNVGDYFV** | 28 | 29 | 36 | 29 |
|  | **ILHCANFNV** | 29 | 30 | 51 | 48 |
|  | **YLTNDVSFL** | 30 | 26 | 38 | 23 |
|  | **SLPGVFCGV** | 31 | 71 | 56 | 21 |
|  | **FVDGVPFVV** | 32 | 35 | 22 | 80 |
|  | **VLSFCAFAV** | 33 | 47 | 11 | 11 |
|  | **KLSYGIATV** | 34 | 20 | 23 | 12 |
|  | **VLAWLYAAV** | 35 | 46 | 30 | 26 |
|  | **WLMWLIINL** | 36 | 17 | 1 | 60 |
|  | **FVAAIFYLI** | 37 | 39 | 29 | 38 |
|  | **LLFLMSFTV** | 38 | 21 | 8 | 33 |
|  | **WMVMFTPLV** | 39 | 18 | 16 | 14 |
|  | **VMVELVAEL** | 40 | 40 | 33 | 36 |
|  | **YMPYFFTLL** | 41 | 19 | 21 | 47 |
|  | **NLSDRVVFV** | 42 | 33 | 35 | 28 |
|  | **VMCGGSLYV** | 43 | 50 | 43 | 16 |
|  | **TLIGDCATV** | 44 | 59 | 57 | 69 |
|  | **LLLTILTSL** | 45 | 38 | 37 | 49 |
|  | **AIFYLITPV** | 46 | 28 | 34 | 41 |
|  | **YLNSTNVTI** | 47 | 58 | 85 | 39 |
|  | **LVLSVNPYV** | 48 | 31 | 64 | 45 |
|  | **FLGRYMSAL** | 49 | 53 | 53 | 54 |
|  | **ILSPLYAFA** | 50 | 86 | 114 | 51 |
|  | **QLMCQPILL** | 51 | 64 | 63 | 106 |
|  | **LLADKFPVL** | 52 | 42 | 52 | 30 |
|  | **YVWKSYVHV** | 53 | 54 | 66 | 19 |
|  | **GLNDNLLEI** | 54 | 49 | 79 | 99 |
|  | **LLSAGIFGA** | 55 | 34 | 31 | 43 |
|  | **LQLGFSTGV** | 56 | 79 | 120 | 63 |
|  | **ILGTVSWNL** | 57 | 72 | 82 | 119 |
|  | **QMAPISAMV** | 58 | 63 | 77 | 37 |
|  | **FLALCADSI** | 59 | 83 | 61 | 71 |
|  | **LLMPILTLT** | 60 | 48 | 68 | 66 |
|  | **RLIDAMMFT** | 61 | 51 | 105 | 92 |
|  | **LLTNMFTPL** | 62 | 62 | 115 | 86 |
|  | **RQLLFVVEV** | 63 | 131 | 89 | 100 |
|  | **TQWSLFFFL** | 64 | 56 | 48 | 84 |
|  | **SLLSVLLSM** | 65 | 77 | 93 | 83 |
|  | **YLASGGQPI** | 66 | 43 | 74 | 56 |
|  | **VLLSVLQQL** | 68 | 85 | 87 | 59 |
|  | **KLKDCVMYA** | 69 | 73 | 78 | 107 |
|  | **TLVPQEHYV** | 70 | 95 | 90 | 25 |
|  | **FLNGSCGSV** | 71 | 66 | 54 | 72 |
|  | **RLANECAQV** | 72 | 120 | 99 | 112 |
|  | **TLGVYDYLV** | 73 | 75 | 59 | 74 |
|  | **AVIKTLQPV** | 75 | 105 | 117 | 58 |
|  | **FLKKDAPYI** | 75 | 105 | 117 | 58 |
|  | **NLLKDCPAV** | 77 | 65 | 65 | 87 |
|  | **KLVNKFLAL** | 79 | 93 | 128 | 73 |
|  | **SLENVAFNV** | 80 | 98 | 111 | 132 |
|  | **WLPTGTLLV** | 81 | 74 | 58 | 50 |
|  | **SQLGGLHLL** | 82 | 99 | 147 | 67 |
|  | **KLMPVCVET** | 83 | 52 | 72 | 122 |
|  | **MLWCKDGHV** | 84 | 81 | 62 | 75 |
|  | **WLTNIFGTV** | 86 | 136 | 142 | 62 |
|  | **SLAIDAYPL** | 87 | 67 | 47 | 88 |
|  | **FLPGVYSVI** | 88 | 106 | 102 | 57 |
|  | **NLWNTFTRL** | 89 | 125 | 109 | 115 |
|  | **YLITPVHVM** | 90 | 91 | 96 | 101 |
|  | **TLGVLVPHV** | 91 | 111 | 97 | 44 |
|  | **YVFCTVNAL** | 92 | 112 | 127 | 91 |
|  | **MLAKALRKV** | 93 | 122 | 86 | 77 |
|  | **KMVSLLSVL** | 94 | 101 | 107 | 79 |
|  | **TLSEQLDFI** | 95 | 76 | 131 | 46 |
|  | **KLNEEIAII** | 97 | 92 | 108 | 130 |
|  | **VMAYITGGV** | 98 | 94 | 95 | 40 |
|  | **KLNIKLLGV** | 100 | 110 | 69 | 105 |
|  | **FLRDGWEIV** | 101 | 104 | 70 | 95 |
|  | **LLDDFVEII** | 102 | 70 | 67 | 108 |
|  | **RIMASLVLA** | 103 | 116 | 155 | 127 |
|  | **SLPSYAAFA** | 104 | 113 | 116 | 125 |
|  | **SLIYSTAAL** | 105 | 84 | 101 | 103 |
|  | **IIWFLLLSV** | 106 | 61 | 26 | 134 |
|  | **VLGSLAATV** | 108 | 130 | 104 | 90 |
|  | **VLLAPLLSA** | 112 | 100 | 122 | 113 |
|  | **AIMTRCLAV** | 113 | 80 | 100 | 136 |
|  | **LMWLIINLV** | 116 | 44 | 3 | 53 |
|  | **SMQNCVLKL** | 117 | 140 | 137 | 146 |
|  | **MLSDTLKNL** | 120 | 108 | 136 | 85 |
|  | **GLALYYPSA** | 125 | 96 | 123 | 142 |
|  | **MLFTMLRKL** | 126 | 97 | 92 | 157 |
|  | **ALNLGETFV** | 128 | 114 | 119 | 131 |
|  | **VLSTFISAA** | 130 | 124 | 138 | 55 |
|  | **NLAKHCLHV** | 132 | 133 | 106 | 76 |
|  | **GLFKDCSKV** | 136 | 109 | 94 | 93 |
|  | **FLMSFTVLC** | 143 | 69 | 39 | 126 |
|  | **FITESKPSV** | 144 | 126 | 126 | 64 |
|  | **VLLSMQGAV** | 152 | 129 | 118 | 148 |
|  |  |  |  |  |  |
| ORF 3A | **LLYDANYFL** | 1 | 2 | 2 | 2 |
|  | **YLYALVYFL** | 2 | 1 | 1 | 1 |
|  | **ALSKGVHFV** | 3 | 3 | 3 | 3 |
|  | **TVYSHLLLV** | 4 | 4 | 8 | 4 |
|  | **ALVYFLQSI** | 5 | 5 | 7 | 5 |
|  | **WLIVGVALL** | 6 | 7 | 4 | 7 |
|  | **ALLAVFQSA** | 7 | 6 | 6 | 6 |
|  | **NLLLLFVTV** | 8 | 8 | 5 | 8 |
|  | **FVTVYSHLL** | 9 | 9 | 10 | 9 |
|  |  |  |  |  |  |
| ENVELOPE | **FLAFVVFLL** | 1 | 1 | 1 | 2 |
|  | **SLVKPSFYV** | 2 | 2 | 4 | 1 |
|  | **FLLVTLAIL** | 3 | 4 | 3 | 5 |
|  | **SVLLFLAFV** | 4 | 5 | 5 | 4 |
|  | **VLLFLAFVV** | 5 | 3 | 2 | 3 |
|  | **IVNSVLLFL** | 6 | 8 | 10 | 9 |
|  |  |  |  |  |  |
| GLYCOPR | **GLMWLSYFI** | 1 | 1 | 1 | 1 |
|  | **KLLEQWNLV** | 2 | 2 | 3 | 2 |
|  | **FVLAAVYRI** | 3 | 3 | 5 | 4 |
|  | **TLACFVLAA** | 4 | 7 | 10 | 3 |
|  | **SMWSFNPET** | 5 | 4 | 8 | 5 |
|  | **WLLWPVTLA** | 6 | 10 | 9 | 6 |
|  | **FIASFRLFA** | 7 | 8 | 13 | 7 |
|  | **FLFLTWICL** | 8 | 5 | 2 | 8 |
|  |  |  |  |  |  |
| NUCLEO | **LLLDRLNQL** | 1 | 1 | 1 | 1 |
|  | **GMSRIGMEV** | 2 | 2 | 2 | 2 |
|  |  |  |  |  |  |
| ORF 8 | **YIDIGNYTV** | 1 | 1 | 1 | 1 |
|  | **FLEYHDVRV** | 2 | 2 | 3 | 2 |
|  | **YVVDDPCPI** | 3 | 3 | 2 | 3 |
|  |  |  |  |  |  |
| ORF 6 | **HLVDFQVTI** | 1 | 1 | 2 | 1 |
|  | **SIWNLDYII** | 2 | 3 | 4 | 5 |
|  | **LIIMRTFKV** | 3 | 2 | 1 | 2 |
|  |  |  |  |  |  |
| ORF 7A | **KLFIRQEEV** | 1 | 1 | 4 | 1 |
|  | **FAFACPDGV** | 2 | 2 | 1 | 2 |
|  | **FLALITLAT** | 3 | 4 | 3 | 4 |
|  | **ILFLALITL** | 4 | 3 | 2 | 3 |
|  | **ELYSPIFLI** | 5 | 5 | 5 | 5 |
|  |  |  |  |  |  |
| ORF 7B | **FLAFLLFLV** | 1 | 1 | 1 | 1 |
|  | **YLCFLAFLL** | 2 | 3 | 3 | 2 |
|  | **IIFWFSLEL** | 3 | 4 | 7 | 5 |
|  | **ELSLIDFYL** | 4 | 10 | 13 | 9 |
